# Supplementary material for: Dengue vaccine acceptability in Peru: A mixed-methods study in two dengue-endemic Peruvian cities
Source: PLoS Negl Trop Dis. 2026 May 18;20(5):e0013572. doi: 10.1371/journal.pntd.0013572 (PMC13193613; doi:10.1371/journal.pntd.0013572)
Supplement: S1 File — (PDF) [file pntd.0013572.s001.pdf]

S1 Material: Data collection materials

ASOCIACION BENEFICA PRISMA
UNIVERSIDAD DE TULANE

Guía para grupos focales (GF) a residentes de comunidades de Iquitos y Piura, Perú

Título: "Examinar estrategias para aumentar aceptabilidad de vacuna y actividades para el control de dengue en el Perú"
Investigador principal: Dra. Valerie Paz Soldán
N° Versión / Fecha: Versión 2: 10 de enero 2022.

Al inicio de cada grupo focal a cada participante se le aplicará el consentimiento informado por escrito. Se pedirá consentimiento para 1) participación voluntaria, 2) ser audio-grabado y 3) fotografiado. Si alguien del grupo no acepta ser grabado o fotografiado, se tomarán notas detalladas.

1. **INTRODUCCION: Presentación y explicación del objetivo del grupo focal.**
   - *Introducción de equipo de investigación*
   - *Objetivo de grupo focal:*
     1. Entender preocupaciones, posibles retos y motivaciones hacia una futura vacuna contra dengue
     2. Examinar estrategias para aumentar la aceptabilidad de las actividades de prevención y control del mosquito que ocasiona el dengue.
   - *Actividad grupal de calentamiento y presentación de participantes. Cada uno se presenta y da la siguiente información:*
     - Nombre (solo primer nombre o nombre por ser usado)
     - Edad
     - Ocupación
     - Número de hijos (rango de edades)
     - Ha tenido dengue (que sepa) o no
     - Ha tenido COVID o no
     - Plato favorito

2. **ACTIVIDAD/ROMPEHIELOS: Exploración de experiencias previas a programa de vacunas en niños, vacuna de COVID y programa de control vectorial.**
   Se pondrá un cartel en cada esquina del cuarto –SI, NO, NO SE, y TAL VEZ. Se leerán los comentarios y en base a los que se agrupan en cada esquina, se profundizarán con preguntas exploratorias como: "Cuénteme por qué sí…"

   "Rompehielos"
   - Me gusta bailar
   - Me gustan los animales
   - He tenido experiencias positivas con el sistema de salud

   Experiencia previa con dengue
   - He tenido dengue
   - Tuve dengue fuerte
   - Conozco a alguien cercano que tuvo dengue fuerte
   - Conozco a alguien cercano que falleció de dengue
   - Me preocupa que me de dengue (de nuevo) *(Carteles: Nada, un poco, más o menos, muchísimo)*

- Me preocupa que a mis hijos les de dengue (de nuevo) *(Carteles: Nada, un poco, más o menos, muchísimo)*

Experiencia previa con COVID

- He tenido COVID
- Un familiar cercano ha tenido COVID
- Un familiar cercano falleció de COVID

Experiencia previa con vacunación nueva (COVID) e infantiles

- A mi me han vacunado contra la COVID-19
  - *(Cambiar cartel para 0, 1, 2, 3 dosis)*
  - *Indagar: experiencias propias o cercanas – positivo y/o barreras*
  - *Indagar sobre complacencia, confianza, conveniencia, comunicación y/o contexto*
- Mis hijos (de edad elegible) han sido vacunados con por lo menos dos dosis
  - *(Cartel de 0, 1, 2, 3)*
  - *Indagar: experiencias propias o cercanas – positivo y/o barreras*
  - *Indagar sobre complacencia, confianza, conveniencia, comunicación y/o contexto*
- A mis hijos les vacunaron para las enfermedades infantiles
  - *(Cartel: SI, NO, NO ESTOY SEGURA, CREO QUE SI)*
  - *Preguntas: nombres de vacunas, experiencias, positivo/barreras*
  - *Indagar sobre complacencia, confianza, conveniencia, comunicación y/o contexto*

Experiencia previa con vectores y su control

- Tengo muchos zancudos en mi casa
- Tengo muchos zancudos en mi barrio
- Cuando vienen a fumigar, siempre dejo que entren
  - *Preguntas: Hay circunstancias en las que no dejan? Explicar.*
  - *Indagar sobre complacencia, confianza, conveniencia, comunicación y/o contexto*
- Cuando vienen a abatizar (tratar el agua con insecticida), siempre dejo que lo hagan.
  - *Preguntas: Hay circunstancias en las que no dejan? Explicar.*
  - *Indagar sobre complacencia, confianza, conveniencia, comunicación y/o contexto*

3. **DISCUSION: Percepciones sobre una nueva vacuna contra DENGUE**

Ahora vamos a imaginar que ya existe una vacuna disponible contra dengue en su comunidad, en otras palabras, imaginemos que ya llegaron las vacunas contra dengue. Sabemos que todavía no existe, pero queremos entender cómo lo percibirían personas en su comunidad – no sólo su opinión personal!

- ¿Qué les gustaría saber o conocer sobre esta vacuna para tomar una decisión sobre si vacunarse?
  - *Preguntar: Confianza*

- ¿Piensan que uds se vacunarían con la nueva vacuna contra dengue? ¿Vacunarían a sus hijos?
  - *¿Por qué sí o por qué no?*

- ¿Piensan que personas en su comunidad se vacunarían con la nueva vacuna contra dengue? ¿Vacunarían a sus hijos?
  - *¿Por qué sí o por qué no?*

- ¿Qué piensa que ayudaría a la gente en su comunidad en decidir vacunarse?
  - *Explorar si son temas de confianza, conveniencia, comunicación, complacencia*

- ¿Qué se puede hacer para mejorar la [*confianza, conveniencia… palabras de los participantes*]?

- ¿De qué manera se podría entregar de manera más rápida y eficiente la vacuna contra dengue?

- ¿Qué dificultades podrían encontrarse con la entrega de las vacunas contra dengue? ¿Cómo se podrían solucionar estas dificultades?

- ¿En qué grupo etario -- ya sea niño pre-escolar, escolar, adolescentes o personas adultas – piensa que aceptarían más fácilmente las vacunas?

    - Explicar por qué.
    - ¿Cuáles serían las ventajas y desventajas?

Variables a considerar
Explorar aspectos generales de la complacencia (sobre la percepción de riesgo a tener dengue), confianza (seguridad y eficacia de la vacuna y confianza en el personal de salud) y conveniencia (barreras/dificultades en el acceso) sobre la situación hipotética de que existe una nueva vacuna contra dengue que se quiere aplicar en la comunidad de los participantes.

4. **Comunicación: Fuentes de información relacionados con la vacuna.**
Ahora conversaremos de los medios de comunicación (puede ser radio, TV, o redes sociales u otros) que hay en su localidad o que ustedes conocen y que sirven o pueden servir para recibir información sobre vacunas y dengue. Nuevamente nos vamos a separar por grupos en base a las respuestas de algunas preguntas.

- ¿Qué medios de comunicación -- tipo radio, televisión o redes sociales – utiliza regularmente? Y en su comunidad, ¿qué medios son populares?

- ¿En qué medios de comunicación -- tipo radio, televisión o redes sociales -- prefieren informarse sobre la pandemia del COVID-19?

    - ¿Qué es lo que informan sobre la pandemia y sobre las vacunas?
    - ¿Cuánto confían en ese medio para su información de salud?
    - ¿Qué otros medios escucha? ¿Cómo cambian los mensajes en base a los medios? ¿Cómo decide en quién confiar?

5. **Contexto: Características sociodemográficas, origen étnico, religión.**
Sigamos hablando en el contexto que existiera una vacuna contra dengue.

- ¿Creen que exista personas en su comunidad que se rehusarían a esta vacuna o que la vacuna no les llegue?
    - ¿Quiénes son estas personas? ¿Por qué rehusarían?

- ¿Ustedes piensan que los líderes religiosos podrían influenciar en el rechazo o aceptación de la vacuna?
    - ¿Cómo y qué comunicaron sobre COVID?

- ¿Algunas veces han escuchado frases como "tengo fe en que solo me voy a curar y no necesito de tratamientos, medicamento o vacunas"?

- ¿Qué nos pueden comentar?

- ¿Habría alguna manera de convencerlos para que también tengan fe en la vacuna para curarse? ¿Cómo les explicarían ustedes? O, ¿a quién recurrirían si tuviesen dudas?

- ¿En su comunidad han ocurrido hechos o situaciones que podrían influir en la aceptación o rechazo de una futura vacuna contra dengue?
    - Cuéntenos sobre esto.

Variables a considerar:
Explorar si existen poblaciones o grupos étnicos que estarían en riesgo de ser marginados en la entrega de vacunas. Explorar el rol de la religión en la aceptación/rechazo de la vacuna y si han ocurrido algunos hechos o situaciones dentro de sus localidades que podrían generar rechazo de la vacuna.

6. Experiencias previas con vacunas contra dengue.
En esta última parte solo discutiremos sobre las experiencias pasadas que ustedes han tenido con una vacuna contra dengue o la información que escucharon de otros familiares o conocidos sobre esta vacuna contra dengue que se aplicó en el pasado.

- ¿Algunos de ustedes, o sus familiares o conocidos han tenido experiencia con la vacuna contra dengue en el pasado?
    - ¿Cómo fue esa experiencia?
    - ¿Creen que esas personas vuelvan a vacunarse?

7. Cierre.
"Bueno, estamos finalizando con la conversación, antes de dar por concluida la reunión me gustaría saber si alguien tiene algún comentario u opinión adicional sobre lo conversado. Algo que ustedes sientan que no ha podido ser dicho…". [Esperar comentarios y recibirlos o concluir].

Proyecto de investigación para aumentar la aceptabilidad de vacuna y las actividades para el control de dengue en el Perú.

Gracias por su interés en participar en nuestro estudio, a continuación empezaremos con las preguntas. Cabe indicar que no hay respuestas correctas e incorrectas y le rogamos encarecidamente que responda con la verdad, recordándole que la información recolectada será confidencial y no daremos a conocer sus datos personales.

I. DATOS DE LA ZONA DE ESTUDIO, EL ENTREVISTADOR Y LA ENCUESTA

1 Site
- ○ Iquitos
- ○ Piura

2 Zona de Intervención
- ○ Zona control
- ○ Zona intervención

3 Nombres y apellidos del entrevistador

____

4 Código del entrevistador

____

5 Fecha de entrevista

____

6 Hora de inicio

____

7 Distrito

____

(En el caso de Batanes, poner Batanes)

8 Centro Poblado - Zona - Manzana (Anote esta información de acuerdo al Google Maps)

____

(Ejemplo: 0001-00100-011C)

9 Lote o número del hogar que se OBSERVA

____

(Si no tiene lote, poner S/N y en la sección final de comentarios descrir caracteristicas generales del hogar (pisos, color de casa, referencia, etc.))

II. INFORMACION DEMOGRÁFICA

1 Nombres y apellidos del participante

____

(Del participante)

2 Fecha de nacimiento del participante

____

3 Edad del participante

____

(Corroborar la edad)

4 Sexo del participante (Observar)

- Femenino
- Masculino
- Otro

(Anotar de acuerdo a su apariencia física)

5 Estado civil actual del participante

- Soltero(a)
- Casado(a)
- Conviviente
- Viudo(a)
- Separado(a)
- Divorciado(a)
- No responde

6 Ocupación: ¿A qué se dedica la mayor parte del tiempo?:

- ama de casa
- estudiante
- administrador/empresario
- chofer
- profesional de salud o educación
- oficina
- obrero u operarios con calificación (construcción, maquinaria, carpintero)
- fuerzas armadas/policla
- trabajador no calificado
- independiente /calificado
- desempleado / jubilado
- Otro: especificar

6.1 Especificar: Ocupación. OTRO:

____

7 Nivel Educativo: ¿Hasta qué grado estudió?

- Primaria
- Secundaria
- Escuela Técnica
- Universidad

(Luego preguntar, cuántos aNos ha estudiado)

7.1 ¿Cuántos años de estudio realizó?

____

8 ¿Cuántas personas viven en esta casa?

____

9 ¿Cuántas familias viven en esta casa?

____

(Si comparten ambientes y alimentos todos los días se considera una sola familia)

10 Sobre las personas de su hogar. Marque la alternativa que corresponda. (LEER ALTERNATIVAS)

- [ ] En mi hogar hay alguna mujer embarazada
- [ ] En mi hogar hay alguna persona con alguna enfermedad crónica
- [ ] En mi hogar hay alguna persona con discapacidad flsica
- [ ] En mi hogar hay niños menores de 5 años
- [ ] En mi hogar hay adultos mayores
- [ ] En mi hogar tengo mascotas
- [ ] Ninguna de las anteriores

III. INFORMACIÓN SOCIOECONÓMICA

1 ¿Cuántos ambientes hay en esta casa?

____

2 De estos ambientes, ¿cuántos son usados para dormir?

____

3 ¿Qué material usan para cocinar?

- ○ No cocina
- ○ Leña
- ○ Carbón
- ○ Kerosene
- ○ Gas
- ○ Electricidad

(marcar material predominante)

4 ¿Con qué servicios cuenta en su casa? (LEER ALTERNATIVA)

- ☐ Ningún servicio
- ☐ Teléfono fijo
- ☐ Teléfono celular
- ☐ Internet fijo (cableado/satelital)
- ☐ Internet de celular/modem
- ☐ Cable
- ☐ Desague
- ☐ Agua potable (o agua red pública)

(Marcar todos los que corresponda)

5 ¿De dónde obtiene el agua para beber y preparar alimentos?

- ○ Lluvia
- ○ Pozo (natural)
- ○ Agua potable de otra casa (vecino, familiar, etc)
- ○ Agua potable (o de red pública)
- ○ Botellas (agua tratada)
- ○ Otro

(Mayoritariamente)

5.1 Otro: ¿De dónde obtiene el agua para beber y preparar alimentos?

____

(Describa)

6 ¿Qué tipo de desague tienen en casa?

- ○ Letrina
- ○ Desague con canales
- ○ Red pública de desague
- ○ No tiene
- ○ Otro

6.1 Otro: ¿Qué tipo de desague tienen en casa?

____

(Describa el tipo de desgue)

IV. EXPERIENCIA PROPIA DE COVID O DENGUE DEL PARTICIPANTE

1 ¿Usted ha tenido COVID?:

- ○ No
- ○ Sí
- ○ No sé / No estoy seguro(a)

1.1 ¿Fue COVID grave? :

- ○ No
- ○ Sí
- ○ No sé / No estoy seguro(a)

(Considerar el episodio más fuerte de COVID, que ha tenido)

1.2 Escala de severidad de COVID:

Con respecto a tu experiencia con COVID. En una escala del 0 a al 10, en donde 0 significa qué la experiencia con COVID fue leve o que tuvo síntomás leves y donde 10 significa qué la experiencia con COVID fue muy grave, o que estuvo hospitalizado.

RESPONDA LA SIGUIENTE PREGUNTA:

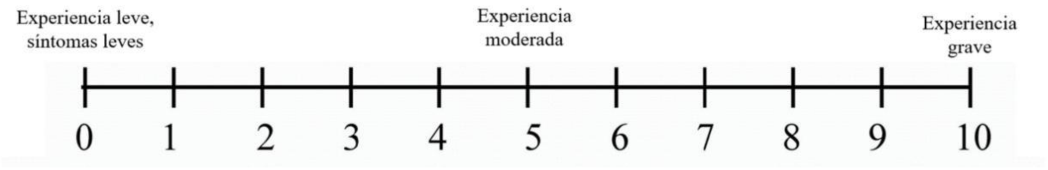

1 .3 ¿Cómo calificarías tu experiencia con COVID?
(Considerar el episodio más fuerte de COVID, que ha tenido)

○ 0 ○ 1 ○ 2 ○ 3 ○ 4 ○ 5 ○ 6 ○ 7 ○ 8 ○ 9 ○ 10 ○ No responde

2 ¿Usted ha tenido DENGUE?:

○ No
○ Sí
○ No estoy seguro(a)

2 .1 ¿Fue DENGUE grave o DENGUE hemorrágico? :

○ No
○ Sí
○ No estoy seguro(a)

(Considerar el episodio más fuerte de DENGUE, que ha tenido)

2.2 Escala de severidad de DENGUE:

Con respecto a tu experiencia con DENGUE?. En una escala del 0 a al 10, en donde 0 significa qué la experiencia con DENGUE? fue leve o que tuvo síntomás leves y donde 10 significa qué la experiencia con DENGUE? fue muy grave, o que estuvo hospitalizado.

RESPONDA LA SIGUIENTE PREGUNTA:

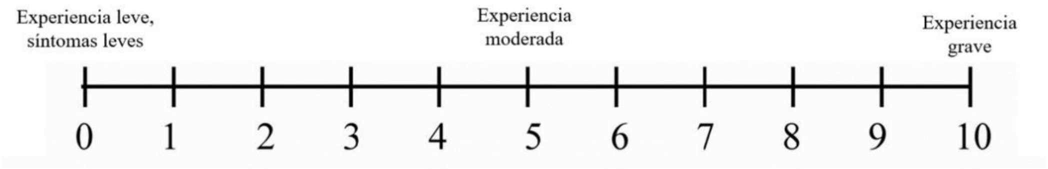

2 .3 ¿Cómo calificarías tu experiencia con DENGUE?
(Considerar el episodio más fuerte de DENGUE, que ha tenido)

○ 0 ○ 1 ○ 2 ○ 3 ○ 4 ○ 5 ○ 6 ○ 7 ○ 8 ○ 9 ○ 10 ○ No responde

2.4 ¿Hace cuánto tiempo tuvo esta experiencia con DENGUE? (en años)

____
(Considerar el episodio más fuerte de DENGUE, que ha tenido)

V1. EXPERIENCIA CERCANA DE FALLECIDOS POR COVID

1 ¿Usted ha tenidio algún familiar/AMIGA(O)/VECINA(O) cercano que falleció por COVID?:

○ No
○ Sí

1 .1 ¿Que relación tenía usted con estas personas que fallecieron con COVID? (LEER ALTERNATIVAS)

☐ Familiar cercano
☐ AMIGA(O) cercano
☐ VECINA(O) cercano
(ASEGURARSE DE PREGUNTAR POR SU FAMILIAR, AMIGO Y VECINO)

1.1.1 ¿.a Cuántos FAMILIARES cercanos fallecierón por COVID?

____

1.1.1.b En general. ¿Cuál era su grado de cercanía con su FAMILIAR O FAMILIARES que fallecieron por COVID?

○ Poco Cercanos
○ Algo cercano
○ Muy Cercanos
(Considerar el FAMILIAR más cercano)

1 .1.1.c En general. ¿Cuántas dosis de la vacuna contra COVID tenía su FAMILIAR O FAMILIARES que fallecieron por COVID?

○ Ninguna dosis
○ Una dosis
○ Dos dosis
○ Tres dosis (refuerzo #1)
○ Cuatro dosis (refuerzo #2)
○ No sé / No estoy seguro
(Considerar el FAMILIAR más cercano)

1.1.2 ¿.a Cuántos AMIGOS cercanos fallecierón por COVID?

____

1.1.2.b En general. ¿Cuál era su grado de cercanía con su AMIGA(O) O AMIGOS que fallecieron por COVID?

○ Poco Cercanos
○ Algo cercano
○ Muy Cercanos
(Considerar el AMIGA(O) más cercano)

1.1.2.c En general. ¿Cuántas dosis de la vacuna contra COVID tenía su AMIGA O AMIGOS que fallecieron por COVID?

○ Ninguna dosis
○ Una dosis
○ Dos dosis
○ Tres dosis (refuerzo #1)
○ Cuatro dosis (refuerzo #2)
○ No sé / No estoy seguro
(Considerar la AMIGA(O) más cercano)

1.1.3 ¿.a Cuántos VECINOS cercanos fallecierón por COVID?

____

1.1.3. En general. ¿Cuál era su grado de cercanía con su VECINA(O) O VECINOS que fallecieron por COVID?

- Poco Cercanos
- Algo cercano
- Muy Cercanos

(Considerar el VECINA(O) más cercano)

1 .1.3. En general. ¿Cuántas dosis de la vacuna contra COVID tenía su VECINA(o) O VECINOS que fallecieron por COVID?

- Ninguna dosis
- Una dosis
- Dos dosis
- Tres dosis (refuerzo #1)
- Cuatro dosis (refuerzo #2)
- No sé / No estoy seguro

(Considerar el VECINA(O) más cercano)

V2. EXPERIENCIA CERCANA CON COVID GRAVE O COVID SEVERO

1 ¿Usted ha tenido algún familiar/AMIGA(O)/VECINA(O) cercano que tuvo COVID GRAVE, COVID SEVERO, o estuvo hospitalizado por COVID?:

- No
- Sí

1 .1 ¿Que relación tenía usted estas personas que tuvieron COVID GRAVE/COVID SEVERO, o que estuvieron hospitalizados por esta enfermedad? (LEER ALTERNATIVAS)

- [ ] Familiar cercano
- [ ] AMIGA(O) cercano
- [ ] VECINA(O) cercano

(ASEGURARSE DE PREGUNTAR POR SU FAMILIAR, AMIGO Y VECINO)

1 .1.1. ¿Cuántos FAMILIARES cercanos han tenido COVID GRAVE/COVID SEVERO o han estado hospitalizado por esta enfermedad?

____

1 .1.1. En general. ¿Cuál era su grado de carcanía con su FAMILIAR O FAMILIARES que han tenido COVID GRAVE/COVID SEVERO o que han estado hospitalizado por esta enfermedad?

- Poco Cercanos
- Algo cercano
- Muy Cercanos

(Considerar el FAMILIAR más cercano)

1.1.1. Escala de severidad de COVID de su FAMILIAR:

Con respecto a la experiencia de COVID de tu FAMILIAR. En una escala del 0 a al 10, en donde 0 significa qué la experiencia con COVID fue leve o que tuvo síntomás leves y donde 10 significa qué la experiencia con COVID fue muy grave, o que se estuvo hospitalizado.

RESPONDA LA SIGUIENTE PREGUNTA:

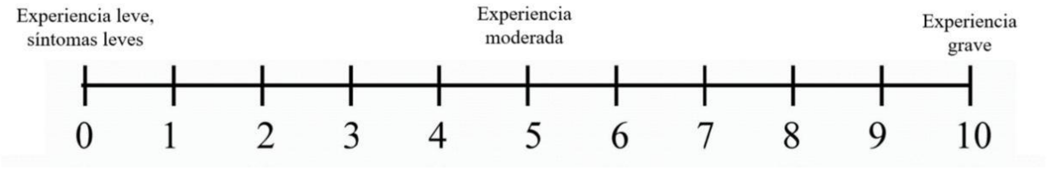

1 .1.1¿dCómo calificarías la experiencia de tu FAMILIAR con COVID GRAVE o COVID SEVERO?
(Considerar el FAMILIAR más cercano)

○ 0 ○ 1 ○ 2 ○ 3 ○ 4 ○ 5 ○ 6 ○ 7 ○ 8 ○ 9 ○ 10 ○ No responde

1 .1.2¿aCuántos AMIGOS cercanos han tenido COVID GRAVE/COVID SEVERO o han estado hospitalizado por esta enfermedad? ________________________________

1 .12Ebn general. ¿Cuál era su grado de carcanía con su AMIGA(O) O AMIGOS que han tenido COVID GRAVE/COVID SEVERO o que han estado hospitalizado por esta enfermedad?

○ Poco Cercanos
○ Algo cercano
○ Muy Cercanos
(Considerar la AMIGA(O) más cercano)

1.1.2E.cscala de severidad de COVID de su AMIGA(O):

Con respecto a la experiencia de COVID de tu AMIGA(O). En una escala del 0 a al 10, en donde 0 significa qué la experiencia con COVID fue leve o que tuvo síntomás leves y donde 10 significa qué la experiencia con COVID fue muy grave, o que se estuvo hospitalizado.

RESPONDA LA SIGUIENTE PREGUNTA:

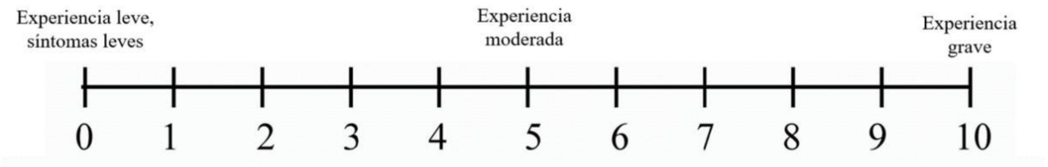

1 .1.2¿dCómo calificarías la experiencia de tu AMIGA(O) con COVID GRAVE o COVID SEVERO?
(Considerar la AMIGA(O) más cercano)

○ 0 ○ 1 ○ 2 ○ 3 ○ 4 ○ 5 ○ 6 ○ 7 ○ 8 ○ 9 ○ 10 ○ No responde

1 .1.3¿aCuántos VECINOS cercanos han tenido COVID GRAVE/COVID SEVERO o han estado hospitalizado por esta enfermedad? ________________________________

1 .13Ebn general. ¿Cuál era su grado de carcanía con su VECINA(O) O VECINOS que han tenido COVID GRAVE/COVID SEVERO o que han estado hospitalizado por esta enfermedad?

○ Poco Cercanos
○ Algo cercano
○ Muy Cercanos
(Considerar la VECINA(O) más cercano)

1.1.3E.cscala de severidad de COVID de su VECINA(O):

Con respecto a la experiencia de COVID de tu VECINA(O). En una escala del 0 a al 10, en donde 0 significa qué la experiencia con COVID fue leve o que tuvo síntomás leves y donde 10 significa qué la experiencia con COVID fue muy grave, o que se estuvo hospitalizado.

RESPONDA LA SIGUIENTE PREGUNTA:

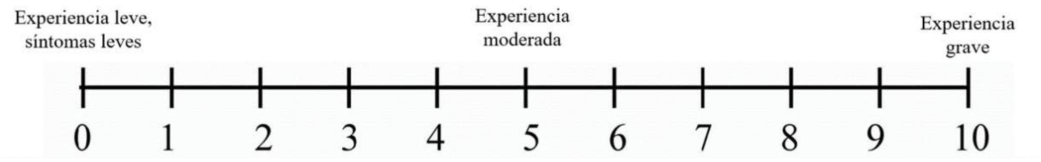

1 .1.3¿.dCómo calificarías la experiencia de tu VECINA(O) con COVID GRAVE o COVID SEVERO?
(Considerar la VECINA(O) más cercano)

○ 0 ○ 1 ○ 2 ○ 3 ○ 4 ○ 5 ○ 6 ○ 7 ○ 8 ○ 9 ○ 10 ○ No responde

VI1. EXPERIENCIA CERCANA DE FALLECIDOS POR DENGUE

1 ¿Usted ha tenidio algún familiar/AMIGA(O)/VECINA(O) cercano que falleció por DENGUE?:

○ No
○ Sí

1 .1 ¿Que relación tenía usted con estas personas que fallecieron con DENGUE? (LEER ALTERNATIVAS)

☐ Familiar cercano
☐ AMIGA(O) cercano
☐ VECINA(O) cercano
(ASEGURARSE DE PREGUNTAR POR SU FAMILIAR, AMIGO Y VECINO)

1.1.1¿.aCuántos FAMILIARES cercanos fallecierón por DENGUE?

____

1.1.1Eb.n general. ¿Cuál era su grado de carcanía con su FAMILIAR O FAMILIARES que fallecieron por DENGUE?

○ Poco Cercanos
○ Algo cercano
○ Muy Cercanos
(Considerar el FAMILIAR más cercano)

1.1.2¿.aCuántos AMIGOS cercanos fallecierón por DENGUE?

____

1.1.2Eb.n general. ¿Cuál era su grado de carcanía con su AMIGA(O) O AMIGOS que fallecieron por DENGUE?

○ Poco Cercanos
○ Algo cercano
○ Muy Cercanos
(Considerar el AMIGA(O) más cercano)

1.1.3¿.aCuántos VECINOS cercanos fallecierón por DENGUE?

____

1 .1.3 En general. ¿Cuál era su grado de carcanía con su VECINA(O) O VECINOS que fallecieron por DENGUE?

- ○ Poco Cercanos
- ○ Algo cercano
- ○ Muy Cercanos

(Considerar el VECINA(O) más cercano)

VI2. EXPERIENCIA CERCANA CON DENGUE GRAVE O DENGUE SEVERO

1 ¿Usted ha tenido algún familiar/AMIGA(O)/VECINA(O) cercano que tuvo DENGUE GRAVE, DENGUE SEVERO, DENGUE HOMORRÁGICO o estuvo hospitalizado por DENGUE?:

- ○ No
- ○ Sí

1 .1 ¿Que relación tiene usted con estas personas que tuvieron DENGUE GRAVE/DENGUE SEVERO, DENGUE HOMORRÁGICO o que estuvieron hospitalizados por esta enfermedad? (LEER ALTERNATIVAS)

- ☐ Familiar cercano
- ☐ AMIGA(O) cercano
- ☐ VECINA(O) cercano

(ASEGURARSE DE PREGUNTAR POR SU FAMILIAR, AMIGO Y VECINO)

1 .1.1 ¿Cuántos FAMILIARES cercanos han tenido DENGUE GRAVE/DENGUE SEVERO, DENGUE HOMORRÁGICO o han estado hospitalizado por esta enfermedad? ________________________________

1 .1.1 En general. ¿Cuál era su grado de carcanía con su FAMILIAR O FAMILIARES que han tenido DENGUE GRAVE/DENGUE SEVERO, DENGUE HOMORRÁGICO o que han estado hospitalizado por esta enfermedad?

- ○ Poco Cercanos
- ○ Algo cercano
- ○ Muy Cercanos

(Considerar el FAMILIAR más cercano)

1.1.1 Escala de severidad de DENGUE de su FAMILIAR:

Con respecto a la experiencia de DENGUE de tu FAMILIAR. En una escala del 0 a al 10, en donde 0 significa qué la experiencia con DENGUE fue leve o que tuvo síntomás leves y donde 10 significa qué la experiencia con DENGUE fue muy grave, o que se estuvo hospitalizado.

RESPONDA LA SIGUIENTE PREGUNTA:

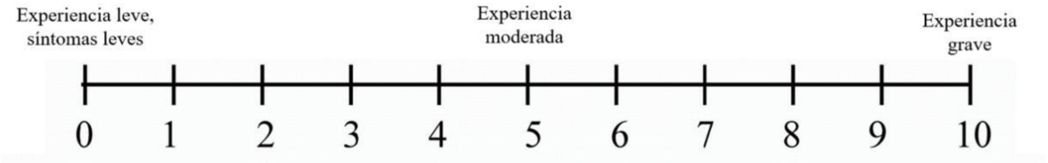

1 .1.1¿.dCómo calificarías la experiencia de tu FAMILIAR con DENGUE GRAVE, DENGUE SEVERO O DENGUE HOMORRÁGICO ?
(Considerar el FAMILIAR más cercano)

○ 0 ○ 1 ○ 2 ○ 3 ○ 4 ○ 5 ○ 6 ○ 7 ○ 8 ○ 9 ○ 10 ○ No responde

1 .1.2¿.aCuántos AMIGOS cercanos han tenido DENGUE GRAVE/DENGUE SEVERO, DENGUE HOMORRÁGICO o han estado hospitalizado por esta enfermedad? ________________________________

1 .1.2Eb.n general. ¿Cuál era su grado de carcanía con su AMIGA(O) O AMIGOS que han tenido DENGUE GRAVE/DENGUE SEVERO, DENGUE HOMORRÁGICO o que han estado hospitalizado por esta enfermedad?

○ Poco Cercanos
○ Algo cercano
○ Muy Cercanos
(Considerar la AMIGA(O) más cercano)

1.1.2E.cscala de severidad de DENGUE de su AMIGA(O):

Con respecto a la experiencia de DENGUE de tu AMIGA(O). En una escala del 0 a al 10, en donde 0 significa qué la experiencia con DENGUE fue leve o que tuvo síntomás leves y donde 10 significa qué la experiencia con DENGUE fue muy grave, o que se estuvo hospitalizado.

RESPONDA LA SIGUIENTE PREGUNTA:

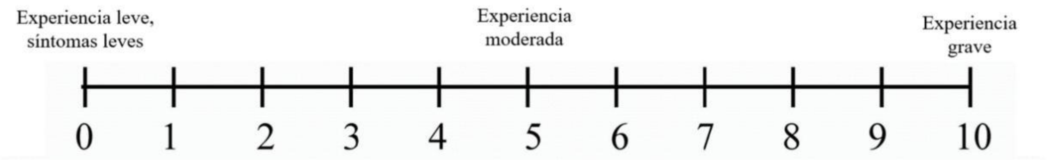

1 .1.2¿.dCómo calificarías la experiencia de tu AMIGA(O) con DENGUE GRAVE, DENGUE SEVERO O DENGUE HOMORRÁGICO
?
(Considerar la AMIGA(O) más cercano)

○ 0 ○ 1 ○ 2 ○ 3 ○ 4 ○ 5 ○ 6 ○ 7 ○ 8 ○ 9 ○ 10 ○ No responde

1 .1.3¿.aCuántos VECINOS cercanos han tenido DENGUE GRAVE/DENGUE SEVERO, DENGUE HOMORRÁGICO o han estado hospitalizado por esta enfermedad? ________________________________

1 .1.3Eb.n general. ¿Cuál era su grado de carcanía con su VECINA(O) O VECINOS que han tenido DENGUE GRAVE/DENGUE SEVERO, DENGUE HOMORRÁGICO o que han estado hospitalizado por esta enfermedad?

○ Poco Cercanos
○ Algo cercano
○ Muy Cercanos
(Considerar la VECINA(O) más cercano)

1.1.3 Escala de severidad de DENGUE de su VECINA(O):

Con respecto a la experiencia de DENGUE de tu VECINA(O). En una escala del 0 a al 10, en donde 0 significa qué la experiencia con DENGUE fue leve o que tuvo síntomás leves y donde 10 significa qué la experiencia con DENGUE fue muy grave, o que se estuvo hospitalizado.

RESPONDA LA SIGUIENTE PREGUNTA:

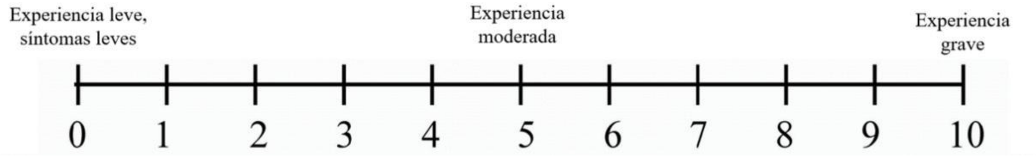

1 .1.3 ¿Cómo calificarías la experiencia de tu VECINA(O) con DENGUE GRAVE, DENGUE SEVERO O DENGUE HOMORRÁGICO ?
(Considerar la VECINA(O) más cercano)

○ 0 ○ 1 ○ 2 ○ 3 ○ 4 ○ 5 ○ 6 ○ 7 ○ 8 ○ 9 ○ 10 ○ No responde

VII. RENUENCIA A LA VACUNA DE COVID Y DENGUE

Experiencia de la vacunación contra COVID

1 Marcar la expresiones con las que usted está de acuerdo: (LEER ALTERNATIVAS)

- ○ Estoy a favor de la vacuna contra COVID
- ○ No estoy ni a favor ni en contra de la vacuna contra COVID
- ○ No estoy a favor de la vacuna contra COVID

1 .1 ¿Cuál es su principal razón por el cuál usted no esta a favor de las vacunas?

- ☐ No confío en la vacuna
- ☐ Va contra mi fe o creencia o religión
- ☐ No necesito la vacuna
- ☐ Otra razón

1.1.1 Otro: ¿Cuál es tu principal razón por el cuál usted no esta a favor de las vacunas?

____

2 ¿Usted cuántas dosis de la vacuna contra COVID tiene?:

- ○ Ninguna dosis
- ○ Una dosis
- ○ Dos dosis
- ○ Tres dosis (refuerzo #1)
- ○ Cuatro dosis (refuerzo #2)
- ○ No sé / No estoy seguro

3 ¿Qué dificultades tuvo usted al momento de aplicarse la vacuna contra COVID?

- ☐ No tuvo dificultades
- ☐ El tiempo de espera
- ☐ La cola era larga
- ☐ Las vacunas se acababan rápido
- ☐ Mucho sol/frio/lluvia
- ☐ Otra dificultad

(Marcar las 3 principales razones)

3.1 Anotar: ¿Qué otras dificultades tuvo usted al momento de vacunarse? ________________________________

---

4 ¿Qué es lo qué le dió a usted más confianza a usted en la vacuna contra el COVID?

- [ ] Ver al personal de salud vacunarse
- [ ] Ver que la OMS lo respaldaba
- [ ] Ver que el MINSA lo respaldaba
- [ ] Ver que la gente que se vacunaba no enfermaba gravemente o no moría
- [ ] Escuchar a algunas personalidades que lo recomendaban
- [ ] Otra razón:

(Marcar todos los que corresponda)

---

4 .1 Anotar: ¿Quién era esa persona o personas que hablaban de las vacunas y te generaba más confianza en la vacuna contra COVID? ________________________________

4.2 Anotar: ¿Qué otras acciones o situaciones le dieron a usted más confianza en la vacuna contra COVID? ________________________________

---

5 ¿Qué es lo qué le ocasionó a usted mayor desconfianza de la vacuna contra COVID?

- [ ] Salió muy rápido la vacuna o se produjo en un tiempo muy corto
- [ ] Lo que se decía en las noticias, redes sociales o grupos de WhatsApp sobre la vacuna
- [ ] Lo que hablaban mis vecinos o conocidos sobre las cosas que te pasaría si te aplicas la vacuna
- [ ] Las personas que abiertamente recomendaban no ponerse la vacuna
- [ ] Que sea obligatorio
- [ ] Ver a personal de salud o médicos que no la recomendaban
- [ ] Lo que decián los políticos o influencer sobre las vacunas
- [ ] Ningúno, yo confío/confiaba en la vacuna
- [ ] Otra razón:

(Marcar todos los que corresponda)

5.1 ¿Qué otras acciones o situaciones le generó a usted mayor desconfianza en la vacuna contra COVID? ________________________________

---

6 ¿Cuáles cree usted que sean las principales razónes por lo que las personas no se vacunan o no han completado sus dosis?

- [ ] Por falta de tiempo
- [ ] Desea ponerse, pero falta vacunas en su vecindario/distrito
- [ ] Experiencia previa negativa con vacunas (reacciónes adversas)
- [ ] Por tener una enfermedad o comorbilidad
- [ ] Por miedo a las agujas o a la aplicación de la vacuna
- [ ] Porque piensa que no va a enfermarse
- [ ] Por razónes religiosas (sello de la bestia, Dios me protege, etc.)
- [ ] Creen en teorías de conspiración y noticias falsas
- [ ] No creen en la efectividad de la vacuna
- [ ] Otra(s) razón(es)

(Marcar las 3 principales razones)

---

6 .1 Anotar: ¿Qué otras dificultades tienen las personas que no se quieren vacunas o no han completado sus dosis ________________________________

VIII. ESCALA DE ACTITUDES HACIA LA VACUNACION:

De acuerdo con las siguientes expresiones donde el 1 significa "Completamente en desacuerdo" y 5 significa "Completamente de acuerdo"; Responda lo siguiente:

		Completamente en desacuerdo	En desacuerdo	Neutral	De acuerdo	Completamente de acuerdo
1	Me siento seguro después de vacunarme	○	○	○	○	○
2	Confío en las vacunas frente a enfermedades infecciosas graves	○	○	○	○	○
3	Me siento protegido después de vacunarme	○	○	○	○	○
4	Aunque la mayoría de las vacunas parecen seguras, puede haber problemas que aun no hemos descubierto	○	○	○	○	○
5	Las vacunas pueden causar problemas imprevistos en los niños	○	○	○	○	○
6	Me preocupan los efectos desconocidos de las vacunas en el futuro	○	○	○	○	○
7	Las vacunas generan mucho dinero para las empresas farmacéuticas, pero no son realmente útiles para la gente normal	○	○	○	○	○
8	Las autoridades promueven la vacunación para obtener beneficios económicos, no por la salud de las personas	○	○	○	○	○
9	Los programás de vacunación son una gran estafa	○	○	○	○	○
1 0	La inmunidad propia dura más que una vacuna	○	○	○	○	○
1 1	La exposición natural a virus y gérmenes es la protección más segura	○	○	○	○	○
12	Estar expuesto a enfermedades de manera natural es más seguro para el sistema inmunológico que hacerlo medíante vacunas	○	○	○	○	○

IX. ESCALA DE RENUENCIA A LA VACUNACION:

De acuerdo con las siguientes expresiones donde el 1 significa "Completamente en desacuerdo" y 5 significa "Completamente de acuerdo"; Responda lo siguiente:

		Completamente en desacuerdo	En desacuerdo	Neutral	De acuerdo	Completamente de acuerdo
1	Las vacunas son importantes para mí salud	O	O	O	O	O
2	Las vacunas son efectivas	O	O	O	O	O
3	Estar vacunado es importante para la salud de las personas de mi comunidad	O	O	O	O	O
4	Todas las vacunas que ofrece el Ministerio de Salud son buenas	O	O	O	O	O
5	Las vacunas nuevas tienen más riesgo de efectos adversos que las antiguas	O	O	O	O	O
6	La información de las Vacunas que difunde el Ministerio de Salud es verdadera y confiable	O	O	O	O	O
7	Recibir la vacuna es la mejor manera de protegerme contra enfermedades	O	O	O	O	O
8	Generalmente pido que mi médico o el personal de salud me recomienda sobre las vacunas	O	O	O	O	O
9	Me preocupan los efectos adversos serios de algúnas vacunas	O	O	O	O	O
1 0	Yo no necesito vacunas para enfermedades que ya no se ven hoy en día.	O	O	O	O	O

X1. RENUENCIA A UNA FUTURA VACUNA CONTRA DENGUE PARA ADULTOS

Imaginémos: En este momento no hay ninguna vacuna contra dengue autorizada por el Ministerio de Salud. Dentro de este contexto vamos a IMAGINAR que próximamente estará disponible una nueva VACUNA CONTRA DENGUE en tu ciudad. Frente a este contexto, vamos a responder las siguientes preguntas.

1 ¿Si hubiera una vacuna disponible contra dengue aceptaría la vacuna si se le ofreciera?

- O No
- O Sí
- O No sé

2 ¿Si en la actualidad existiera una vacuna contra dengue? Usted consideraría lo siguiente: (LEER ALTERNATIVAS)

- ○ Me gustaría aplicármela lo antes posible
- ○ Me aplicaría cuando me la ofrezcan
- ○ No estoy seguro de lo que haré
- ○ pospondré (retrasaré) su aplicación
- ○ Me negaré a aplicármela
- ○ No sé

---

3 Describiría mi actitud hacia recibir una vacuna contra DENGUE cómo: (LEER ALTERNATIVAS)

- ○ Muy entusiasta
- ○ Bastante positivo
- ○ Neutral
- ○ Bastante preocupado(a)
- ○ En contra de la vacuna
- ○ No sé

---

4 ¿Si ya estuviera disponible para usted una vacuna contra DENGUE, que haría? (LEER ALTERNATIVAS)

- ○ Me la aplicaría tan pronto como pueda
- ○ Me la aplicaría cuando tenga tiempo
- ○ Retrasaría su aplicación
- ○ Evitaría aplicármela durante el mayor tiempo posible
- ○ Nunca me la aplicaría
- ○ No sé

---

5 Si mi familia o amigos estuvieran pensando en vacunarse contra el DENGUE, yo haría lo siguiente: (LEER ALTERNATIVAS)

- ○ Los animaría con entusiasmo
- ○ Los animaría
- ○ No les diría nada al respecto
- ○ Les pediría que retrasen su vacuna
- ○ Les sugerirla que No sé vacunen
- ○ No sé

---

6 Con respecto a recibir la vacuna contra DENGUE, yo me Describiría cómo: (LEER ALTERNATIVAS)

- ○ Ansioso porque quiero recibir la vacuna contra DENGUE
- ○ Dispuesto a recibir la vacuna contra DENGUE
- ○ No preocupado por recibir la vacuna contra DENGUE
- ○ No dispuesto a recibir la vacuna contra DENGUE
- ○ En contra de la vacuna contra DENGUE
- ○ No sé

---

7 Considero que recibir una vacuna contra DENGUE para mí es: (LEER ALTERNATIVAS)

- ○ Realmente importante
- ○ Importante
- ○ Ni importante ni no importante
- ○ No es importante
- ○ Realmente no es importante
- ○ No sé

---

8 Si la vacuna contra DENGUE estuviera disponible. ¿Usted estaría dispuesto a pagar por esta vacuna?

- ○ No
- ○ Sí
- ○ No sé/ Tal vez

---

8 .1 Apróximadamente cuanto estaría dispuesto a pagar usted por una futura vacuna contra dengue

____

(En soles. Si no responde colocar -1 (menos 1))

X2. RENUENCIA A UNA FUTURA VACUNA CONTRA DENGUE PARA MENORES DE EDAD

Sigámos imaginando: Aun no existe vacuna contra DENGUE autorizada por el Ministerio de Salud. Dentro de este contexto vamos a IMAGINAR que próximamente estará disponible una nueva VACUNA CONTRA DENGUE en tu ciudad. Frente a este contexto, vamos a responder las siguientes preguntas EN RELACIÓN A LA VACUNACIÓN EN LOS MENORES DE EDAD.

1 ¿En su hogar tiene algún hijo o algún menor de 16 años, de la cual usted tiene cierta responsabilidad de su cuidado?

- ☐ Sí. Mi hijo(a) menor de 5 años
- ☐ Sí. Mi hijo(a) de 6 años a 10 años
- ☐ Sí. Mi hijo(a) de 11 años a 15 años
- ☐ Sí. Mi familiar menor de 5 años
- ☐ Sí. Mi familiar de 6 años a 10 años
- ☐ Sí. Mi familiar de 11 años a 15 años
- ☐ No tengo hijos ni familiares menores de 16 años bajo mi cuidado

(INDAGAR SOBRE LAS EDADES DE MENORES DE 16 AÑOS Y LA RESPONABILIDAD QUE TIENE EN SU CUIDADO)

2 Si hubiera una vacuna disponible contra dengue para los menores de edad de su hogar: ¿Aceptaría la vacuna ,si se le ofreciera?

- ○ No
- ○ Sí
- ○ No sé

3 ¿Si en la actualidad existiera una vacuna contra dengue para los menores de edad de mi hogar? Usted consideraría lo siguiente: (LEER ALTERNATIVAS)

- ○ Me gustaría aplicársela lo antes posible
- ○ Lo aplicaría cuando me la ofrezcan
- ○ No estoy seguro de lo que haré
- ○ pospondría (retrasaré) su aplicación
- ○ Me negaría a aplicársela
- ○ No sé

4 Describiría mi actitud hacia la vacunación contra DENGUE de los menores de edad cómo: (LEER ALTERNATIVAS)

- ○ Muy entusiasta
- ○ Bastante positivo
- ○ Neutral
- ○ Bastante preocupado(a)
- ○ En contra de la vacuna
- ○ No sé

5 ¿Si ya estuviera disponible una vacuna contra DENGUE para los menores de edad, que haría? (LEER ALTERNATIVAS)

- ○ Lo aplicaría tan pronto como pueda
- ○ Lo aplicaría cuando tenga tiempo
- ○ Retrasaría su aplicación
- ○ Evitaría aplicárla durante el mayor tiempo posible
- ○ Nunca lo aplicaría
- ○ No sé

6 Si mi familia o amigos estuvieran pensando en vacunar a sus hijos menores de edad contra el DENGUE, yo haría lo siguiente: (LEER ALTERNATIVAS)

- ○ Los animaría con entusiasmo
- ○ Los animaría
- ○ No les diría nada al respecto
- ○ Les pediría que retrasen su vacuna
- ○ Les sugerirla que No sé vacunen
- ○ No sé

7 Con respecto a que los menores edad de mi hogar pueda recibir la vacuna contra DENGUE, yo me describiría cómo: (LEER ALTERNATIVAS)

- ○ Ansioso porque quiero que reciban la vacuna contra DENGUE
- ○ Dispuesto a que reciba la vacuna contra DENGUE
- ○ No preocupado por que puedan recibir la vacuna contra DENGUE
- ○ No dispuesto a que reciban la vacuna contra DENGUE
- ○ En contra de la vacuna contra DENGUE
- ○ No sé

8 Considero que recibir una vacuna contra DENGUE para los menores de edad de mi hogar es: (LEER ALTERNATIVAS)

- ○ Realmente importante
- ○ Importante
- ○ Ni importante ni no importante
- ○ No es importante
- ○ Realmente no es importante
- ○ No sé

9 Si la vacuna contra DENGUE para menores de edad estuviera disponible. ¿Usted estaría dispuesto a pagar por esta vacuna?

- ○ No
- ○ Sí
- ○ No sé/ Tal vez

9 .1 Apróximadamente cuanto estaría dispuesto a pagar usted por una futura vacuna contra dengue para menores de edad

____
(En soles. Si no responde colocar -1 (menos 1))

XI1. MODELOS DE LAS 5C DE LA RENUENCIA A LA VACUNACION

Ahora imaginemos que la vacuna de DENGUE ya EXISTE y que YA LLEGO al Perú. Frente a este contexto respondamos las siguientes preguntas.

Confianza

1 ¿Qué le gustaría saber de la nueva vacuna contra DENGUE antes de aplicarse?

- ☐ Sus efectos adversos y tratamientos cuando se presentan
- ☐ Si es efectivo
- ☐ Si puedo ponerme a pesar de tener comorbilidades
- ☐ Si puedo ponerme a pesar de que ya tuve dengue
- ☐ Cuantas dosis debe recibir
- ☐ Si está certificada o aprobada por el Ministerio de Salud/OMS-OPS
- ☐ Que laboratorio produjo las vacunas
- ☐ Saber en qué país fue producido la vacuna
- ☐ No me gustaría saber nada
- ☐ Otro:

(Marcar todos los que corresponda.)

1 .1 Redactar: ¿Qué otras cosas te gustaría saber de la nueva vacuna contra DENGUE antes de aplicarte

____

2 ¿Qué institución debiera dar a conocer que existe una nueva vacuna contra DENGUE, para qué a usted le genere mayor confianza? De las instituciónes que ha mencionado ¿Cuáles les genera mayor confianza?

- ☐ El Ministerio de Salud
- ☐ La Organización Mundial de la Salud
- ☐ La DIRESA/Geresa
- ☐ El centro de salud/Posta de Salud
- ☐ El Colegio Médico
- ☐ Otra institución:
- ☐ Ningua Institución
- ☐ No sabe /no responde

(Marcar hasta 3 alternativas y luego preguntar por el orden de la prioridad para cada respuesta dada)

---

2 .1 Otra ¿Qué institución debiera dar a conocer que existe una nueva vacuna contra DENGUE, para qué a usted le genere mayor confianza? De las instituciónes que ha mencionado ¿Cuáles les genera mayor confianza?

____

(Marcar hasta 3 alternativas y luego preguntar por el orden de la prioridad para cada respuesta dada)

---

2 .2 Del 1 a 3. Dónde 1 es la primera prioridad y 3 la tercera prioridad. ¿Qué orden de prioridad le asigna a usted a esta alternativa: Ministerio de Salud?

____

---

2 .3 Del 1 a 3. Dónde 1 es la primera prioridad y 3 la tercera prioridad. ¿Qué orden de prioridad le asigna a usted a esta alternativa: OrganizaciOn Mundíal de la Salud?

____

---

2 .4 Del 1 a 3. Dónde 1 es la primera prioridad y 3 la tercera prioridad. ¿Qué orden de prioridad le asigna a usted a esta alternativa: La DIRESA/GERESA?

____

---

2 .5 Del 1 a 3. Dónde 1 es la primera prioridad y 3 la tercera prioridad. ¿Qué orden de prioridad le asigna a usted a esta alternativa: El Centro de Salud/La Posta?

____

---

2 .6 Del 1 a 3. Dónde 1 es la primera prioridad y 3 la tercera prioridad. ¿Qué orden de prioridad le asigna a usted a esta alternativa: El Colegio médico del Perú?

____

---

2 .7 Del 1 a 3. Dónde 1 es la primera prioridad y 3 la tercera prioridad. ¿Qué orden de prioridad le asigna a usted a esta alternativa: Otra institución

____

---

3 Si la vacuna contra DENGUE estuviera autorizada por el Ministerio de Salud. ¿Usted se aplicaría?

- ○ No
- ○ Sí
- ○ No estoy seguro
- ○ No sabe , no responde

---

3 .1 ¿Cuáles serían los principales motivos, por las cuales usted NO se aplicaría la vacuna o no estaría seguro de aplicársela?

- ☐ No creo en las vacunas
- ☐ Primero buscaría información sobre la vacuna y en base a eso decidiría si vacunarme o no
- ☐ Primero esperarla que otras personas se apliquen y en base a eso decidiría si vacunarme o no
- ☐ Otra razón:

(Marcar todos los que corresponda)

3 .1.1¿Cuál sería el otro principal motivo, por las cuales usted NO se aplicaría la vacuna o no estaría seguro de aplicársela?

____

3.2 ¿Cuáles serían los principales motivos, por las cuales usted se aplicaría la vacuna?

- ☐ Para proteger mi salud y la de mis familiares
- ☐ Para proteger la salud de mis familiares
- ☐ Para no enfermarme gravemente por dengue
- ☐ Otra razón:

(Marcar todos los que corresponda)

3.2.1¿Cuál serían otro principal motivo, por las cuales usted se aplicaría la vacuna?

____

4 Existe algúna persona en su comunidad ya sea médico, autoridad, dirigente, sacerdote, periodista de radio o television, que le genere confianza sobre temás de salud?

- ○ No
- ○ Sí
- ○ No sabe/No responde

4 .1 ¿Quíen o quienes son estas personas? Puede mencionar varios nombres, pero tratar de señalar el nombre más importante

____

(Anotar el nombre de la persona más importante/influyente)

4 .2 Si algúno de estas personas que ha mencionado, les promocionara o informara sobre la nueva vacuna contra DENGUE. ¿Las personas de su comunidad tendrían más confianza en la vacuna?

- ○ No
- ○ Sí
- ○ No estoy seguro / No responde

XI2. PERCEPCION DE RIESGO SOBRE DENGUE

En qué medida usted está de acuerdo con las siguientes declaraciones?

		Totalmente en desacuerdo	En desacuerdo	Ni de acuerdo, ni en desacuerdo	De acuerdo	Totalmente de acuerdo
1	Asumo que me enfermaré de dengue	○	○	○	○	○
2	Creo que tengo una mayor probabilidad de contraer dengue en comparación con otros.	○	○	○	○	○
3	Todos en Iquitos/Piura corren un gran riesgo de contraer dengue.	○	○	○	○	○
4	Creo que corro riesgo de contraer dengue de nuevo.	○	○	○	○	○

XI3. PERCEPCIÓN DE SEVERIDAD SOBRE DENGUE

Por favor compruebe en qué medida usted está de acuerdo con las siguientes declaraciones.

		Totalmente en desacuerdo	En desacuerdo	Ni de acuerdo, ni en desacuerdo	De acuerdo	Totalmente de acuerdo
1	Dengue es una enfermedad seria	O	O	O	O	O
2	Las personas pueden morir si se enferman con dengue.	O	O	O	O	O
3	Las personas que tienen dengue tienen limitadas actividades sociales.	O	O	O	O	O
4	Una infección con dengue conduce a la pérdida de ingresos.	O	O	O	O	O
5	Una infección con dengue le cuesta mucho dinero al paciente.	O	O	O	O	O

XI4. CONVENIENCIA

1 Cómo se debería realizar la vacunación contra DENGUE a las personas de manera más eficiente?

- ☐ Vacunación casa por casa
- ☐ Vacunación en los colegios
- ☐ Vacunación en las plazas
- ☐ Otro:

(Marcar todos los que corresponda)

1.1 Qué otra estrategia se podría utilizar para qué la vacunación contra dengue sea la más efeciente

____

2 Si usted tuviera ¿Qué movilizarse desde su casa a un centro de vacunación ya sea caminando o utilizando algún tipo de trasporte ¿Cuánto tiempo de viaje estaría dispuesto a invertir para llegar al centro de vacunación?

- O Menos de una hora
- O Entre 1 hora y 2 horas
- O Entre 3 horas y 4 horas
- O más de 5 horas
- O La veces que sea necesario
- O No sabe / No responde

3 ¿Cuánto tiempo usted estaría dispuesto a esperar en las colas para aplicarse la vacuna contra DENGUE?

- O Menos de una hora
- O Entre 1 hora y 2 horas
- O Entre 3 horas y 4 horas
- O más de 5 horas
- O La veces que sea necesario
- O No sabe / No responde

4 Si llegara a ir a un centro de vacunación y no pudiera llegar a vacunarse por distintos motivos. ¿Cuántas veces más podría intentar regresar paravacunarse?

- O Una vez más
- O Dos veces más
- O Tres veces más
- O Las veces que sea necesario o buscaría otro lugar para vacunarme
- O No sabe / No responde

XI5. CONTEXTO

En el contexto que exista una nueva vacuna contra DENGUE en su comunidad, que haya sido aprobado por el Ministerio de Salud.

1 Si el Ministerio de Salud comenzara la campaña de vacunación contra DENGUE. ¿A qué personas, grupos, comunidades, sería dificil que el Ministerio de Salud logre vacunarlos?

- ☐ Los evangélicos o los religiosos
- ☐ Las personas que viven fuera de la ciudad o lugares peri-urbanos
- ☐ Las personas pobres
- ☐ Comunidades nativas / indígenas
- ☐ Otros:
- ☐ Ninguno/no sabe no responde

(arcar todos los que corresponda)

1 .1 Explique: A ¿Qué otras personas, grupos, comunidades, sería dificil que el Ministerio de Salud logre vacunarlos?

____

2 Creencias religiosas: En una escala del 1 al 7, Dónde 1 es "No es nada importante" y donde 7 es " extremadamente importante" ¿Que tan importante es la religión en tu vida?

- ○ Nada importante (Level 1)
- ○ Level 2
- ○ Level 3
- ○ Level 4
- ○ Level 5
- ○ Level 6
- ○ Es extremadamente importante (Level 7)

3 Creencias políticas: En política, muchas personas usan los términos "izquierda" y "derecha". En una escala del 1 al 7, Dónde 1 es "Fuertemente izquierdista" y donde 7 es "Fuertemente derechista". Cuando se trata de política, ¿Dónde te colocas en esta escala?

- ○ Fuertemente izquierdista (Level 1)
- ○ Level 2
- ○ Level 3
- ○ Level 4 (Centro)
- ○ Level 5
- ○ Level 6
- ○ Fuertemente derechista (Level 7)

XII1. RENUENCIA AL CONTROL VECTORIAL

Renuencia a la fumigación

1 ¿Cuándo fue la última vez que realizaron la fumigación contra los zancudos en su vecindario?

- ○ Hace menos de un año
- ○ Entre un año y dos años
- ○ Hace más de 3 años
- ○ Nunca han realizado la fumigación por aquí
- ○ No recuerda / no responde

2 De la última vez que realizaron la fumigación en su vecindario. ¿Usted permitió ingresar al personal de salud a su hogar todas las veces para la fumigación?

- ○ No. NUNCA PERMITE que ingresen a su hogar
- ○ Sí. SOLO PERMITE ALGUNAS VECES que ingresen a su hogar
- ○ Sí. SIEMPRE PERMITE que ingresen a su hogar todas las veces

2 .1 ¿Cuáles son las razónes principales por las cuales usted PERMITE ingresar siempre al fumigador a su hogar?

- ☐ Sí tengo tiempo
- ☐ Horario adecuado
- ☐ Confianza en el personal que realiza la actividad (Personas conocidas)
- ☐ Confianza en el producto (el producto es bueno)
- ☐ Mata los zancudos
- ☐ Otra razón:

(Marcar las 3 principales razones)

2.1.1Otra: ¿Cuál es la otra razón por la cual usted siempre permite fumigar? ____________________

2 .2 ¿Cuáles son las razónes principales por las cuales usted NUNCA PERMITE O SOLO PERMITE ALGUNAS VECES ingresar a su hogar para la fumigación?

- ☐ No tengo tiempo
- ☐ Horario inadecuado
- ☐ Desconfianza en el personal que realiza la actividad (se pierden las cosas)
- ☐ Desconfianza en el producto (el producto no sirve, solo es humo, es petróleo)
- ☐ Tengo un familiar vulnerable (incluye bebes, ancianos) y/o enfermo
- ☐ Tengo mi mascota
- ☐ Por el negocio que tengo (restaurante, etc.)
- ☐ Aparece más zancudos
- ☐ Dejan oliendo mis ropas y los alimentos
- ☐ Me obligan a salir de casa para la fumigación (a la fuerza/o es una obligación)
- ☐ Otra razón:

(Marcar hasta 3 razones más importantes)

2.2.1¿Qué otras razónes existe para qué la gente no permita fumigar en su hogar o no lo permita siempre? ____________________

2.3 Dejaron algún registro, marca o sticker cuando realizaron la fumigación?

- ○ No
- ○ Sí
- ○ No sé acuerda

2 .3.1Observar/verificar si cuenta con la marca o sticker de la fumigación.

- ○ Se verifica registro, marca o sticker de la fumigación
- ○ Menciona que ya se borró o ya fue retirado por diversos motivos
- ○ No permite realizar la verificación.

(Pedirle por favor que le permita verificar en la pared/puerta interna/externa del hogar)

3 ¿Cuál es su opinión sobre la calidad del producto que se utiliza para fumigar?

- ○ Percepción de mala calidad o el producto no funcióna
- ○ Se muestra indiferente sobre la calidad del producto (le da igual)
- ○ Percibe buena calidad o que el producto funcióna

3 .1 ¿Por qué piensa de esa manera sobre la calidad del producto utilizado para la fumigación?

- ☐ Solo es humo de petróleo y agua
- ☐ No mata a los zancudos
- ☐ Los zancudos se incrementan
- ☐ No fumigan bien
- ☐ No sé sobre el producto que utilizan
- ☐ Otra razón:

(Marcar todos los que corresponda)

3 .1.1Anotar otra razón, sobre las oponiones de la calidad del producto que se utiliza para fumigar, en el cual piensan que no es buena ____________________

4 ¿Sabe usted cuál es la RAZÓN PRINCIPAL de la FUMIGACIÓN en su hogar?

- ○ Para matar las larvas del zancudo
- ○ Para matar al zancudo adulto
- ○ Para matar a todos los insectos
- ○ No sirve para el zancudo
- ○ No sabe para qué sirve/no responde
- ○ Otro:

(MARCAR LA PRINCIPAL RAZON)

4 .1 Otro: ¿para qué sirve la fumigación?

____

5 ¿De qué manera se puede mejorar la fumigación?

- ☐ Que avisen con anticipación
- ☐ Que se utilice un producto que sea efectivo
- ☐ Que lo realicen en un horario adecuado
- ☐ Que la fumigación se realice correctamente
- ☐ Que utilicen un producto que no genere olor fuerte
- ☐ Que utilicen un producto que genere olor fuerte
- ☐ Que los trabajadores sean de confianza
- ☐ Que haya un lugar para descansar o sentarse mientras fumigan
- ☐ Otra recomendación:

(Marcar todos los que corresponda)

5.1 Otro: ¿De qué manera se puede mejorar la fumigación?

____

6 ¿Ha notado algún cambio en la fumigación del pasado con la fumigación de ahora?

- ☐ No. la fumigación es la misma
- ☐ Sí. la fumigación de antes era más fuerte
- ☐ Sí. la fumigación de ahora es más fuerte
- ☐ Otro cambio:

6.1 Otro: ¿Qué otro cambio a notado entre la fumigación de ahora y del pasado

____

7 ¿Qué debería pasar para qué usted diga qué la fumigación es buena?

- ☐ Que mate a los zancudos
- ☐ Que me genere picazón o me afecte ligeramente
- ☐ Que huela fuerte
- ☐ Que se realice en toda la casa
- ☐ Otro horario
- ☐ Otro:

(Marcar todos los que corresponda)

7 .1 Otro: ¿Qué debería pasar para qué usted piense que la fumigación ha sido buena?

____

XII2. HORARIO DE FUMIGACION: Si usted tuviera que recomendar algunos días y horarios ideales durante la semana, dónde el personal de salud podría venir a fumigar a su casa. 1 ¿ Qué días de la semana lo recomendarías? 2 ¿De esos días, cuales sería los horarios que recomendarías?

	En cualquier horario del día	De 5 am a 7 am	De 8 am a 11 am	De 12 m a 3 pm	De 4 pm a 7 pm	Después de las 8 pm	Ningún horario
1							

	Entre Lunes a Viernes (días de la semana)	☐	☐	☐	☐	☐	☐	☐
2	Sábado y domingo (Fines de semana)	☐	☐	☐	☐	☐	☐	☐

XIII1. RENUENCIA AL TRATAMIENTO DE AGUA.

Existe algunas ocasiones en las cuales el personal de salud visita las casas para repartir una bolsita o mallita o abate que se agrega en los recipientes de agua del hogar. Con respecto a la abatización responda las siguientes preguntas.

1 ¿Cuándo fue la última vez que realizaron la abatización en su vecindario?

- ○ Hace menos de 6 meses
- ○ Entre 6 meses a 1 año
- ○ Entre un año y dos años
- ○ Hace más de 2 años
- ○ Nunca han realizado la abatización por aquí
- ○ No recuerda

2 En su vecindario, normalmente en un año. ¿Cada cuánto tiempo vienen los abatizadores a los hogares?

- ○ Cada 1-2 meses
- ○ Cada 3 meses
- ○ Cada 4-5 meses
- ○ Cada 6-11 meses
- ○ Cada año
- ○ No lo sé
- ○ Otro momento:

2.1 Otro: ¿Cada ¿Cuánto tiempo vienen los abatizadores a los hogar.

____

3 De la última vez que realizaron la Abatización en su vecindario. ¿Usted permitió ingresar al personal de salud a su hogar todas las veces para la Abatización?

- ○ No. NUNCA PERMITE que ingresen a su hogar
- ○ Sí. SOLO PERMITE ALGUNAS VECES que ingresen a su hogar
- ○ Sí. SIEMPRE PERMITE que ingresen a su hogar todas las veces

3 .1 ¿Cuáles son las razónes principales por las cuales usted siempre no permite ingresar a su hogar para la Abatización o sólo permite algúnas veces?

- ☐ No tengo tiempo
- ☐ Horario inadecuado
- ☐ Desconfianza en el personal que realiza la actividad (se pierden las cosas)
- ☐ Desconfianza en el producto (el producto no sirve, solo es humo)
- ☐ Tengo un familiar vulnerable (incluye bebes, ancianos) y/o enfermo
- ☐ Tengo mi mascota
- ☐ Por el negocio que tengo (restaurante, etc.)
- ☐ Se siente el sabor de las comidas y el agua
- ☐ Otra razón:

(Marcar las 3 razones principales)

3 .1.1 Otra razón: ¿Cuáles son las razónes principales por las cuales usted siempre no permite ingresar a su hogar para la Abatización o sólo permite algúnas veces?

____

3.2 ¿Dejaron algún registro, marca o sticker cuando realizaron la Abatización?

- ○ No
- ○ Sí
- ○ No Recuerda

3.2.1 Observar/verificar si cuenta con la marca o sticker de la Abatización.

- ○ Se verifica registro, marca o sticker de la abatización
- ○ Menciona que ya se borró o ya fue retirado por diversos motivos
- ○ No permite realizar la verificación.

(Pedirle por favor que le permita verificar en la pared/puerta interna/externa del hogar)

---

3 .3 ¿Cuáles son las razónes principales por las cuales usted permite siempre ingresar al abatizador o solo permite ingresar algúnas veces al abatizador a su hogar?

- ☐ Tengo tiempo
- ☐ Horario adecuado
- ☐ Confianza en el personal que realiza la actividad
- ☐ Confianza en el producto (el producto no sirve, solo es humo)
- ☐ No se siente el sabor de las comidas y el agua
- ☐ No quiero los zancudos en mi hogar
- ☐ Para la prevención del dengue
- ☐ Es por mi salud/salud de mi familia
- ☐ Aunque no es efectivo, igual siempre permito fumigar mi hogar
- ☐ Otra razón:

(Marcar las 3 principales razones)

---

3 .3.1 Otro: ¿Cuáles son las razónes principales por las cuales usted permite siempre ingresar al abatizador o solo permite ingresar algúnas veces al abatizador a su hogar?

____

---

4 ¿Cuál es su opinión sobre la calidad del producto que utilizan en la Abatización?

- ○ Percepción de mala calidad o el producto no funcióna
- ○ Se muestra indiferente sobre la calidad del producto (le da igual)
- ○ Percibe buena calidad o que el producto funcióna

---

4 .1 ¿Por qué piensa de esa manera sobre la calidad del producto utilizado para la Abatización?

- ○ No mata a las larvas del zancudo
- ○ No mata a los zancudos
- ○ Los zancudos se incrementan
- ○ No abatizan bien
- ○ Otra razón:

4.1.1 Otra razón: ¿Por qué piensa qué la Abatización no funciona o funciona parcialmente?

____

---

5 ¿Sabe usted para cuál es la RAZÓN PRINCIPAL de la ABATIZACIÓN en su hogar?

- ○ Para matar las larvas del zancudo
- ○ Para matar al zancudo adulto
- ○ Para matar a todos los insectos
- ○ Para limpiar el agua para tomar o cocinar
- ○ No sirve para el zancudo
- ○ No sabe para qué sirve/no responde
- ○ Otra razón:

(MARCAR LA PRINCIPAL RAZON)

5.1 Otro: ¿Sabe usted para cuál es la RAZÓN PRINCIPAL de la ABATIZACIÓN en su hogar?

____

---

6 ¿De qué manera se puede mejorar la Abatización?

- ☐ Que avisen con anticipación
- ☐ Que se utilice un producto que sea efectivo
- ☐ Que lo realicen en un horario adecuado
- ☐ Que la abatización se realice correctamente
- ☐ Otra recomendación:

(Marcar todos los que corresponda.)

6 .1 Otro: ¿De qué manera se puede mejorar la Abatización? ______________________________

7 ¿Ha notado algún cambio en la Abatización del pasado con la Abatización de ahora?

- ☐ No. La abatización es la misma
- ☐ Sí. La abatización de antes era más fuerte
- ☐ Sí. La abatización de ahora es más fuerte
- ☐ Sí. Antes daban piedritas en una bolsa, ahora es polvo
- ☐ Ahora se utiliza menos cantidad del producto en la abatización
- ☐ Otro cambio:

7.1 Otro: ¿Ha notado algún cambio en la Abatización del pasado con la Abatización de ahora? ______________________________

8 ¿Qué debería pasar para qué usted diga qué la Abatización es buena?

- ☐ Que mate a las larvas
- ☐ Que mate a los zancudos
- ☐ Que huela fuerte
- ☐ Que se realice en toda la casa
- ☐ Que no afecte el sabor/olor al agua y/o comidas
- ☐ Otro horario:
- ☐ Otro:

(Marcar todos los que corresponda)

8.1 Otro: ¿Qué debería pasar para qué usted diga qué la Abatización es buena? ______________________________

9 ¿En qué tipo de recipientes colocan el abate?

- ☐ Baldes
- ☐ Bidones
- ☐ Tanque elevado
- ☐ Tanque bajo
- ☐ Pozo
- ☐ No sabe / no responde
- ☐ Otro:

9.1 Otro: ¿En qué tipo de recipientes colocan el abate? ______________________________

XIII2. HORARIO DE abatización: Si usted tuviera que recomendar algunos días y horarios ideales durante la semana, dónde el personal de salud podría venir a abatizar a su casa. 1 ¿ Qué días de la semana lo recomendarías? 2 ¿De esos días, cuales sería los horarios que recomendarías?

		En cualquier horario del día	De 5 am a 7 am	De 8 am a 11 am	De 12 m a 3 pm	De 4 pm a 7 pm	Después de las 8 pm	Ningún horario
1	Entre Lunes a Viernes (días de la semana)	☐	☐	☐	☐	☐	☐	☐
2	Sábado y domingo (Fines de semana)	☐	☐	☐	☐	☐	☐	☐

XIV1: RENUENCIA A LAS CAMPAÑAS DE RECOJO DE INSERVIBLES:

El recojo de inservibles se refiere al recojo de objetos como, por ejemplo, latas, botellas, llantas, etc. que usted puede tener en su jardln, patio, o en alguna parte de su casa, en el cual se puede acumular el agua y puede servir como nido de los zancudos. Con respecto a esto:

1 ¿En su vecindario realizan Campañas de recojo de inservibles?

- ○ No
- ○ Sí
- ○ No lo sé

1 .1 ¿Quienes realizan esta actividad?

- ☐ Personal de la municipalidad
- ☐ Personal de salud
- ☐ Los escolares
- ☐ Nosotros mismos lo hacemos
- ☐ Otro:

(Marcar todos los que corresponda)

1.1.1Otro: ¿Quienes realizan esta actividad?

____

1.2 Cada ¿Cuánto tiempo realizan esta actividad?

- ○ Cada 2 meses
- ○ Cada 3 meses
- ○ Cada 4 meses
- ○ Cada 6 meses
- ○ Cada año
- ○ No lo sé
- ○ Otro:

1.2.1Otro: ¿Cada ¿Cuánto tiempo realizan esta actividad?

____

2 ¿Usted participa o participaría en estas actividades o Campañas de recojo de inservibles si se dan o se darían?

- ○ No participo/participaría
- ○ Solo participo/participaría algunas veces
- ○ Sí, Síempre participo/participaría
- ○ No realizan campañas de recojo de inservibles

3 ¿Usted piensa qué las Campañas de recojo de inservibles funcionan?

- ○ No realizan campañas de inservibles
- ○ No Funcióna
- ○ Funcióna parcialmente / Solo sirve un momento
- ○ Sí funcióna

3 .1 ¿Por qué piensa qué las Campañas de recojo de inservibles no funciona o funciona parcialmente?

- ☐ Las personas no reconocen los criaderos
- ☐ Las personas no participan
- ☐ Solo realizan de vez en cuando
- ☐ Otra razón:

(Marcar todos los que corresponda)

3 .1.1Otro: ¿Por qué piensa qué las Campañas de recojo de inservibles no funciona o funciona parcialmente?

____

4 ¿Sabe usted cuál es la RAZÓN PRINCIPAL por las cuales se realiza las Campañas de recojo de inservibles?

- ○ Para eliminar los criaderos de zancudos
- ○ Para matar las larvas del zancudo
- ○ Para matar al zancudo adulto
- ○ Para matar a todos los insectos
- ○ No sirve para el zancudo
- ○ No sé para qué sirve
- ○ Otra razón

(MARCAR LA PRINCIPAL RAZON)

---

4 .1 Otro: ¿Sabe usted cuál es la RAZÓN PRINCIPAL por las cuales se realiza las Campañas de recojo de inservibles?

____

---

5 ¿De qué manera se puede mejorar las Campañas de recojo de inservibles?

- ☐ Que avisen con anticipación
- ☐ Que la municipalidad nos de un incentivo
- ☐ Que lo realicen en un horario adecuado
- ☐ Que toda la población participe
- ☐ Otra recomendación:
- ☐ Que los puntos de recojo sean cerca a mi casa

(Marcar todos los que corresponda)

5.1 Otro: ¿De qué manera se puede mejorar las Campañas de recojo de inservibles?

____

---

6 ¿Ha notado algún cambio en las Campañas de recojo de inservibles de ahora?

- ☐ No. las campañas de recojo de inservibles es la misma
- ☐ Sí, las campañas de recojo de inservibles de antes eran mejores
- ☐ Sí, las campañas de recojo de inservibles de ahora son mejores
- ☐ No sé / No realiza campaña de recojo de inservibles
- ☐ Otro cambio:

6.1 Otro: ¿Ha notado algún cambio en las Campañas de recojo de inservibles de ahora?

____

---

7 ¿Qué debería pasar para qué usted diga qué las Campañas de recojo de inservibles son buenas?

- ☐ Que la gente reconozca los criaderos
- ☐ Que disminuya a los zancudos
- ☐ Que se realice en todas las casas
- ☐ Que participe toda la población
- ☐ Otro horario
- ☐ Otro:

(Marcar todos los que corresponda)

7.1 Otro: ¿Qué debería pasar para qué usted diga qué las Campañas de recojo de inservibles son buenas?

____

---

8 ¿Qué tipo de inservible puede servir de criadero para el zancudo del dengue?

- ☐ Las latas
- ☐ Las botellas y/o sus tapas en el jardín
- ☐ Las llantas
- ☐ En un charco que se forma en cualquier parte de mi casa
- ☐ En las hojas de mis plantas
- ☐ Otro criadero:

(Marcar todos los que corresponda)

---

8 .1 Otro: ¿Qué tipo de inservible puede servir de criadero para el zancudo del dengue?

____

9 Para usted: ¿Qué día de la semana debería realizarse las campañas de recojo de inservibles?

- ☐ Lunes
- ☐ Martes
- ☐ Miércoles
- ☐ Jueves
- ☐ Viernes
- ☐ Sábado
- ☐ Domingo
- ☐ Ningún día

XIV2: BRIGADAS CONTRA DENGUE

Introduccion: Imagínense que se formen pequeños grupos en su comunidad - conformada por personas de su misma comunidad - que apoyaría con asegurarse que no hayan criaderos de zancudos en sus huertos o casas. Estas personas vendrían una vez cada 7-10 días para ayudarle a encontrar criaderos, enseñarles lo que encuentran, informarles sobre dengue, etc.

Frente a este contexto, responda las siguientes pregunta:

1 ¿Quiénes podrían tener esa función en su comunidad?

____

(SI NO RESPONDE, PONER NADIO/NINGUNO)

2 ¿Qué grupos comunitarios o vecinales hay en su comunidad que mobiliza o se comunica regularmente con personas en su comunidad?

- ☐ Qali Warma
- ☐ Asociaciones de Padres Escolares
- ☐ Comdedores Populares
- ☐ Dirigentes Vecinales
- ☐ Agentes comunitarios
- ☐ Otro:
- ☐ Nadie / No hay

2.1 Otro: ¿Qué grupos comunitarios o vecinales hay en su comunidad que mobiliza o se comunica regularmente con personas en su comunidad?

____

3 Si sus hijos o menores de edad quisieran participar como "asistentes en buscar y/o eliminar criaderos" en sus vecinadarios o casas de sus vecinos. ¿A partir de qué edad les podrían permitir participar en estas actividades?

____

(COLOCAR 1 SI NO APLICA O NO TIENE HIJO)

4 Si existiera una aplicación en el celular, que pudiera utilizarse para luchar contra el dengue y el zancudo. ¿Qué características debería tener este aplicativo para que las personas tengan mucho interés en utilizarlo?

____

XIV3: MEDIOS DE COMUNICACIÓN O REDES SOCIALES: Por donde se deberían difundir información sobre fumigación, abatización, recojo de invervibles y sobre campañas de vacunación contra dengue.

Por ejemplo. Si el Ministerio de Salud o la posta de salud de su distrito. Tuviera que informar a la población sobre una próxima campaña de FUMIGACIÓN: ¿Qué tipo de medio de comunicación debería utilizar para que la información llegue a personas como ustes o sus vecinos? ¿Si tuviera que recomendar SOLO UN MEDIO de comunicación o estrategia de difución para que sus vecinos se enteren de una próxima campaña de FUMIGACIÓN? ¿Cuál recomendaría?

		Por la radio	Por la TV	Por redes sociales (incluye Facebook)	Por perifoneo	Que avisen casa por casa	Por Ningúno/No responde	Otro medio;
1	Fumigación	O	O	O	O	O	O	O
2	Abatización	O	O	O	O	O	O	O
3	Recojo de inservibles	O	O	O	O	O	O	O
4	Campañas de vacunación contra dengue	O	O	O	O	O	O	O

4 .1 Otro medio FUMIGACIÓN:

____
(Describa el otro tipo de medio de difusión)

4 .2 Otro medio ABATIZACIÓN:

____
(Describa el otro tipo de medio de difusión)

4 .3 Otro medio capaña de RECOJO DE INSERVIBLES:

____
(Describa el otro tipo de medio de difusión)

4 .4 Otro medio VACUNACIÓN:

____
(Describa el otro tipo de medio de difusión)

XV1. CONOCIMIENTO SOBRE EL ZANCUDO

1 ¿Usted ha visto zancudos en su hogar?

- O No
- O Pocos
- O Mas o menos
- O Bastantes

1.1 ¿En qué lugares de su hogar los ha visto?

- [ ] Debajo de la cama
- [ ] Lugares oscuros
- [ ] Baños
- [ ] Cocina
- [ ] Dormitorios
- [ ] Ventanas
- [ ] Puertas
- [ ] Debajo de mesas
- [ ] En el jardín o patio
- [ ] Partes bajas de paredes
- [ ] En otro lugar

(Marcar todos los que corresponda)

1.1.1Otro: ¿En qué lugares de su hogar los ha visto?

____

---

2 ¿Hay algúna hora del día o noche donde fastidían más?

- ○ No
- ○ Sí
- ○ No sé / No responde

2.1 ¿Qué horas del día?

- ○ Entre las 5 am a 10 am
- ○ Entre las 10 am a 1 pm
- ○ Entre la 1 pm a 4 pm
- ○ Entre las 4 pm y las 8 pm
- ○ Todo el día
- ○ Toda la noche
- ○ No sabe / No responde

---

3 ¿En qué meses del año pica más o fastidia más el zancudo del DENGUE?

- [ ] Enero
- [ ] Febrero
- [ ] Marzo
- [ ] Abril
- [ ] Mayo
- [ ] Junio
- [ ] Julio
- [ ] Agosto
- [ ] Setiembre
- [ ] Octubre
- [ ] Noviembre
- [ ] Diciembre
- [ ] Todos los meses pica igual
- [ ] No hay zancudo / no sabe

(Puede marcar hasta 4 meses, si lo considera el participante o todo el año o sólo en un periodo de terminado)

XV2. CONOCIMIENTO SOBRE EL DENGUE

1 ¿Sabe usted cómo se transmite el dengue?

- ○ No
- ○ Sí

---

1 .1 ¿Cómo cree que se enferma de dengue?

- ○ Picadura de zancudo infectado
- ○ Por agua sucia
- ○ Por bañarse en el rio
- ○ No sabe
- ○ Otras explicacións

---

1 .1.1Otra explicaciones: ¿Cómo cree que se enferma de dengue?

____

2 ¿Sabe usted qué tipo de zancudo transmite el dengue?

- ○ No
- ○ Sí

2 .1 ¿Cómo es este zancudo o cómo se llama?

- ○ Tiene manchitas blancas en las patas
- ○ No Sabe
- ○ Otro

2.1.1Otro: ¿Cómo es este zancudo o Cómo se llama?

____

3 Puede decirme ¿Cuáles son los signos y síntomás del dengue?

- ☐ Fiebre
- ☐ Escalofrios
- ☐ Cansancio
- ☐ Erupción
- ☐ Dolor de cabeza
- ☐ Dolor de cuerpo
- ☐ Dolor de los ojos
- ☐ Dolor en las articulaciónes
- ☐ Nausea y/o vómitos
- ☐ pérdida de apetito
- ☐ Diarrea
- ☐ Mareos
- ☐ Malestar en general
- ☐ Dolor abdominal
- ☐ No sabe
- ☐ Otro:

(Marcar todos los que corresponda)

3.1 Otro: Puede decirme ¿Cuáles son los signos y síntomás del dengue?

____

4 Puede decirme ¿Cuáles son los síntomás de emergencia del dengue?

- ☐ Sangrado/hemorragia
- ☐ Malestar fuerte
- ☐ Dolor abdominal estomacal
- ☐ Se mejora de la enfermedad y luego empeora
- ☐ No sé
- ☐ Otro:

4.1 Otro: Puede decirme ¿Cuáles son los síntomás de emergencia del dengue?

____

5 ¿Cómo cree Usted que se trata el dengue? ¿Cuál es el tratamiento?

- ☐ Tomar paracetamol
- ☐ Ir a establecimiento de salud
- ☐ No sé toma nada
- ☐ Antibióticos
- ☐ Tomar refrescos/frescos
- ☐ No hay tratamiento
- ☐ No sé
- ☐ Otro:

(Marcar todos los que corresponda)

5 .1 Otro: ¿Cómo se trata el dengue? ¿Cuál es el tratamiento?

____

6	¿Si sospecha que tiene DENGUE LEVE a donde acude?	☐ A un familiar<br>☐ A mis amigos<br>☐ A la farmacia de la esquina<br>☐ Me tomo algunas pastillas<br>☐ Voy a la posta/ a la clInica/ consulto con algún médico<br>☐ Voy al hospital<br>☐ No sabe/no responde<br>☐ Otro:<br>(Marcar todos los que corresponda)
6.1	Otro: ¿Si sospecha que tiene DENGUE LEVE a donde acude?	__________________________________
7	¿Si sospecha que tiene DENGUE SEVERO O DENGUE HEMORRÁGICO a donde acude?	☐ A un familiar<br>☐ A mis amigos<br>☐ A la farmacia de la esquina<br>☐ Me tomo algunas pastillas<br>☐ Voy a la posta/ a la clInica/ consulto con algún médico<br>☐ Voy al hospital<br>☐ No sabe/no responde<br>☐ Otro:<br>(Marcar todos los que corresponda)
7.1	Otro: ¿Si sospecha que tiene DENGUE SEVERO O DENGUE HEMORRÁGICO a donde acude?	__________________________________
8	Por favor dígame si lo siguiente es verdadero o falso: El dengue le puede dar a todos por igual.	○ Falso<br>○ Vedadero<br>○ No sé
9	¿Hay personas que se enferman más con dengue?	○ Sí<br>○ No<br>○ No sé
9 .1	¿Quiénes están más propensos al dengue?	○ Bebes/niNos<br>○ Adultos<br>○ Mujeres embarazadas<br>○ Personas de tercera edad/ancianos<br>○ No sé<br>○ Otro
9.1.1	Otro: ¿Quiénes están más propensos al dengue?	__________________________________
10	¿Qué puede hacer uno para protegerse contra el dengue?	☐ Evitar picaduras de zancudo<br>☐ Usar productos contra zancudos en casa<br>☐ Ponerse repelente<br>☐ Limpiar la casa<br>☐ Rociar/fumigar la casa<br>☐ Recoger inservibles<br>☐ Tapar recipientes de agua<br>☐ Quemar hojas u otros productos<br>☐ Usar mosquitero al dormir<br>☐ No sabe/no responde<br>☐ Otro<br>(Marcar todos los que corresponda)

1 0.1 Otro: ¿Qué puede hacer uno para protegerse contra el dengue? ____________________

XVI. COMPORTAMIENTOS PREVENTIVOS

1 ¿Hace usted algo para reducir la cantidad de zancudos en su casa?
- ○ No
- ○ Sí

1 .1 ¿Qué hace?
- ☐ Usa productos contra zancudos en casa
- ☐ Limpia la casa
- ☐ Evita pocitos de agua en o alrededor de la casa
- ☐ Quema hojas u otros productos
- ☐ Saca la basura del jardín/patio
- ☐ Otro:

(Marcar todos los que corresponda)

1 .1.1¿Qué tipo de productos utiliza reducir los zancudos en su casa?
- ☐ Petróleo
- ☐ Lejía
- ☐ Gallito
- ☐ Insecticida comercial / Comun
- ☐ Baygón
- ☐ Espiral
- ☐ Otro

1 .1.1Otro: ¿Qué tipo de productos utiliza reducir los zancudos en su casa? ____________________

1.1.2Otro: ¿Qué otra actividad realiza para reducir los zancudos? ____________________

XVII. PREGUNTAS ADICIONALES DEL HOGAR Y OBSERVACIÓN DEL HOGAR

1 Marque los artefactos y/o vehículos que tiene en casa (no marcar si no tiene)
- ☐ Refrigeradora
- ☐ Televisor
- ☐ DVD
- ☐ Computadora
- ☐ Equipo de Sonido y/o Radio
- ☐ Cocina elEctrica y/o Gas
- ☐ Lavadora
- ☐ Bicicleta
- ☐ Motocicleta
- ☐ Motocarro
- ☐ Triciclo de trabajo
- ☐ Carro
- ☐ Peque Peque

2 Observar: Materiales de las paredes exteriores
- ○ Madera/ calamina
- ○ Ladrillos / bloque
- ○ Concreto (tarrajeado)
- ○ Otro: Especificar

(marcar material predominante)

2 .1 Otro tipo de material de la pared exterior ____________________

3 Observar: Materiales de las paredes interiores

- Triplay/calamina
- Madera
- Ladrillos / bloque
- Concreto (tarrajeado)
- Otro: Especificar

(marcar material predominante)

3 .1 Otro tipo de material de la pared interior

____

4 Observar: Tipo de techo

- Techo abierto
- Techo cerrado
- Techo abierto/cerrado

(tipo de techo mAs predominante)

5 Observar: Material de techo

- Hojas (hirapay, paja, etc.)
- Calamina
- Eternit
- Ladrillo/Cemento
- Otro:

(marcar material predominante)

5 .1 Otro: Observar: Material de techo

____

6 Observar: Material del piso

- Tierra / arena
- Madera
- Cemento
- Mayólicas
- Otro

(marcar material predominante)

6 Otro: Observar: Material del piso

____

7 Observar: ¿Cómo son las ventanas?

- Abiertas
- Con tapas de madera
- Con rejas
- Con vidrio
- Con malla contra insectos
- No hay ventana

(tipo de ventana mAs predominante)

XVIII. NOTAS U OBSERVACIONES DE LA ENTREVISTA

1 Encuestador: For favor, anote en este campo, las observaciones o alguna información adicional respecto a la entrevista con el participante

____

Hora de finalización de encuesta

____
