# Supplementary material for: Dengue vaccine acceptability in Peru: A mixed-methods study in two dengue-endemic Peruvian cities
Source: PLoS Negl Trop Dis. 2026 May 18;20(5):e0013572. doi: 10.1371/journal.pntd.0013572 (PMC13193613; doi:10.1371/journal.pntd.0013572)
Supplement: S1 Table — (DOCX) [file pntd.0013572.s002.docx]

**S1 Table. Representative Quotes by Themes and Subcodes, in Spanish and English.**

| **Themes** | **Subcodes** | **Quotes** |  |
| --- | --- | --- | --- |
| **Section: Confidence** | |  |  |
| Availability of clear and transparent information on the vaccine | Vaccine side effects | *Nos imaginamos que es una vacuna que es buena para tu salud. ¿Quién no va a querer ponérselo? Pero siempre y cuando terminen sabiendo cuales son los síntomas que vamos a tener. ¿No? (FG1)*  *Y el miedo a la reacción de la vacuna. (FG1)*  *¿qué efectos tiene después de haber puesto la vacuna? (FG11)* | *We imagine that it’s a vaccine which is good for your health. Who wouldn't want to get it? But as long as they end up knowing what symptoms we’re going to have. Right? (FG1)*  *Fear of a reaction to the vaccine. (FG1)*  *What are the side effects after getting the vaccine? (FG11)* |
|  | Vaccine eligibility | *una persona que ha tenido dengue puede poner? (FG4)*  *¿si ya lo probaron con una persona que ha tenido dengue ya sea un severo no? (FG9)*  *¿Qué problemas causaría en una mujer gestante o una persona diabética o cualquier otra enfermedad que tenga la persona? (FG10)* | *Can a person who has had dengue get vaccinated? (FG4)*  *Have they tested it on a person who’s had dengue, including a severe case? (FG9)*  *What problems would it cause in a pregnant woman, or a diabetic person, or for any other disease a person may have? (FG10)* |
|  | Evidence from others | *Así, por ejemplo, en esto de COVID, cuando ya supe que todos están por recibir ya también me puse la vacuna. (FG1)*  *solo estoy esperando solamente un poquito a ver qué pasa, voy a ver qué pasa con los niños que están vacunando no. (FG2)*  *(COVID) Como era algo novedoso y encontró a la infraestructura de salud agresivo, entonces, las personas lógicamente ha habido temor (FG3)*  *(COVID) Porque a veces escucho y veo la noticia que les choca, porque son muy pequeños, están experimentando en ellos...no lo voy a poner, depende más adelante sí, pero que primero quiero ver los demás niños. (FG4)*  *Primero como yo he dicho, esperar que se pongan otros, para ver que reacciones tiene (FG5)*  *voy a esperar que se ponga toda la gente para hacer yo, pero no voy a arriesgar a ponerme primero (FG11)* | *For example, with the COVID vaccine, when I found out that everyone was going to receive it, I also got the vaccine. (FG1)*  *I’m just waiting a bit to see what happens, I’m going to see what happens to the children who are getting vaccinated. (FG2)*  *(COVID) Since it was something new and it struck the healthcare system aggressively, so logically people were afraid.*  *(COVID) Because sometimes I hear and read news that it bothers them, because they are very young. They are experimenting on them... I am not going to get the vaccine. It depends later, yes, but first I want to see the other children. (FG4)*  *First, like I said, wait for others to get vaccinated, and see what reactions they have. (FG5)*  *I’m going to wait for everyone else to get it before I do it, but I’m not going to risk getting it first. (FG11)* |
|  | Number of doses | *Si, porque tiene ya las tres vacunas, tres dosis, nunca una vacuna es tres dosis, solamente es dos. (FG1)*  *Dice que la tercera es la más fuerte, por eso yo todavía no me pongo. Ya tengo miedo. (FG1)*  *Suficientes con dos dosis para vacunarse. (FG2)* | *Yes, because he already has three vaccines, three doses, a vaccine is never three doses, it’s only two. (FG1)*  *They say the third dose is the strongest, that's why I haven't gotten it yet. I'm already scared. (FG1)*  *Two doses are enough to get vaccinated. (FG2)* |
|  | Science | *Porque yo siempre sé decir las vacunas protegen del mal que se están volviendo en estos tiempos. (FG1)*  *Para que, siempre se decir eso. Miren familia yo desde que he nacido, ha habido el sarampión, la viruela, tuberculosis, Todo eso ha habido ven hijos, a mis vecinos a todos les digo eso, es una mentira eso que digan yo no me voy a vacunar, porque esa vacuna me va a enfermar me va a matar, no, la vacuna protege, porque creen que las personas siguen vacunados, los doctores siguen estudiando, los biólogos están preparando para que sepan todos los procesos, así que yo me vacune, sí. (FG1)*  *para defendernos de la enfermedad y lógicamente tenemos que vacunarnos porque si no nos vacunados estamos expuestos a la enfermedad (FG2)* | *Because I always say that vaccines protect against the bad that’s happening in these times. (FG1)*  *For what? I always say this. “Look, family. Since I was born, there has been measles, smallpox, tuberculosis, there has been all that, see kids.” I tell my neighbors and everyone that. What they say is a lie- “I’m not going to get vaccinated, because that vaccine is going to make me sick, it is going to kill me,” The vaccine protects. Why do you think that people continue to be vaccinated, the doctors continue to study, the biologists are preparing so that they know all the processes, so I got vaccinated, yes. (FG1)*  *To protect ourselves from the disease and logically we have to get vaccinated, because if we are not vaccinated we are at risk for the disease (FG2)* |
|  | Immunity | *la vacuna tiene un determinado tiempo de inmunización (FG2)*  *Dependiendo de las garantías que me darían pues, inmunizado de por vida, pues me pongo. (FG2)*  *¿si me pusiera esto ya no me daría nunca más el dengue?, ¿No tendría los síntomas? (FG4)*  *Yo preguntaría si me va a proteger parcial o completamente. (FG8)*  *¿Qué tiempo se está haciendo poner la vacuna o es una sola durante cinco, diez años? (FG9)*  *¿Cuántas dosis deberíamos recibir por persona y que tiempo este actúa este medicamento en nosotros? (FG10)*  *¿Y cuál es la duración de la vacuna? ¿Qué tiempo me va a proteger de esa enfermedad? (FG11)*  *100% de la protección. (FG13)* | *Does the vaccine have a certain immunization time? (FG2)*  *Depending on the guarantees they would give me- if it’s immunization for life, then I would get it. (FG2)*  *If I get the vaccine, would I never get dengue again? Would I not have the symptoms? (FG4)*  *I want to know if it will protect me partially or completely. (FG8)*  *How long does it take to get the vaccine, or is it just one for five, ten years? (FG9)*  *How many doses does a person need and how long does this medicine last for us? (FG10)*  *And what’s the vaccines duration? How long will it protect me from this disease?*  *100% protection. (FG13)* |
| Experience with Dengue | Experience with Dengue | *Bueno, como Loretano, en algún momento he tenido el dengue. (FG2)*  *Se murieron varias personas que conozco, de dengue y otros se salvaron. Mi primo se salvó, se iba con sangrando y se salvó. (FG2)*  *yo creo que la vacuna del dengue, es algo muy esperado y creo que sería un boom ese suceso (FG2)*  *El año pasado un niño, hijo de un amigo falleció por dengue hemorrágico...Y me ha dejado pasmado porque realmente yo nunca he tenido dengue y esa noticia nos ha afectado, uno que tiene hijos… (FG3)*  *Porque ahora es diferente, porque las enfermedades van evolucionando y ya no son iguales yo creo y ahora la enfermedad es más fuerte, bueno esa es mi idea. (FG13)*  *Eso que yo me vacunaría por dengue, porque usted sabe que a diario o a diario hay bastante zancudos y a nosotros nos pican (FG13)* | *Well, as someone from Loreto, I’ve had dengue at some point. (FG2)*  *Several people I know died of dengue and others survived. My cousin survived, he had hemorrhagic dengue and he survived. (FG2)*  *I think the dengue vaccine is something that is highly anticipated and I think this event would be a boom. (FG2)*  *Last year a child, the son of a friend, died of hemorrhagic dengue... And it has left me stunned because I’ve never had dengue and so this news has affected us, as someone who has children. (FG3)*  *Because now it’s different, because the diseases are evolving and they are no longer the same, I think. Now the disease is stronger, well that’s what I think. (FG13)*  *I would get vaccinated against dengue, because you know that there are lots of mosquitoes every day and they bite us (FG13)* |
| Trusted Healthcare Professionals | Local trusted sources | *sabe quién más da confianza? el padre Raymundo, porque si te dice las cosas porque él, las sabe (FG1)*  *Un infectólogo, Luis. Él siempre daba, daba charlas a conocer del seguro de salud. (FG1)*  *“Well, here in Unión, we honestly don’t trust the authorities. What they promise are lies. Maybe they were telling me something that isn’t true.” (FG11)* | *Do you know who inspires the most confidence? Father Raymundo. If he tells you things, it’s because he knows them (FG1)*  *An infectious disease specialist, Luis. He always gave talks about health insurance. (FG1)*  *Well, here in Unión, we honestly don’t trust the authorities. What they promise are lies. Maybe they were telling me something that isn’t true. (FG11)* |
|  | National sources | *Lo primero que haría es, saber si el ministerio de salud ya lo aprobó. (FG2)*  *Yo me vacunaría, pero como les digo, ya teniendo respaldo de los entes que promueven la vacuna. (FG2)* | *The first thing I would do is find out if the Ministry of Health has approved it. (FG2)*  *I would get vaccinated, but as I said, if it already had the support of the entities that promote the vaccine. (FG2)* |
| **Section: Complacency** | |  |  |
| Reasons for Vaccinating | Self-perceived risk from past experience | *Yo si también me pusiera la vacuna, porque como dice, el joven, hay cuatro tipos de dengue y toda esa gente caso me pusiera porque le temería más que toda al dengue hemorrágico. Y fuera de eso ese dengue se presenta cada año o cada creciente que se vive acá en esta región. Entonces yo si me pusiera (FG3)*  *Pero cuando te cae el dengue hemorrágico ya te empiezas a preocupar. Ahí recién empiezas a tomar conciencia no es cosa de bromearse, con dengue (FG13)*  *el dengue ha sido de muchos años que ha ido viviendo, pero qué pasa ¿Con que nos curábamos con el dengue? con puro amargo y eso lo utilizamos todavía nosotros (FG13)* | *I would also get the vaccine. As the young man says, there are four types of dengue, and all those people. I would get it because I fear dengue hemorrhagic fever more than anything. And apart from that, dengue occurs every year or every flood that we experience here in this region. So I would get it (FG3)*  *But when you get dengue hemorrhagic fever, you start to worry. That's when you start to realize it's not a joke, with dengue. (FG13)*  *Dengue has been around for many years, but what did we do? What did we use to cure dengue? Pure bitters, and we still use them. (FG13)* |
|  | Protecting the health of children | *Si durante dos años que ha pasado la pandemia ya, la gran mayoría fueron adultos y no ha afectado a los niños ¿Para que necesitamos vacunar a los niños ahora? (FG2)*  *Que es una preocupación como papá, padre de familia, tengo tres niños, cuatro niños son menores de edad y me preocupa de mi niño de cuatro añitos que va al jardín y no está vacunado. (FG2)*  *Y yo no estaba de acuerdo en mi hijo que me lo vacunen porque ellos son niños y ellos desarrollan defensas, ¿ya? Ellos desarrollan defensas, como están en todo crecimiento y no me parecía que le pongan vacuna a ellos (FG6)*  *Bueno para protegerle de su salud. (FG10)*  *es una responsabilidad como padre para vacunarse, para que ellos crezcan sano y fuerte con su vacuna de nacimiento. (FG11)*  *Si viene por el centro de salud si, como todas las vacuna protegerían a los niños. (FG12)*  *Bueno para mí que sería que primero vacunarían a los más pequeñitos. Para evitar esa enfermedad del dengue. (FG14)* | *If, during the two years that the pandemic began, the vast majority of cases were adults and it has not affected children, why do we need to vaccinate children now? (FG2)*  *It’s a concern as a father, a family man. I have three children, four of them are minors and I worry about my four-year-old son who goes to kindergarten and is not vaccinated. (FG2)*  *And I didn't agree with my son being vaccinated because they’re children and they develop defenses, right? They develop defenses as they are growing and I didn't think we should vaccinate them. (FG6)*  *Well, to protect your health. (FG10)*  *It’s a responsibility, as a parent, to get them vaccinated, so that they grow up healthy and strong with their childhood vaccines. (FG11)*  *If you come to the health center, yes. Like all vaccines, they protect children. (FG12)*  *Well, to me, they should vaccinate the little ones first, to avoid that dengue disease. (FG14)* |
|  | Work/ Travel and Policies | *me puse porque sé que voy a viajar a Calca. (FG1)*  *Porque sin esa vacuna no podíamos viajar. (FG1)*  *Me animé porque me habían dicho que tengo que tener, si no, no voy a poder trabajar. No, no voy a viajar. (FG1)*  *Mayormente los ancianos, como eran pensionistas les obligaban a vacunar, la vacuna quiera o no quieran porque pueden estar cobrando. (FG1)*  *porque en el colegio cuando entras te van a obligar a que los niños tengan sus vacunas (FG4)*  *Para jugar fútbol están pidiendo eso, si tú no tienes eso no puedes jugar. (FG5)* | *I got vaccinated because I know I'm going to travel to Calca. (FG1)*  *Because without the vaccine we can’t travel. (FG1)*  *I was encouraged because I’d been told that I have to have it otherwise I won't be able to work. No, I'm not going to travel. (FG1)*  *Mostly the elderly, since they were pensioners, were forced to get vaccinated, whether they wanted the vaccine or not because they would be collecting money. (FG1)*  *Because to enroll in school they will force you to get the children vaccinated. (FG4)*  *To play football they are asking for the vaccine, if you don't have it you can't play. (FG5)* |
| Reasons for Not Vaccinating | Low self-perceived risk | *pero ahora que ya hay la vacuna digamos del dengue creo pero porque me voy a poner si nunca eh tenido eso verdad, de repente si estoy de acuerdo con las personas que han tenido, pero particularmente para mí, que hasta ahora no eh tenido, no no me pondría porque todavía no, siento los síntomas (FG3)*  *Nunca me ha pasado, o sea no me he enfermado como para llamar a un médico. (FG6)*  *no tengo miedo porque te cae un dengue leve, ¿No? (FG13)*  *Tengo 64 años, hasta aquí yo no sé lo que es doctor, no sé lo que es una inyección y ya no me he caído gripe de esta enfermedad fuerte. (FG13)* | *But now that there is a vaccine, let's say for dengue, I think, but why am I going to get it if I've never had dengue, right? Maybe I agree with the people who have had it, but particularly for me, who until now still not has had dengue, I would not get it because I still don't feel the symptoms (FG3)*  *It's never happened to me. I mean, I’ve never gotten sick enough to call a doctor. (FG6)*  *I'm not afraid because you just get a mild dengue infection, right? (FG13)*  *I’m 64 years old, and I still don't know what a doctor is, I don't know what an injection is, and I haven't had the flu from this serious illness. (FG13)* |
|  | Religious views | *Por la simple razón que dice es el sello de la bestia. (FG1)*  *Por la religión no necesitan vacuna. (FG2)* | *For the simple reason that it says it is the sign of the beast. (FG1)*  *Because of religion, they do not need a vaccine. (FG2)* |
| Vector Control | Vaccine effect on vector control | *A mí, lo que yo te preguntaría si me vacunarías, entonces ¿ya no voy a matar mis zancudos (FG2)*  *Para esto del dengue es echarse el repelente o poner alguna pastilla o tomar aspiral ¿no? para protegernos, si deberíamos seguir usándolo, si en caso tenemos la vacuna colocada. (FG10)*  *He visto también que vienen por el sembrar de la chacra, de hecho, que ahí va a seguir habiendo zancudo ¿no? Entonces con la vacuna ya estaría protegido (FG10)* | *As for me, what I want to know is if I get vaccinated, then I no longer need to kill my mosquitoes? (FG2)*  *For dengue, we should put on repellent or take a pill or use a mosquito coil, right? To protect ourselves, we should continue doing this, even if we’ve been vaccinated. (FG10)*  *I’ve also seen that they come for the sowing of the fields, in fact, there will still be mosquitoes there, right? So with the vaccine they would be protected (FG10)* |
| **Section: Convenience** | |  |  |
| Physical Barriers | Long lines | *Vendían su cola. (FG1)*  *Si, es que la gente madrugaba, llevaba sus sillas y ponían delante y ellos vendían la cola 10 soles 20 soles. (FG1)*  *La cola más que todo, yo me demoré casi una hora en pasar. (FG2)*  *Lo único que, esperar la cola desde la noche hasta la madrugada. (FG16)* | *They sold their spot in line. (FG1)*  *People got up early, brought their chairs, put them in front of the line, and they sold their spot for 10 soles or 20 soles. (FG1)*  *The line more than anything, it took me almost an hour to get through. (FG2)*  *The only thing is that you have to wait in line from night until dawn. (FG16)* |
|  | Costs | *porque no solamente vas a estar en el hospital porque a veces tienes que comprar algo para el dolor de cabeza y previniendo mejor me pongo una vacuna que me cuesta 30 soles ya no voy a tener ese gasto (FG1)*  *generalmente las vacunas la dan el gobierno. No hay, no veo una vacuna que nosotros la podamos comprar. (FG2)*  *si es por el Estado la cantidad de plata que pagamos en impuestos tiene que ser gratis. (FG5)*  *prefiero salvar a mi familia, puedo pagar hasta lo que me dicen 50, 20 soles, lo pago pues, es para protegernos para salvarle la vida. (FG11)*  *el Estado ya vengan a vacunarlos por sus casas, las enfermeras, pero gratis, no les cobran pues. (FG14)*  *las vacunas son gratis, yo no pagaría. Tiene que ser gratis. (FG15)* | *Because not only are you going to be in the hospital, but sometimes you have to buy something for the headache and to prevention. Better if I get a vaccine that costs me 30 soles, then I will no longer have this expense. (FG1)*  *Generally, vaccines are provided by the government. There’s not- I don't know of a vaccine that we can buy. (FG2)*  *If it’s from the State, with the amount of money we pay in taxes it has to be free. (FG5)*  *I prefer to save my family, I can pay whatever they tell me to. 50, 20 soles, I’ll pay it. It’s to protect us, to save our lives. (FG11)*  *The State comes and vaccinates them at home with nurses. But for free, they don't charge them. (FG14)*  *Vaccines are free, I wouldn't pay. It should be free. (FG15)* |
| Improving Access | Ideas for rolling out the dengue vaccine | *Si, porque hay personas que trabajan, no tienen tiempo, pero los domingos si, en tal sitio y vamos. (FG1)*  *Cuando hacíamos campañas grandes con el INEI venían de repente les daban un refresco o venían para un menú, esas personas conociendo su idiosincrasia, como viven en su comunidad, mientras no les des algo no van a ir o no lo van hacer. (FG3)*  *Y viendo que es muy importante sensibilización sostenida no que vayan una vez y de ahí no aparezcan, para cambiar la estructura mental de las personas de la comunidad. Tiene que ser constante, hay que estar con ellos, experimentar. (FG3)*  *En ir a los barrios, a hacer charlas. (FG4)*  *Perifonear que se acerquen por favor (FG12)*  *Comunicar al medio de emisora local. (FG15)* | *Yes, because there are people who work, they don't have time to go. But on Sundays they can, in this place and we will go. (FG1)*  *When we did large campaigns with the INEI, they would come and give them a soft drink or a meal. These people know their idiosyncrasies, how they live in their community. If you don't give them something, they won't go or they won't do it. (FG3)*  *And seeing sustained awareness is very important, not just going once and then not returning. To change the mental structure of the people in the community it has to be constant, you have to be with them, experiment. (FG3)*  *Go to the neighborhoods and give talks. (FG4)*  *Broadcast that they should come near please. (FG12)*  *Communicate to the local broadcasting station. (FG15)* |
| **Section: Communication** | |  |  |
| COVID-19 Misinformation | Misinformation | *Dicen que murieron personas por vacunar (FG1)*  *Yo escuché que nos quieren implantar un chip (FG3)*  *él había escuchado esta vacuna a los 6 años va a morir. (FG5)*  *Los chinos están haciendo para disminuir la población. (FG6)*  *Que se van a morir, no van a poder tomar sus chela. (FG6)*  *si se vacunaba que se iba a volver estériles o algo así y ella no se ha puesto ni una vacuna (FG10)*  *Que bajaba la potencia sexual. (FG15)* | *They say people have died from the vaccine. (FG1)*  *I heard they want to implant a chip in us (FG3)*  *He heard people with this vaccine are going to die after 6 years. (FG5)*  *The Chinese are doing it to reduce the population. (FG6)*  *They are going to die, they won't be able to drink their beer. (FG6)*  *If you’re vaccinated you would become sterile or something like that, so she hasn't had a single vaccine (FG10)*  *It lowered sexual potency. (FG15)* |
| Vaccine Information | Sources of information | *Se prende la radio mientras estás haciendo tus cosas ahí. (FG1)*  *yo me informe por, por internet. (FG2)*  *Sí, algo, yo me informaba todo con respecto a esta enfermedad ahí en exitosa, escuchaba en exitosa, al doctor Massé (FG2)*  *me entro en el Facebook y en minutos libres (FG3)*  *porque no todo lo que sale en el internet es real y en las personas que ponen tampoco no son confiables. (FG6)*  *Yo no confío en la vacuna por... por motivos que vi en la noticias, o sea me deje guiar por las noticias (FG7)*  *Por eso yo me informo de la manera adecuada, escuchando al doctor, escucharlo por medio de la noticia es la manera más confiable para mí. (FG7)*  *La emisora pues, la emisora también pasa. (FG10)* | *The radio is on while you're doing your work. (FG1)*  *I get information from the internet. (FG2)*  *Yes, I informed myself about everything regarding this disease from Radio Exitosa, I listened to Dr. Massé on Radio Exitosa. (FG2)*  *I go on Facebook in my free time (FG3)*  *Because not everything that appears on the Internet is real and people who post are not trustworthy either. (FG6)*  *I don't trust the vaccine for reasons I saw on the news. I let myself be influenced by the news (FG7)*  *That's why I inform myself in the right way: listening to the doctor, listening to him through the news is the most reliable source for me. (FG7)*  *The radio station, well, the station as well. (FG10)* |
