# Supplementary material for: Dengue vaccine acceptability in Peru: A mixed-methods study in two dengue-endemic Peruvian cities
Source: PLoS Negl Trop Dis. 2026 May 18;20(5):e0013572. doi: 10.1371/journal.pntd.0013572 (PMC13193613; doi:10.1371/journal.pntd.0013572)
Supplement: S2 Table — (DOCX) [file pntd.0013572.s003.docx]

### **S2 Table. Convergence Matrix of Mixed-Methods Findings**

| **Theme/ Key Findings** | **Survey Findings** | **Focus Group Findings** | **Integration Conclusion** |
| --- | --- | --- | --- |
| **Confidence** | | | |
| Overall acceptance of hypothetical dengue vaccine | Acceptance rate of 81.9%. | Participants had a positive outlook for a future dengue vaccine, with participants describing it as highly anticipated and beneficial. | Strong convergence: High baseline confidence in both study populations. |
| Views influenced by experience with the COVID-19 vaccine or other vaccines | Decreased confidence associated to: unfavorable opinion of COVID-19 vaccine, short development time of the COVID-19 vaccine, and overall distrust in vaccines (VAX scale). | Confidence in the dengue vaccine reflected experience with the COVID-19 vaccine- participants wanted to know who created it, number of doses, and side effects. | Strong convergence: Broader vaccine trust influences dengue vaccine intentions. |
| **Complacency** | | | |
| Self- perceived risk from past experience | Belief that one can get dengue more than once and that one can die from dengue associated with vaccine acceptance. | Participants who had or knew someone with severe dengue were more vocal about wanting the vaccine to protect against severe disease, particularly for risk of hemorrhagic dengue. | Convergence: Perception of severity and previous experience with dengue associated with acceptance. |
| **Convenience** | | | |
| Vaccine cost | Vaccine acceptors were more willing to pay for it than unsures and refusers. Acceptors willing to wait more time for a vaccine than unsures. | People felt vaccine should be free, in line with other vaccines provided through government health services. Participants still recognized a low cost would be more cost effective than hospitalization or OTC medications. Convenience not brought up in context of dengue vaccine, but issues of convenience (lines, wait times, access) emerged when talking about COVID-19 experience. | Convergence: Both sources support concept of free vaccine and of reducing wait times, with qualitative results providing more insight about why people expect free vaccine. Discussion in FGDs about access barriers that emerged during COVID-19, despite not bringing this up as an issue for a potential dengue vaccine. |
| **Context** | | | |
| Knowledge of transmission | Not having had the COVID-19 vaccine, as well as lack of knowledge of how dengue is transmitted, was associated with vaccine refusers. | Not addressed, but participant questions focused on whether getting the vaccine could reduce their vector control activities. | Complementary information: Knowledge gaps about dengue transmission contribute to vaccine uncertainty. Education about dengue transmission and control practices should be part of the vaccine campaign to ensure vector control practices continue. |
| Targeted groups | Having a technical or higher education level was association with greater uncertainty about the dengue vaccine. | Key populations, such as religious groups and indigenous communities, were identified as potentially hard to reach groups. There was a strong sentiment of parental motivation to vaccinate children first, even among hesitant adults. | Partial convergence: Both sources found “sub-groups” that might require targeted communication; in surveys, those with higher education and men, whereas in FGDs, they identified indigenous and certain religious groups, and also focused on vaccinating children first. |
| **Communication** | | | |
| Trusted sources | Only significant difference found was that vaccine refusers and “unsures” more likely to want to hear about official approval from Ministry of Health than acceptors, yet less likely to need to know about vaccine side effects than acceptors. | Health professionals, Ministry of Health, and even World Health Organization cited as the most trusted sources of information, despite lack of trust in authority overall expressed in Piura. | Consistent convergence: Health institutions are a source of trusted communication. |
| Need for clear messages | Not asked. | Participants wanted to know the main side effects, who was eligible, how many doses they might need, safety, and “not being the first” to get vaccine. | No information from surveys. Qualitative findings highlight specific examples within unforeseen vaccine effects that concern them. |
| Communication channels | The top communication channels include loudspeakers, social media, and radio broadcasting. | Participants recommended using radio, social media, television, loudspeaker announcements, and community forums to promote vaccine information. | Strong convergence: Need for a multi-channel communication strategy. |
