# Supplementary material for: Dengue vaccine acceptability in Peru: A mixed-methods study in two dengue-endemic Peruvian cities
Source: PLoS Negl Trop Dis. 2026 May 18;20(5):e0013572. doi: 10.1371/journal.pntd.0013572 (PMC13193613; doi:10.1371/journal.pntd.0013572)
Supplement: S1 Text — (DOCX) [file pntd.0013572.s004.docx]

**S1 Material. Variable Selection and Multicollinearity Assessment**

# **Table A. Domain-Specific Variable Assessment Using Binary Logistic Regression (Willing vs Unsure)**

| **N°** | **Domain** | **Subcategory** | **Name of variable** | **Description of variable** | **Category** |  | **Logistic Regression – Crude OR (Willing Vs Unsure)** | | | | **Logistic Regression – Adjusted OR (Willing Vs Unsure)** | | | |
| --- | --- | --- | --- | --- | --- | --- | --- | --- | --- | --- | --- | --- | --- | --- |
|  |  |  |  |  |  |  | **N** | **OR** | **95% CI** | **p-value** | **N** | **OR** | **95% CI** | **p-value** |
| 1 | Confidence | A. Observed evidence and social modeling | conf_vac_cov_pers_salud_vacuna | Observing healthcare workers being vaccinated against COVID-19 increased my confidence in getting vaccinated | Yes |  | 851 | 0.53 | 0.28, 0.94 | 0.028 | 749 | 0.53 | 0.24, 1.10 | 0.09 |
| 2 | Confidence | A. Observed evidence and social modeling | conf_vac_cov_vacunado_no_muere | Seeing that vaccinated people did not die from COVID-19 increased my confidence in vaccination | Yes |  | 851 | 0.65 | 0.44, 0.94 | 0.024 | 749 | 0.75 | 0.45, 1.25 | 0.3 |
| 3 | Confidence | B. Institutional endorsement and legitimacy | conf_vac_cov_oms_minsa_respalda | Knowing that the COVID-19 vaccine was endorsed by WHO or the Ministry of Health increased my confidence in vaccination | Yes |  | 851 | 0.83 | 0.42, 1.52 | 0.6 | 749 | 1.1 | 0.49, 2.35 | 0.8 |
| 4 | Confidence | C. Global attitudes toward vaccination | conf_vac_cov_prot_salud | Knowing that getting vaccinated against COVID-19 would protect my family and my community increased my confidence in vaccination | Yes |  | 851 | 1.11 | 0.60, 1.95 | 0.7 | 749 | 1.15 | 0.53, 2.43 | 0.7 |
| 5 | Confidence | C. Global attitudes toward vaccination | posicion_covid_cat | Overall opinion of the COVID-19 vaccine | Against or in doubt |  | 851 | 3.53 | 2.32, 5.34 | <0.001 | 749 | 2.15 | 1.28, 3.56 | 0.004 |
| 6 | Confidence | C. Global attitudes toward vaccination | av_trust | Trust in vaccine benefits (VAX scale construct) | Intermediate |  | 788 | 0.39 | 0.20, 0.79 | <0.001 | 749 | 0.48 | 0.22, 1.05 | <0.001 |
|  |  |  |  |  | High |  |  | 0.13 | 0.07, 0.25 |  |  | 0.21 | 0.10, 0.45 |  |
| 7 | Confidence | D. Trust in vaccine development and regulation | descon_vac_cov_rapido | Knowing that the COVID-19 vaccine was developed very quickly reduced my confidence in getting vaccinated | Yes |  | 851 | 2.34 | 1.41, 3.80 | 0.001 | 749 | 2.24 | 1.25, 3.93 | 0.007 |
| 8 | Confidence | D. Trust in vaccine development and regulation | av_worries | Concerns about unforeseen future effects of vaccines (VAX scale construct) | Intermediate |  | 846 | 3.25 | 1.40, 9.49 | 0.009 | 749 | 3.22 | 1.30, 9.86 | 0.035 |
|  |  |  |  |  | High |  |  | 3.51 | 1.49, 10.3 |  |  | 2.74 | 1.07, 8.52 |  |
| 9 | Confidence | D. Trust in vaccine development and regulation | av_commercial | Concerns about commercial profiteering related to vaccines (VAX scale construct) | Intermediate |  | 841 | 2.03 | 1.31, 3.23 | 0.006 | 749 | 1.29 | 0.78, 2.15 | 0.6 |
|  |  |  |  |  | High |  |  | 1.41 | 0.79, 2.50 |  |  | 1.04 | 0.53, 2.01 |  |
| 10 | Confidence | E. Policy environment and coercion-related perceptions | descon_vac_cov_obligatorio | Knowing that COVID-19 vaccination was mandatory reduced my confidence in getting vaccinated | Yes |  | 851 | 1.47 | 0.88, 2.37 | 0.13 | 749 | 1.16 | 0.64, 2.03 | 0.6 |
| 11 | Confidence | F. Interpersonal trust in health information | people_trust | Presence of a trusted healthcare or community figure for health-related information | Yes |  | 817 | 0.62 | 0.37, 1.01 | 0.054 | 749 | 0.69 | 0.39, 1.18 | 0.2 |
| 12 | Complacency | A. Perceived susceptibility to dengue | p_rd_assume_cat1 | Participant believes they could become ill with dengue | Agree |  | 850 | 0.61 | 0.42, 0.89 | 0.011 | 836 | 0.96 | 0.60, 1.55 | 0.9 |
| 13 | Complacency | A. Perceived susceptibility to dengue | p_rd_chance_cat1 | Participant perceives a high probability of contracting dengue | Agree |  | 850 | 0.6 | 0.40, 0.89 | 0.01 | 836 | 0.85 | 0.52, 1.39 | 0.5 |
| 14 | Complacency | A. Perceived susceptibility to dengue | p_rd_risk_dengue_cat1 | Participant perceives everyone is at risk of contracting dengue | Agree |  | 850 | 0.42 | 0.27, 0.66 | <0.001 | 836 | 0.65 | 0.38, 1.12 | 0.12 |
| 15 | Complacency | A. Perceived susceptibility to dengue | p_rd_dengue_again_cat1 | Participant believes they could get dengue again if previously infected | Agree |  | 850 | 0.46 | 0.31, 0.67 | <0.001 | 836 | 0.64 | 0.39, 1.04 | 0.072 |
| 16 | Complacency | B. Perceived severity and consequences | p_sd_serius_cat1 | Participant perceives dengue as a serious or severe disease | Agree |  | 850 | 0.45 | 0.21, 1.06 | 0.065 | 836 | 1.26 | 0.47, 3.66 | 0.7 |
| 17 | Complacency | B. Perceived severity and consequences | p_sd_died_cat1 | Participant believes people can die from dengue infection | Agree |  | 850 | 0.3 | 0.16, 0.60 | <0.001 | 836 | 0.52 | 0.22, 1.23 | 0.13 |
| 18 | Complacency | B. Perceived severity and consequences | p_sd_limit_cat1 | Participant perceives dengue causes limitations in daily activities | Agree |  | 850 | 0.48 | 0.32, 0.70 | <0.001 | 836 | 0.68 | 0.43, 1.07 | 0.095 |
| 19 | Complacency | B. Perceived severity and consequences | p_sd_loss_cat1 | Participant perceives dengue may lead to income loss | Agree |  | 849 | 0.51 | 0.32, 0.83 | 0.008 | 836 | 0.83 | 0.45, 1.53 | 0.5 |
| 20 | Complacency | B. Perceived severity and consequences | p_sd_money_cat1 | Participant perceives dengue entails high out-of-pocket costs | Agree |  | 848 | 0.6 | 0.40, 0.91 | 0.018 | 836 | 0.84 | 0.51, 1.42 | 0.5 |
| 21 | Complacency | C. Preference for natural immunity | av_natural | Preference for natural immunity (VAX scale construct) | Intermediate |  | 839 | 1.91 | 1.24, 2.97 | 0.002 | 836 | 1.95 | 1.24, 3.07 | 0.009 |
|  |  |  |  |  | High |  |  | 0.91 | 0.53, 1.52 |  |  | 1.13 | 0.65, 1.94 |  |
| 22 | Convenience | A. Prior logistical barriers (COVID-19) | vac_covid_dificultad_tiempo | Experienced time-related difficulties during COVID-19 vaccination | Yes |  | 833 | 1.22 | 0.57, 2.38 | 0.6 | 832 | 1.26 | 0.45, 3.45 | 0.7 |
| 23 | Convenience | A. Prior logistical barriers (COVID-19) | vac_covid_dificultad_cola | Experienced long waiting lines during COVID-19 vaccination | Yes |  | 833 | 1.05 | 0.55, 1.86 | 0.9 | 832 | 1.04 | 0.42, 2.37 | >0.9 |
| 24 | Convenience | B. Affordability and financial accessibility | cost_vacc_dengue_si | Willingness to pay for a dengue vaccine | Yes |  | 851 | 0.32 | 0.20, 0.50 | <0.001 | 832 | 0.33 | 0.20, 0.52 | <0.001 |
| 25 | Convenience | C. Accessibility of delivery strategies | estra_vac_casa | Vaccination delivered door-to-door perceived as a good strategy | Yes |  | 851 | 0.72 | 0.49, 1.07 | 0.1 | 832 | 0.53 | 0.33, 0.85 | 0.009 |
| 26 | Convenience | C. Accessibility of delivery strategies | estra_vac_colegio | Vaccination delivered at schools perceived as a good strategy | Yes |  | 851 | 0.95 | 0.58, 1.50 | 0.8 | 832 | 0.72 | 0.41, 1.23 | 0.2 |
| 27 | Convenience | C. Accessibility of delivery strategies | estra_lugares_publicos | Vaccination delivered in public spaces perceived as a good strategy | Yes |  | 851 | 1.84 | 1.17, 2.83 | 0.009 | 832 | 1.52 | 0.89, 2.53 | 0.12 |
| 28 | Convenience | C. Accessibility of delivery strategies | estra_vac_postas_hospital | Vaccination delivered at health facilities perceived as a good strategy | Yes |  | 851 | 0.59 | 0.34, 0.99 | 0.044 | 832 | 0.57 | 0.30, 1.02 | 0.058 |
| 29 | Convenience | D. Temporal accessibility and tolerance | time_vaccine_cat1 | Willingness to spend travel time to reach dengue vaccination site | One hour or more |  | 850 | 1.14 | 0.69, 1.84 | 0.6 | 832 | 1.48 | 0.84, 2.54 | 0.2 |
| 30 | Convenience | D. Temporal accessibility and tolerance | tiempo_espera_cat1 | Willingness to wait in line at dengue vaccination site | One hour or more |  | 850 | 0.58 | 0.40, 0.85 | 0.005 | 832 | 0.55 | 0.36, 0.83 | 0.004 |
| 31 | Communication | A. Misinformation exposure | descon_vac_cov_fake_news | Negative information or misinformation encountered through news media or social networks was a major factor contributing to my distrust of COVID-19 vaccination. | Yes |  | 851 | 1.43 | 0.98, 2.09 | 0.061 | 838 | 1.44 | 0.97, 2.15 | 0.072 |
| 32 | Communication | B. Information needs prior to decision | vac_deng_infor_dosis | Desire to know the number of doses before dengue vaccination | Yes |  | 851 | 0.76 | 0.50, 1.14 | 0.2 | 838 | 0.74 | 0.47, 1.15 | 0.2 |
| 33 | Communication | B. Information needs prior to decision | vac_deng_infor_minsa_oms | Before deciding whether to receive a dengue vaccine, I would like to know whether it has been officially approved by Ministry of Health or WHO | Yes |  | 851 | 2.36 | 1.27, 4.21 | 0.007 | 838 | 2.53 | 1.29, 4.85 | 0.008 |
| 34 | Communication | B. Information needs prior to decision | vac_deng_infor_reacciones | Before getting vaccinated against dengue, I would like to know about the vaccine’s adverse effects | Yes |  | 851 | 0.61 | 0.39, 0.99 | 0.047 | 838 | 0.66 | 0.40, 1.09 | 0.1 |
| 35 | Communication | B. Information needs prior to decision | vac_deng_infor_efectivo | Before getting vaccinated against dengue, I would like to know how effective the vaccine is | Yes |  | 851 | 1.15 | 0.76, 1.77 | 0.5 | 838 | 1.21 | 0.77, 1.94 | 0.4 |
| 36 | Communication | B. Information needs prior to decision | vac_deng_infor_laboratorio | Before deciding whether to receive a dengue vaccine, I would like to know which pharmaceutical laboratory or manufacturer developed the vaccine | Yes |  | 851 | 1.12 | 0.56, 2.08 | 0.7 | 838 | 0.89 | 0.42, 1.77 | 0.8 |
| 37 | Communication | C. Preferred institutional sources | inst_vac_minsa | Preference for Ministry of Health as source of dengue vaccine information | Yes |  | 851 | 0.7 | 0.48, 1.03 | 0.072 | 838 | 0.47 | 0.30, 0.73 | <0.001 |
| 38 | Communication | C. Preferred institutional sources | inst_vac_centro_salud | Health centers or local primary care facilities as source of dengue vaccine information | Yes |  | 851 | 0.5 | 0.32, 0.74 | <0.001 | 838 | 0.37 | 0.23, 0.58 | <0.001 |
| 39 | Communication | D. Preferred communication channels | com_vacuna | Media to publicize the vaccine (Ref. Radio) | Tv |  | 838 | 0.89 | 0.42, 1.84 | 0.7 | 838 | 1.03 | 0.47, 2.19 | 0.5 |
|  |  |  |  |  | Social media |  |  | 1.29 | 0.72, 2.38 |  | 838 | 1.49 | 0.81, 2.82 |  |
|  |  |  |  |  | Perifoneo |  |  | 1.3 | 0.74, 2.37 |  |  | 1.43 | 0.79, 2.69 |  |
|  |  |  |  |  | House-to-house notification |  |  | 1.01 | 0.44, 2.20 |  |  | 0.95 | 0.40, 2.17 |  |
| 40 | Context | A. Geographic and programmatic context | site | Study site (Ref. Iquitos) | Piura |  | 851 | 0.84 | 0.58, 1.22 | 0.4 | 812 | 1.39 | 0.82, 2.39 | 0.2 |
| 41 | Context | A. Geographic and programmatic context | zona_intervencion | Residence in an area with prior public health intervention | Yes |  | 851 | 0.76 | 0.52, 1.11 | 0.2 | 812 | 0.93 | 0.60, 1.43 | 0.7 |
| 42 | Context | B. Basic sociodemographic characteristics | edad_cat_2 | Age group of participant (Ref. 18 to 39 years old) | 40-60 yrs |  | 851 | 0.63 | 0.43, 0.93 | 0.018 | 812 | 0.81 | 0.51, 1.28 | 0.4 |
| 43 | Context | B. Basic sociodemographic characteristics | tpca2 | Wealth index terciles (Ref. Level 1- poorer) | Level 2 |  | 839 | 0.78 | 0.47, 1.29 | 0.046 | 812 | 0.66 | 0.36, 1.19 | 0.052 |
|  |  |  |  |  | Level 3 (Wealthier) |  |  | 1.41 | 0.90, 2.21 |  |  | 1.32 | 0.70, 2.50 |  |
| 44 | Context | B. Basic sociodemographic characteristics | sexo | Sex of participant (Ref. Female) | Male |  | 851 | 2.11 | 1.43, 3.11 | <0.001 | 812 | 1.76 | 1.02, 3.07 | 0.041 |
| 45 | Context | B. Basic sociodemographic characteristics | nivel_educativo_cat_1 | Level education (Ref. Elementary school) | High school |  | 851 | 1.47 | 0.81, 2.84 | 0.019 | 812 | 1.59 | 0.77, 3.49 | 0.12 |
|  |  |  |  |  | Technical |  |  | 2.19 | 1.21, 4.20 |  |  | 2.45 | 1.03, 6.15 |  |
| 46 | Context | B. Basic sociodemographic characteristics | estado_civil_cat_1 | Marital status (Ref. Single) | Married |  | 851 | 0.74 | 0.45, 1.19 | 0.086 | 812 | 0.88 | 0.50, 1.53 | 0.7 |
|  |  |  |  |  | Cohabitant |  |  | 0.62 | 0.40, 0.96 |  |  | 0.83 | 0.49, 1.37 |  |
| 47 | Context | B. Basic sociodemographic characteristics | ocupacion_cat1 | Occupation (Ref. Homemaker) | Qualified worker |  | 845 | 2.05 | 1.32, 3.23 | 0.002 | 812 | 1.06 | 0.53, 2.12 | 0.5 |
|  |  |  |  |  | No Qualified worker |  |  | 1.98 | 1.20, 3.25 |  |  | 1.39 | 0.73, 2.62 |  |
| 48 | Context | C. Prior disease and vaccination experiences | exposure_covid_cerc_grave | Close experience with severe COVID-19 | Yes |  | 851 | 0.66 | 0.43, 1.00 | 0.048 | 812 | 0.53 | 0.32, 0.85 | 0.009 |
| 49 | Context | C. Prior disease and vaccination experiences | exposure_dengue_cerc_grave | Close experience with severe dengue | Yes |  | 851 | 0.9 | 0.51, 1.51 | 0.7 | 812 | 1.16 | 0.62, 2.08 | 0.6 |
| 50 | Context | C. Prior disease and vaccination experiences | vac_covid_dosis | Number of COVID-19 vaccine doses received (Ref. Third or fourth doses) | No or incomplete doses |  | 851 | 2.5 | 1.59, 3.85 | <0.001 | 812 | 2.51 | 1.51, 4.13 | <0.001 |
| 51 | Context | D. Cultural, religious, and political values | religion_cat | Importance of religion in participant’s life (Ref. No important) | Some important |  | 850 | 1.19 | 0.48, 3.18 | 0.5 | 812 | 1.52 | 0.55, 4.56 | 0.7 |
|  |  |  |  |  | Is very important |  |  | 0.85 | 0.40, 1.99 |  |  | 1.38 | 0.58, 3.74 |  |
| 52 | Context | D. Cultural, religious, and political values | politica_cat | Self-identified political orientation (Left) | Center |  | 850 | 1.1 | 0.51, 2.74 | >0.9 | 812 | 1.23 | 0.52, 3.29 | 0.6 |
|  |  |  |  |  | Right |  |  | 1.01 | 0.35, 3.02 |  |  | 0.85 | 0.25, 2.89 |  |
| 53 | Context | E. Health literacy and dengue knowledge | dengue_transmision | Know how dengue is transmitted | Yes |  | 849 | 0.57 | 0.34, 0.97 | 0.039 | 812 | 0.48 | 0.25, 0.93 | 0.031 |
| 54 | Context | E. Health literacy and dengue knowledge | conoce_aedes | Knowledge of the dengue-transmitting mosquito | Yes |  | 851 | 1 | 0.67, 1.47 | >0.9 | 812 | 1.1 | 0.69, 1.75 | 0.7 |
| 55 | Context | E. Health literacy and dengue knowledge | correct_symptoms_dengue | Know at least 4 symptoms of dengue | Yes |  | 851 | 0.85 | 0.58, 1.24 | 0.4 | 812 | 0.98 | 0.63, 1.53 | >0.9 |
| 56 | Context | F. Prior dengue control intervention experiences | fumigacion_outcome_1 | Levels of Fumigation Hesitancy (Ref. Not Hesitant) | Occasionally Hesitant |  | 835 | 1.52 | 0.89, 2.50 | 0.038 | 812 | 1.1 | 0.57, 2.06 | 0.3 |
|  |  |  |  |  | Hesitant |  |  | 2.29 | 1.10, 4.47 |  |  | 2 | 0.85, 4.44 |  |
| 57 | Context | F. Prior dengue control intervention experiences | calidad_fumigacion | Perception of Effectiveness of Fumigation Product (Ref. Poor Effectiveness) | Indifferent to Effectiveness |  | 849 | 0.94 | 0.55, 1.54 | 0.042 | 812 | 0.98 | 0.53, 1.74 | 0.2 |
|  |  |  |  |  | Good Effectiveness |  |  | 0.52 | 0.29, 0.87 |  |  | 0.57 | 0.30, 1.03 |  |
| 58 | Context | F. Prior dengue control intervention experiences | abatizacion_outcome_1 | Levels of Focal Treatment Hesitancy (Ref. Not Hesitant) | Occasionally Hesitant |  | 850 | 1.91 | 0.79, 4.16 | 0.13 | 812 | 1.04 | 0.34, 2.82 | >0.9 |
|  |  |  |  |  | Hesitant |  |  | 2.11 | 0.75, 5.19 |  |  | 0.92 | 0.22, 3.04 |  |
| 59 | Context | F. Prior dengue control intervention experiences | calidad_tto_focal | Perception of Effectiveness of Focal Treatment Product | Indifferent to Effectiveness |  | 847 | 0.81 | 0.41, 1.65 | 0.052 | 812 | 0.72 | 0.32, 1.67 | 0.2 |
|  |  |  |  |  | Good Effectiveness |  |  | 0.53 | 0.29, 1.01 |  |  | 0.54 | 0.26, 1.15 |  |

# **Section B. Assessment of Multicollinearity Within Each 5C Domain**

## **Table B. Confidence – Variance Inflation Factors (VIF)**

| **Predictor** | **Adjusted VIF*** |
| --- | --- |
| conf_vac_cov_pers_salud_vacuna | 1.17 |
| conf_vac_cov_vacunado_no_muere | 1.22 |
| conf_vac_cov_oms_minsa_respalda | 1.11 |
| conf_vac_cov_prot_salud | 1.15 |
| posicion_covid_cat | 1.11 |
| av_trust | 1.05 |
| descon_vac_cov_rapido | 1.02 |
| av_worries | 1.03 |
| av_commercial | 1.04 |
| descon_vac_cov_obligatorio | 1.02 |
| people_trust | 1.01 |

*Adjusted VIF corresponds to GVIF^(1/(2·Df)) derived from a domain-specific multivariable logistic regression model.

## **Table C. Complacency – Variance Inflation Factors (VIF)**

| **Predictor** | **Adjusted VIF*** |
| --- | --- |
| p_rd_assume_cat1 | 1.21 |
| p_rd_chance_cat1 | 1.19 |
| p_rd_risk_dengue_cat1 | 1.14 |
| p_rd_dengue_again_cat1 | 1.24 |
| p_sd_serius_cat1 | 1.22 |
| p_sd_died_cat1 | 1.23 |
| p_sd_limit_cat1 | 1.14 |
| p_sd_loss_cat1 | 1.22 |
| p_sd_money_cat1 | 1.19 |
| av_natural | 1.01 |

*Adjusted VIF corresponds to GVIF^(1/(2·Df)) derived from a domain-specific multivariable logistic regression model.

## **Table D. Convenience – Variance Inflation Factors (VIF)**

| **Predictor** | **VIF*** |
| --- | --- |
| vac_covid_dificultad_tiempo | 1.95 |
| vac_covid_dificultad_cola | 1.91 |
| cost_vacc_dengue_si | 1.04 |
| estra_vac_casa | 1.34 |
| estra_vac_colegio | 1.18 |
| estra_lugares_publicos | 1.24 |
| estra_vac_postas_hospital | 1.22 |
| time_vaccine_cat1 | 1.08 |
| tiempo_espera_cat1 | 1.08 |

* Variance inflation factors were estimated from a domain-specific multivariable logistic regression model. All variables had dichotomic categories.

**Table E. Communication – Variance Inflation Factors (VIF)**

| **Predictor** | **Adjusted VIF*** |
| --- | --- |
| descon_vac_cov_fake_news | 1.02 |
| vac_deng_infor_dosis | 1.05 |
| vac_deng_infor_minsa_oms | 1.08 |
| vac_deng_infor_reacciones | 1.01 |
| vac_deng_infor_efectivo | 1.04 |
| vac_deng_infor_laboratorio | 1.07 |
| inst_vac_minsa | 1.12 |
| inst_vac_centro_salud | 1.12 |
| com_vacuna | 1.02 |

*Adjusted VIF corresponds to GVIF^(1/(2·Df)) derived from a domain-specific multivariable logistic regression model.

## **Table F. Context – Variance Inflation Factors (VIF)**

| **Predictor** | **Adjusted VIF*** |
| --- | --- |
| site | 1.30 |
| zona_intervencion | 1.04 |
| edad_cat_2 | 1.10 |
| tpca2 | 1.17 |
| sexo | 1.30 |
| nivel_educativo_cat_1 | 1.22 |
| estado_civil_cat_1 | 1.06 |
| ocupacion_cat1 | 1.26 |
| exposure_covid_cerc_grave | 1.06 |
| exposure_dengue_cerc_grave | 1.05 |
| vac_covid_dosis | 1.06 |
| religion_cat | 1.06 |
| politica_cat | 1.04 |
| dengue_transmision | 1.09 |
| conoce_aedes | 1.09 |
| correct_symptoms_dengue | 1.07 |
| fumigacion_outcome_1 | 1.11 |
| calidad_fumigacion | 1.05 |
| abatizacion_outcome_1 | 1.07 |
| calidad_tto_focal | 1.05 |

*Adjusted VIF corresponds to GVIF^(1/(2·Df)) derived from a domain-specific multivariable logistic regression model.

# **Section C. Variables Retained in the Final Multivariable Model**

## **Table G. Final set of selected predictors across domains**

| **N°** | **Domain** | **Subcategory** | **Name of variable** | **Description of variable** |  |
| --- | --- | --- | --- | --- | --- |
|  |  |  |  |  |  |
| 1 | Confidence | C. Global attitudes toward vaccination | posicion_covid_cat | Overall opinion of the COVID-19 vaccine |  |
| 2 | Confidence | C. Global attitudes toward vaccination | av_trust | Trust in vaccine benefits (VAX scale construct) |  |
| 3 | Confidence | D. Trust in vaccine development and regulation | descon_vac_cov_rapido | Knowing that the COVID-19 vaccine was developed very quickly reduced my confidence in getting vaccinated |  |
| 4 | Confidence | D. Trust in vaccine development and regulation | av_worries | Concerns about unforeseen future effects of vaccines (VAX scale construct) |  |
| 5 | Complacency | A. Perceived susceptibility to dengue | p_rd_dengue_again_cat1 | Participant believes they could get dengue again if previously infected |  |
| 6 | Complacency | B. Perceived severity and consequences | p_sd_died_cat1 | Participant believes people can die from dengue infection |  |
| 7 | Complacency | B. Perceived severity and consequences | p_sd_loss_cat1 | Participant perceives dengue may lead to income loss |  |
| 8 | Complacency | C. Preference for natural immunity | av_natural | Preference for natural immunity (VAX scale construct) |  |
| 9 | Convenience | B. Affordability and financial accessibility | cost_vacc_dengue_si | Willingness to pay for a dengue vaccine |  |
| 10 | Convenience | D. Temporal accessibility and tolerance | tiempo_espera_cat1 | Willingness to wait in line at dengue vaccination site |  |
| 11 | Communication | A. Misinformation exposure | descon_vac_cov_fake_news | Negative information or misinformation encountered through news media or social networks was a major factor contributing to my distrust of COVID-19 vaccination. |  |
| 12 | Communication | B. Information needs prior to decision | vac_deng_infor_minsa_oms | Before deciding whether to receive a dengue vaccine, I would like to know whether it has been officially approved by Ministry of Health or WHO |  |
| 13 | Communication | C. Preferred institutional sources | inst_vac_minsa | Preference for Ministry of Health as source of dengue vaccine information |  |
| 14 | Communication | C. Preferred institutional sources | inst_vac_centro_salud | Health centers or local primary care facilities as source of dengue vaccine information |  |
| 15 | Communication | D. Preferred communication channels | com_vacuna | Media to publicize the vaccine (Ref. Radio) |  |
| 16 | Context | A. Geographic and programmatic context | site | Study site (Ref. Iquitos) |  |
| 17 | Context | A. Geographic and programmatic context | zona_intervencion | Residence in an area with prior public health intervention |  |
| 18 | Context | C. Prior disease and vaccination experiences | exposure_covid_cerc_grave | Close experience with severe COVID-19 |  |
| 19 | Context | C. Prior disease and vaccination experiences | exposure_dengue_cerc_grave | Close experience with severe dengue |  |
| 20 | Context | C. Prior disease and vaccination experiences | vac_covid_dosis | Number of COVID-19 vaccine doses received (Ref. Third or fourth doses) |  |
| 21 | Context | E. Health literacy and dengue knowledge | dengue_transmision | Know how dengue is transmitted |  |

## **Table H. A priori sociodemographic covariates included for adjustment**

| **N°** | **Domain** | **Subcategory** | **Name of variable** | **Description of variable** |  |
| --- | --- | --- | --- | --- | --- |
|  |  |  |  |  |  |
| 1 | Sociodemographic covariates | Sex | sexo | Sex of participant (Ref. Female) |  |
| 2 | Sociodemographic covariates | Age | edad_cat_2 | Age group of participant (Ref. 18 to 39 years old) |  |
| 3 | Sociodemographic covariates | Education | nivel_educativo_cat_1 | Level education (Ref. Elementary school) |  |
| 4 | Sociodemographic covariates | Occupation | ocupacion_cat1 | Occupation (Ref. Homemaker) |  |

# **Section D. Multicollinearity Assessment of the Final Multivariable Model**

## **Table I. Variance Inflation Factors (VIF) for predictors included in the final model**

| **Predictor** | **Adjusted VIF*** |
| --- | --- |
| posicion_covid_cat | 1.15 |
| av_trust | 1.08 |
| descon_vac_cov_rapido | 1.05 |
| av_worries | 1.06 |
| p_rd_dengue_again_cat1 | 1.09 |
| p_sd_died_cat1 | 1.11 |
| p_sd_loss_cat1 | 1.08 |
| av_natural | 1.06 |
| cost_vacc_dengue_si | 1.05 |
| tiempo_espera_cat1 | 1.12 |
| descon_vac_cov_fake_news | 1.07 |
| vac_deng_infor_minsa_oms | 1.09 |
| inst_vac_minsa | 1.19 |
| inst_vac_centro_salud | 1.27 |
| com_vacuna | 1.11 |
| site | 1.52 |
| zona_intervencion | 1.09 |
| exposure_covid_cerc_grave | 1.09 |
| exposure_dengue_cerc_grave | 1.08 |
| vac_covid_dosis | 1.09 |
| dengue_transmision | 1.13 |
| sexo | 1.25 |
| edad_cat_2 | 1.07 |
| nivel_educativo_cat_1 | 1.21 |
| ocupacion_cat1 | 1.25 |

*Adjusted VIF corresponds to GVIF^(1/(2·Df)) derived from the final multivariable logistic regression model.
