# Supplementary material for: Dengue vaccine acceptability in Peru: A mixed-methods study in two dengue-endemic Peruvian cities
Source: PLoS Negl Trop Dis. 2026 May 18;20(5):e0013572. doi: 10.1371/journal.pntd.0013572 (PMC13193613; doi:10.1371/journal.pntd.0013572)
Supplement: S2 Text — (DOCX) [file pntd.0013572.s005.docx]

**S2 Text:**

**Description and adaptation of Oxford COVID-19 Vaccine Hesitancy Scale**

## **1. Origin and conceptual framework for the instrument**

At the time of the design and implementation of this study, there was no validated instrument specifically developed to measure vaccine hesitancy toward a dengue vaccination. Although vaccine hesitancy has been extensively studied in the context of routine childhood immunizations and, more recently, COVID-19 vaccines, no standardized scale had been adapted or validated for dengue, particularly in settings where a dengue vaccine was not yet widely available or implemented.

To address this gap, we adapted the Oxford COVID-19 Vaccine Hesitancy Scale, developed by Freeman et al. [1] as part of the *Oxford Coronavirus Explanations, Attitudes, and Narratives Survey (OCEANS II)*. This instrument was originally designed to capture gradients of vaccine acceptance, uncertainty, and refusal in the UK during the pre-implementation phase of COVID-19 vaccination. The Oxford scale conceptualizes vaccine hesitancy as a multidimensional construct, reflected through a set of related but distinct attitudinal and behavioral indicators, rather than as a binary decision.

The decision to adapt this instrument to the dengue context was guided by strong conceptual and contextual parallels between the two vaccination scenarios. In both cases, individuals are asked to evaluate a novel potential or recently introduced vaccine under conditions characterized by incomplete or evolving information, uncertainty regarding safety and effectiveness, widespread circulation of risk-related narratives through formal and informal channels, and the need to make vaccination decisions in advance of large-scale implementation. These shared features make the Oxford COVID-19 Vaccine Hesitancy Scale a theoretically appropriate framework for assessing dengue vaccine hesitancy in a pre-implementation setting.

Importantly, the adaptation of the Oxford scale for dengue vaccination was intended to leverage an established conceptual framework, rather than to formally re-validate the instrument psychometrically. The primary objective was to capture meaningful variation in attitudes, intentions, and anticipated behaviors related to dengue vaccination, allowing for the identification of gradients of acceptance, uncertainty, and hesitancy within the study population. As such, this adaptation should be understood as a contextual application of an existing measurement approach, rather than the development of a newly validated dengue-specific hesitancy scale. The specific adaptations made to the individual items, including modifications introduced during pilot testing, are described in detail in the following subsections.

## **2. Description of the original Oxford hesitancy items**

The original Oxford COVID-19 Vaccine Hesitancy Scale, developed by Freeman et al. within the OCEANS II study [1], consists of seven ordinal items designed to capture different dimensions of vaccine-related attitudes, intentions, and anticipated behaviors (Table A in S2 Text). Each item was measured using an ordinal response scale with up to six response categories, allowing respondents to express varying degrees of acceptance, uncertainty, or resistance toward vaccination. In this framework, responses coded as 1 or 2 reflect clear acceptance or positive orientation toward vaccination, category 3 represents a neutral or undecided position, and categories 4 and 5 indicate explicit hesitancy or resistance to vaccination (Table A in S2 Text). The final category, “Don’t know” (category 6), captures respondents who are unable or unwilling to express a directional attitude (Table A in S2 Text).

In the original Oxford study, “Don’t know” responses were not treated as equivalent to either acceptance or hesitancy. Instead, they were handled analytically as a distinct form of uncertainty, either excluded from certain summary measures or treated as missing information at the item level, depending on the analytical approach. This distinction reflects the authors’ conceptualization of vaccine hesitancy as a spectrum ranging from strong acceptance to strong refusal, with uncertainty occupying a separate position that does not necessarily imply opposition to vaccination.

The seven items were analyzed jointly to construct both categorical classifications of vaccine hesitancy (e.g., willing, very unsure, strongly hesitant) and continuous representations of hesitancy, including summed scores and latent factor scores derived from confirmatory factor analysis. This multi-item, multi-approach strategy allowed the original authors to capture nuanced patterns of vaccine-related decision-making beyond reliance on a single question.

## **3. Adaptation of the key question of general acceptance and anticipated action if a dengue vaccine became available (Item 1), which represents the instruments only dichotomous response variable.**

For the purposes of the present study, the seven Oxford hesitancy items were adapted linguistically and contextually to refer specifically to dengue vaccination, while preserving their original conceptual intent. All items were framed to assess anticipated attitudes and behaviors in a pre-implementation context, reflecting the fact that a dengue vaccine was not yet routinely available to the study population at the time of data collection.

During pilot testing of the adapted questionnaire, we identified conceptual redundancy between two items: (i) Item 1, which assessed general acceptance of a dengue vaccine if it were available, and, (ii) Item 4, which assessed the respondent’s anticipated action if a dengue vaccine were available to them. Both items addressed closely related constructs—general acceptance versus concrete behavioral intention—using highly similar response structures. To reduce redundancy and respondent burden, and to improve clarity during field implementation, a methodological decision was made to simplify the response options of Item 1.

Specifically, Item 1 was modified to use a simplified trichotomous response format: Yes / No / Don’t know, instead of the original six-category ordinal scale. This modification was applied only to Item 1; the remaining six items retained their original ordinal structure. As a result, the modification altered the original ordinal structure of the first item and limited its direct comparability with the remaining items. This change has important analytical implications, which are addressed explicitly in the modeling strategies described in subsequent sections.

For analytical consistency with the conceptual framework of the Oxford scale, responses to the modified Item 1 were recoded as follows:

- Yes → equivalent to a pro-vaccine response (coded 2)
- No → equivalent to a hesitant response (coded 5)
- Don’t know → treated as an uncertainty response (“Don't know / DK”) and coded as category 6

By explicitly documenting this modification and its analytical handling, we aim to ensure transparency in the construction of the dengue vaccine hesitancy outcomes and to clarify how potential inconsistencies introduced during questionnaire adaptation were addressed in the analysis.

Table A in S2 Text. Adaptation of the Oxford Vaccine Hesitancy Scale to the dengue context and modification of Item1

| **Item** | **Original Oxford COVID-19 Vaccine Hesitancy Scale** | | **Adapted dengue vaccine hesitancy scale** | |
| --- | --- | --- | --- | --- |
|  | **Original question (English)** | **Response options (English)** | **English (Spanish translation)** | **Response options in English (Spanish)** |
| 1 | Would you take a COVID-19 vaccine (approved for use in the UK) if offered? | 1, Definitely 2, Probably 3, I may or I may not 4, Probably not 5, Definitely not 6, Don't know | If a vaccine against dengue fever were available, would you accept the vaccine if it were offered to you?  (¿Si hubiera una vacuna disponible contra dengue aceptaría la vacuna si se le ofreciera?) | 1, No (No) 2, Yes (Sí)  3, Don’t Know (No sé) |
| 2 | If there is a COVID-19 vaccine available | 1, I will want to get it as soon as possible 2, I will take it when offered 3, I’m not sure what I will do 4, I will put off (delay) getting it 5, I will refuse to get it 6, Don't know | If there is a dengue vaccine available (Consider the following)  (Si en la actualidad existiera una vacuna contra dengue. Usted consideraría lo siguiente:) | 1, I will want to get it as soon as possible (Me gustaría aplicármela lo antes posible)  2, I will take it when offered (Me aplicaría cuando me la ofrezcan ) 3, I’m not sure what I will do (No estoy seguro de lo que haré) 4, I will put off (delay) getting it (Pospondré (retrasaré) su aplicación)  5, I will refuse to get it (Me negaré a aplicármela) 6, Don’t know (No sé) |
| 3 | I would describe my attitude towards receiving a COVID-19 vaccine as: | 1, Very keen 2, Pretty positive 3, Neutral  4, Quite uneasy 5, Against it 6, Don't know | I would describe my attitude towards receiving a dengue vaccine as:  (Describiría mi actitud hacia recibir una vacuna contra dengue cómo:) | 1, Very keen (Muy entusiasta) 2, Pretty positive (Bastante positivo) 3, Neutral (Neutral) 4, Quite uneasy (Bastante preocupado(a)) 5, Against it (En contra de la vacuna) 6, Don’t know (No sé) |
| 4 | If a COVID-19 vaccine was available at my local pharmacy, I would: | 1, Get it as soon as possible 2, Get it when I have time 3, Delay getting it  4, Avoid getting it for as long as possible 5, Never get it 6, Don't know | If a dengue vaccine was available to you, what would you do?  (¿Si ya estuviera disponible para usted una vacuna contra DENGUE, que haría?) | 1, I would get it as soon as possible (Me la aplicaría tan pronto como pueda)  2, I would get it when I have time (Me la aplicaría cuando tenga tiempo)  3, I would delay getting it (Retrasaría su aplicación)  4, I would avoid getting it for as long as possible (Evitaría aplicármela durante el mayor tiempo posible)  5, I would never get it (Nunca me la aplicaría)  6, Don’t know (No sé) |
| 5 | If my family or friends were thinking of getting a COVID-19 vaccination, I would: | 1, Strongly encourage them 2, Encourage them 3, Not say anything to them about it 4, Ask them to delay getting the vaccination 5, Suggest that they do not get the vaccination 6, Don't know | If my family or friends were thinking of getting a dengue vaccination, I would do the following:  (Si mi familia o amigos estuvieran pensando en vacunarse contra el dengue, yo haría lo siguiente:) | 1, Strongly encourage them (Los animaría con entusiasmo)  2, Encourage them (Los animaría)  3, Not say anything to them about it (No les diría nada al respecto)  4, Ask them to delay getting the vaccination (Les pediría que retrasen su vacuna) 5, Suggest that they do not get the vaccination (Les sugerirIa que No sé vacunen)  6, Don’t know (No sé) |
| 6 | I would describe myself as: | 1, Willing to get the COVID-19 vaccine  2, Not bothered about getting the COVID-19 vaccine 3, Unwilling to get the COVID-19 vaccine 4, Unwilling to get the COVID-19 vaccine 5, Anti-vaccination for COVID-19 6, Don't know | Regarding receiving the dengue vaccine, I would describe myself as follows:  (Con respecto a recibir la vacuna contra dengue, yo me Describiría cómo:) | 1, Eager to receive the dengue vaccine (Ansioso porque quiero recibir la vacuna contra dengue) 2, Willing to get the dengue vaccine (Dispuesto a recibir la vacuna contra dengue) 3, Not bothered about getting the dengue vaccine (No preocupado por recibir la vacuna contra dengue)  4, Unwilling to get the dengue vaccine (No dispuesto a recibir la vacuna contra dengue)  5, Against the dengue vaccine (En contra de la vacuna contra dengue)  6, Don’t know (No sé) |
| 7 | Taking a COVID-19 vaccination is: | 1, Really important  2, Important 3, Neither important nor unimportant 4, Unimportant 5, Really unimportant 6, Don't know | For me, receiving a dengue vaccine is:  (Considero que recibir una vacuna contra DENGUE para mí es:) | 1, Really important (Realmente importante)  2, Important (Importante)  3, Neither important nor unimportant (Ni importante ni no importante) 4, Unimportant (No es importante)  5, Really unimportant (Realmente no es importante)  6, Don’t know (No sé) |

**References.**

1. Freeman D, Loe BS, Chadwick A, Vaccari C, Waite F, Rosebrock L, et al. COVID-19 vaccine hesitancy in the UK: the Oxford coronavirus explanations, attitudes, and narratives survey (Oceans) II. Psychol Med. 2022 Oct 11;52(14):3127–41. doi:10.1017/S0033291720005188 PubMed PMID: 33305716.
