# Supplementary material for: Dengue vaccine acceptability in Peru: A mixed-methods study in two dengue-endemic Peruvian cities
Source: PLoS Negl Trop Dis. 2026 May 18;20(5):e0013572. doi: 10.1371/journal.pntd.0013572 (PMC13193613; doi:10.1371/journal.pntd.0013572)
Supplement: S3 Text — (DOCX) [file pntd.0013572.s006.docx]

**S3 Text:**

**Description of the development and selection of Dengue Vaccine Hesitancy Outcomes:** **Analytical strategies used to model dengue vaccine hesitancy**

Given the absence of a validated scale specifically designed to measure dengue vaccine hesitancy at the time of data collection, as well as the minor modification introduced in one of the adapted items, and following the methodological recommendations proposed by Freeman et al. [1], multiple specifications of the dengue vaccine hesitancy outcome were developed and estimated. This approach aimed to characterize the hesitancy construct from different analytical perspectives.

Specifically, dengue vaccine hesitancy was operationalized in four complementary ways: (i) as a latent continuous construct estimated through confirmatory factor analysis; (ii) as an observed continuous score calculated as the mean of item-level responses; (iii) as a three-level categorical outcome classified as *willing to get vaccinated*, *unsure to get vaccinated*, and *strongly hesitant*; and (iv) as a dichotomous outcome derived from the three-level classification.

In this text, we describe the procedures used to construct each of these outcome specifications. In the subsequent text [S4 Text], we present systematic comparisons and sensitivity analyses across the four outcomes using the same set of predictors.

## **1. Latent continuous outcome: Dengue Vaccine Hesitancy Factor**

### ***1.1 Conceptual justification***

In line with contemporary theoretical frameworks, vaccine hesitancy was conceptualized as a continuous latent trait that is not directly observable but is instead reflected through a set of correlated attitudinal and behavioral indicators. Under this perspective, individual survey items represent imperfect manifestations of an underlying propensity toward vaccine acceptance, uncertainty, or resistance.

Modeling hesitancy as a latent construct offers several important advantages. First, it reduces measurement error by separating shared variance attributable to the underlying construct from item-specific noise. Second, it preserves information across the full range of responses, avoiding the loss of variability inherent in dichotomization or coarse categorization. Third, it mitigates concerns regarding arbitrary thresholding, a point explicitly raised by the reviewers in relation to outcome definition. This approach is consistent with the analytical strategy adopted in the original Oxford COVID-19 Vaccine Hesitancy Scale [1], which treated hesitancy as a graded phenomenon rather than a binary outcome.

### ***1.2 Model specification***

The latent dengue vaccine hesitancy factor was estimated using confirmatory factor analysis (CFA) [2,3] with a single latent factor representing overall vaccine hesitancy. Six adapted items were specified as ordered categorical indicators of this latent construct. All items were treated as ordinal variables, and model estimation was conducted using the weighted least squares mean- and variance-adjusted (WLSMV) estimator, as implemented in the lavaan package in R [4], which is appropriate for categorical or ordinal survey data and provides robust standard errors and fit statistics based on polychoric correlations and threshold estimation.

Responses coded as “Don’t know” (category 6) were recoded as missing at the item level, consistent with the conceptualization used in the original Oxford scale. Under the WLSMV framework, the CFA model was estimated using polychoric correlation matrices with pairwise present data, allowing respondents with partial item-level data to contribute to parameter estimation without requiring listwise deletion.

### ***1.3 Decision to exclude Item 1 from the latent model***

Although the original Oxford COVID-19 Vaccine Hesitancy Scale [1] comprised seven items, the latent dengue vaccine hesitancy factor was ultimately estimated using six items, excluding Item 1. This decision was based on a systematic comparison of seven-item and six-item confirmatory factor analysis (CFA) models and was driven by both conceptual coherence and empirical performance.

As described in S2 Text, Item 1 was modified during pilot testing to adopt a simplified trichotomous response format (Yes / No / Don’t know), departing from the ordinal response structure used in the remaining items. Although CFA with WLSMV permits indicators with differing numbers of ordinal categories, the simplified response structure of Item 1 substantially reduced its informational granularity relative to the remaining items and may have contributed to local dependency and estimation instability within the unidimensional model.

From a statistical perspective, inclusion of Item 1 resulted in estimation problems despite acceptable global fit indices. Specifically, the seven-item model produced a negative residual variance for Item 1 (a Heywood case), indicating model misspecification and undermining the interpretability of the latent construct. In addition, the seven-item model generated estimation warnings and yielded factor scores for a reduced subset of participants (n = 790/883), further indicating instability of the solution (Table A in S3 Text). In contrast, the six-item model demonstrated excellent overall fit, well-behaved residual variances, strong and statistically significant factor loadings, and stable factor score estimation. The latent dengue vaccine hesitancy outcome was therefore defined using six items to ensure valid, interpretable, and robust latent measurement suitable for multivariable regression analyses (Table A in S3 Text).

Table A in S3 Text. Comparison of CFA models for the Dengue Vaccine Hesitancy Scale

| **Indicator** | **7-item model**  **(including Item 1)** | **6-item model**  **(excluding Item 1)** |
| --- | --- | --- |
| Items included | 7 | 6 |
| Estimator | WLSMV | WLSMV |
| CFI | 0.998 | 0.999 |
| TLI | 0.997 | 0.998 |
| RMSEA | 0.054 | 0.046 |
| SRMR | 0.031 | 0.026 |
| Standardized factor loadings | 0.72–1.05 | 0.72–0.88 |
| Residual variances | Negative variance (Item 1) (-0.093) | All positive |
| Estimation warnings | Yes (Heywood case) | No |
| Factors score estimation | Unstable | Stable |
| Participants with latent scores | n = 790 of 883 | n = 835 of 883 |
| - WLSMV = *Weighted Least Squares Mean and Variance adjusted estimator*, appropriate for confirmatory factor analysis with ordinal categorical indicators. - CFI = *Comparative Fit Index* and TLI = *Tucker–Lewis Index*; values ≥0.95 indicate excellent model fit. - RMSEA = *Root Mean Square Error of Approximation*; values ≤0.06 indicate close fit and values ≤0.08 acceptable fit. - SRMR = *Standardized Root Mean Square Residual*; values ≤0.08 indicate good fit. - Negative residual variance indicates an improper solution (Heywood case), suggesting model misspecification and unreliable parameter estimation. | | |

### ***1.4 Model fit and interpretation***

The six-item one-factor CFA model demonstrated excellent overall fit to the data. Model fit indices indicated a strong correspondence between the specified latent structure and the observed response patterns (CFI ≈ 0.999; TLI ≈ 0.998; RMSEA ≈ 0.046; SRMR ≈ 0.026) (Table A in S3 Text). All six items loaded strongly and significantly onto the latent hesitancy factor, with standardized factor loadings ranging approximately from 0.72 to 0.88, indicating that each item contributed meaningfully to the measurement of the underlying construct. The resulting latent factor scores were approximately centered around zero, as expected under model identification constraints, with higher values indicating greater levels of dengue vaccine hesitancy.

Figure A in S3 Text. Distribution of latent dengue vaccine hesitancy (6 items)


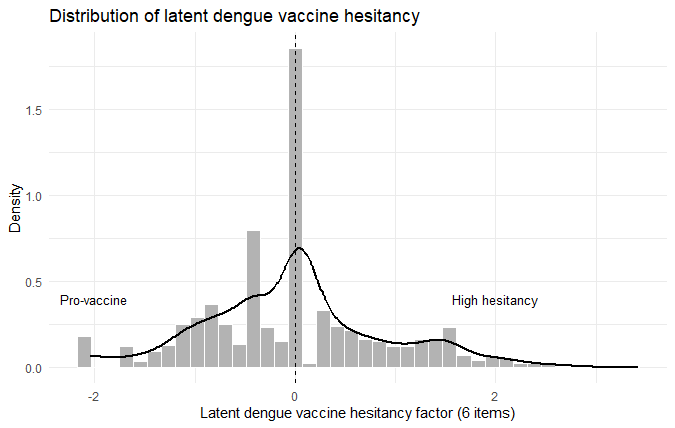


> summary(data$hesitancy_factor6)

Min. 1st Qu. Median Mean 3rd Qu. Max. NA's

-2.04494 -0.59211 0.03925 -0.01116 0.40536 3.42159 48

## **2. Observed continuous outcome: Hesitancy mean score**

### ***2.1 Construction***

As a complementary specification, dengue vaccine hesitancy was operationalized as an observed continuous outcome derived from participants’ responses to the adapted hesitancy items. Following the analytical strategy described by Freeman et al. (2021) [1], ordinal responses were interpreted as reflecting increasing levels of hesitancy, with lower values indicating vaccine acceptance and higher values indicating reluctance or refusal. Responses coded as “Don’t know” (value = 6) were not incorporated into the score and were treated as missing at the item level, rather than as intermediate or neutral responses. For each participant, the hesitancy mean score was calculated as the arithmetic mean of all available non-missing item responses. Importantly, the denominator corresponded to the number of valid responses provided by each participant, not to the total number of items. To ensure sufficient information for a stable and interpretable score while avoiding unnecessary exclusion due to uncertainty-driven missingness, the mean score was computed only for participants with valid responses on at least four of the seven items (min_valid_7 = 4). Participants with fewer than four valid responses were assigned missing values for this outcome.

### ***2.2 Role of Item 1***

Item 1 required specific handling due to its modified response structure. Whereas the remaining items retained a five-category ordinal scale, Item 1 was administered using a simplified trichotomous format (Yes / No / Don’t know). Prior to score construction, responses to Item 1 were recoded to align conceptually with the original hesitancy framework. Specifically, “Yes” responses were coded as 2 to reflect a pro-vaccine position, “No” responses—originally coded as 1—were recoded to 5 to represent clear vaccine hesitancy, and “Don’t know” responses—originally coded as 3—were recoded to 6 and treated as missing. This recoding allowed Item 1 to contribute information on general vaccine acceptance or refusal within the observed mean score, while maintaining consistency with the treatment of uncertainty applied to other items. Its inclusion in the observed continuous outcome therefore captured relevant behavioral intent, even though the item was excluded from the latent factor model for measurement-related reasons.

Figure B in S3 Text. Distribution of dengue vaccine hesitancy mean score (7 items)


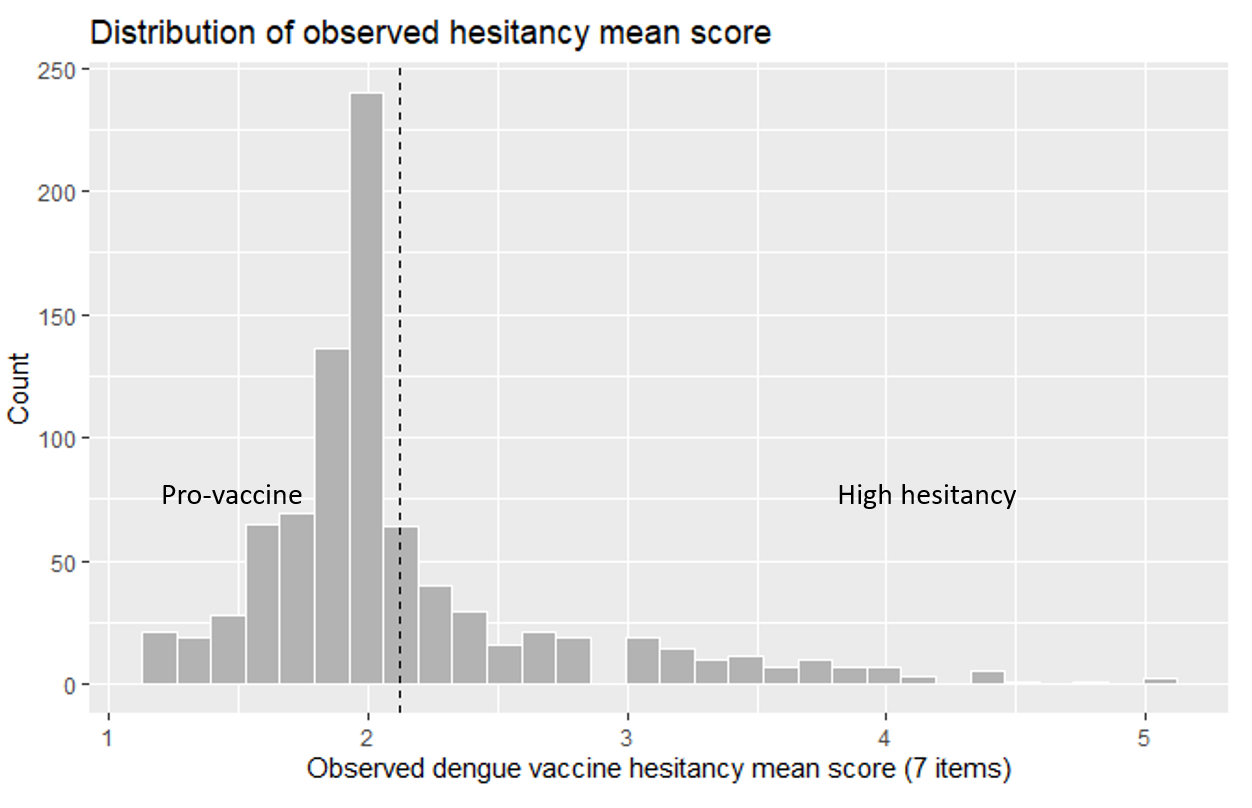


> summary(data$hesitancy_mean7)

Min. 1st Qu. Median Mean 3rd Qu. Max. NA's

1.143 1.857 2.000 2.124 2.286 5.000 19

## **3. Categorical outcome: Three-level dengue vaccine hesitancy**

### ***3.1 Classification rules***

As a third complementary specification, dengue vaccine hesitancy was operationalized as a three-level categorical outcome, closely following the rule-based classification strategy proposed by Freeman et al. (2021) for the Oxford COVID-19 Vaccine Hesitancy Scale [1]. This approach emphasizes consistency of responses across multiple items rather than reliance on a single question, thereby capturing meaningful and stable attitudinal patterns.

Each item was first positioned along the hesitancy continuum: clear pro-vaccine responses (ratings 1–2), neutral responses (rating 3), clear hesitant responses (ratings 4–5), and “Don’t know” responses (rating 6), which were treated as analytically distinct indicators of uncertainty. For Item 1, responses were recoded to align with this framework prior to classification.

Participants were then classified based on response predominance. Those endorsing four or more items with clear pro-vaccine responses were classified as *willing to get vaccinated*, whereas those endorsing four or more items with clear hesitant responses were classified as *strongly hesitant*. All remaining participants—including those with mixed, neutral, or uncertainty-dominated patterns—were classified as *unsure to get vaccinated*. This rule-based categorization reflects the conceptual logic of the original Oxford scale and preserves the graded nature of vaccine hesitancy. For clarity and consistency in the main manuscript, these three categories were relabeled as “acceptors” (willing to get vaccinated), “unsure” (unsure to get vaccinated), and “refusers” (strongly hesitant), while retaining the original classification logic described above.


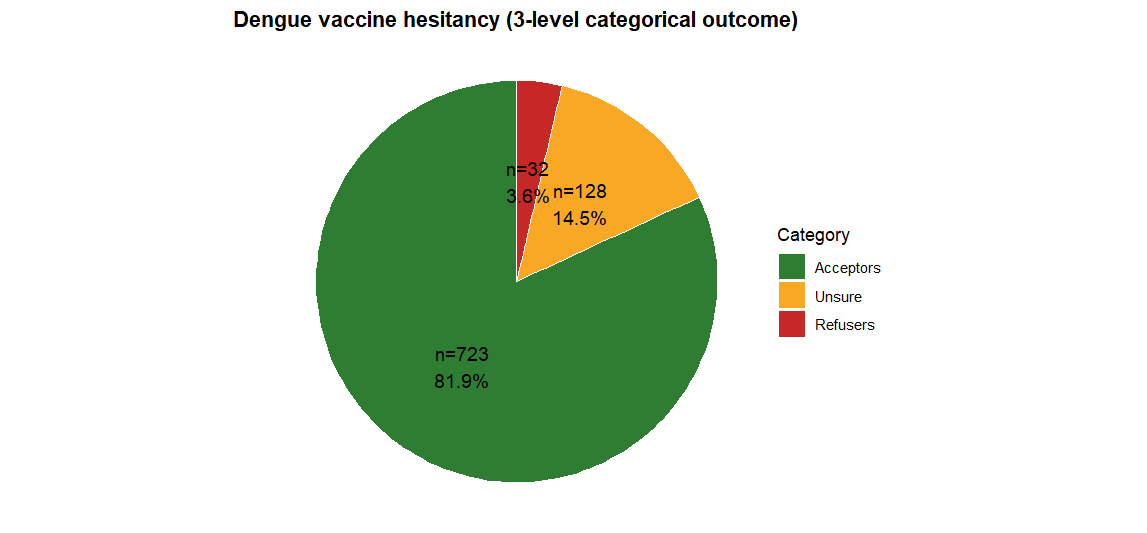
 Figure C in S3 Text. Dengue vaccine hesitancy (3-level categorical outcome).

## **4. Categorical outcome: Two-level dengue vaccine hesitancy**

### ***4.1 Conceptual and analytical rationale***

This fourth approach to operationalizing dengue vaccine hesitancy was developed based on the three-level categorical outcome described in Section 3 (*acceptors, unsure, and refusers*). Based on this classification, participants categorized as *refusers* (n = 32) were excluded, and a dichotomous outcome was constructed including participants classified as *acceptors* (n = 723) and *unsure* (n = 128).

The rationale for this specification is grounded in the widely accepted conceptualization of vaccine hesitancy as a continuum, in which the majority of the population tends to accept vaccination, an intermediate group expresses varying degrees of uncertainty or conditional acceptance, and a small minority consistently rejects all vaccines [5,6]. In this context, individuals who consistently reject all vaccines—representing a relatively small proportion of the population—constitute a conceptually distinct extreme, characterized by more stable attitudes that are less amenable to modification through informational or programmatic interventions.

From a public health and implementation perspective, primary interest often lies in the so-called movable middle, that is, individuals who do not categorically reject vaccination but instead express doubts, ambivalence, or conditions under which they would accept a new vaccine [7,8]. This intermediate group represents a substantial proportion of the population and constitutes the segment most likely to respond to communication strategies, trust-building efforts, and improvements in access to vaccination services.

Within this framework, constructing a dichotomous outcome contrasting *acceptors* versus *unsure* to get vaccinated allows for a more direct estimation of the determinants of vaccine uncertainty among individuals who do not exhibit unequivocal rejection. In addition, from an analytical standpoint, excluding the strongly hesitant group (*refusers*)—which was numerically small in our sample—helps reduce potential estimation instability in regression models, such as wide confidence intervals or imprecise estimates, without altering the substantive interpretation of the phenomenon under study.

**References.**

1. Freeman D, Loe BS, Chadwick A, Vaccari C, Waite F, Rosebrock L, et al. COVID-19 vaccine hesitancy in the UK: the Oxford coronavirus explanations, attitudes, and narratives survey (Oceans) II. Psychol Med. 2022 Oct 11;52(14):3127–41. doi:10.1017/S0033291720005188 PubMed PMID: 33305716.

2. Muthén B. A general structural equation model with dichotomous, ordered categorical, and continuous latent variable indicators. Psychometrika. 1984 Mar;49(1):115–32. doi:10.1007/BF02294210

3. Li CH. Confirmatory factor analysis with ordinal data: Comparing robust maximum likelihood and diagonally weighted least squares. Behavior Research Methods 2015 48:3. 2015 Jul 15;48(3):936–49. doi:10.3758/s13428-015-0619-7 PubMed PMID: 26174714.

4. Rosseel Y. lavaan: An R Package for Structural Equation Modeling. J Stat Softw. 2012 May 24;48:1–36. doi:10.18637/jss.v048.i02

5. MacDonald NE, Eskola J, Liang X, Chaudhuri M, Dube E, Gellin B, et al. Vaccine hesitancy: Definition, scope and determinants. Vaccine. 2015 Aug 14;33(34):4161–4. doi:10.1016/j.vaccine.2015.04.036 PubMed PMID: 25896383.

6. Dubé E, Laberge C, Guay M, Bramadat P, Roy R, Bettinger J. Vaccine hesitancy: An overview. Hum Vaccin Immunother. 2013 Aug;9(8):1763–73. doi:10.4161/hv.24657 PubMed PMID: 23584253.

7. Hyland P, Vallières F, Hartman TK, McKay R, Butter S, Bentall RP, et al. Detecting and describing stability and change in COVID-19 vaccine receptibility in the United Kingdom and Ireland. PLoS One. 2021 Nov 1;16(11):e0258871. doi:10.1371/journal.pone.0258871 PubMed PMID: 34731208.

8. Rozbroj T, McCaffery K. The importance of addressing social inequalities and targeting the undecided to promote vaccination against COVID-19. Lancet Reg Health West Pac. 2021 Sep 1;14:100250. doi:10.1016/j.lanwpc.2021.100250
