# Supplementary material for: Dengue vaccine acceptability in Peru: A mixed-methods study in two dengue-endemic Peruvian cities
Source: PLoS Negl Trop Dis. 2026 May 18;20(5):e0013572. doi: 10.1371/journal.pntd.0013572 (PMC13193613; doi:10.1371/journal.pntd.0013572)
Supplement: S4 Text — (DOCX) [file pntd.0013572.s007.docx]

**S4 Text:**

**Sensitivity analysis: Comparison of predictors across outcome specifications**

The sensitivity analysis aimed to assess the coherence and robustness of associations between key predictors and dengue vaccine hesitancy across four alternative outcome specifications derived from the same set of items and described in previous texts. This approach evaluated whether findings were dependent on the operational definition of hesitancy or reflected stable and consistent patterns of the underlying construct.

The analysis was conducted at two complementary levels. First, descriptive comparisons examined the correspondence between the three-level categorical outcome (*acceptors, unsure, and refusers*) and the two continuous outcomes (observed mean score and latent factor), focusing on distributional separation, ordering, and overlap. These comparisons confirmed that the three categories captured distinct positions along the vaccine hesitancy continuum (Table A in S4 Text).

Second, adjusted multivariable regression models were estimated for each of the four outcome specifications using an identical set of predictors, allowing comparison of the direction, relative magnitude, and statistical significance of associations across modeling approaches. Multiple linear regression was used for the continuous outcomes (latent factor and observed mean score), estimating beta coefficients as measures of association. Ordinal logistic regression was applied to the three-level categorical outcome under the proportional odds assumption, while binary logistic regression was used for the dichotomous outcome (*acceptors* vs. *unsure*).

Predictors were selected a priori and included variables aligned with the 5C model of vaccine hesitancy, along with key sociodemographic characteristics. Consistency of associations across outcome specifications was interpreted as evidence of robustness and conceptual validity beyond differences in scale and modeling assumptions.

## **1. General performance and coherence across outcome specifications**

This section presents descriptive comparisons showing consistent ordering of the three categories across both continuous outcomes. Figure A in S4 Text shows the distribution of the observed hesitancy mean score (7 items) across the three-category dengue vaccine hesitancy outcome. Participants classified as *acceptors* display consistently low mean scores, concentrated near the pro-vaccine end of the scale. In contrast, those classified as *refusers* show substantially higher mean scores, with minimal overlap with the willing group. Individuals categorized as *unsure* occupy an intermediate position, with scores spanning a wider range (Figure A in S4 Text).


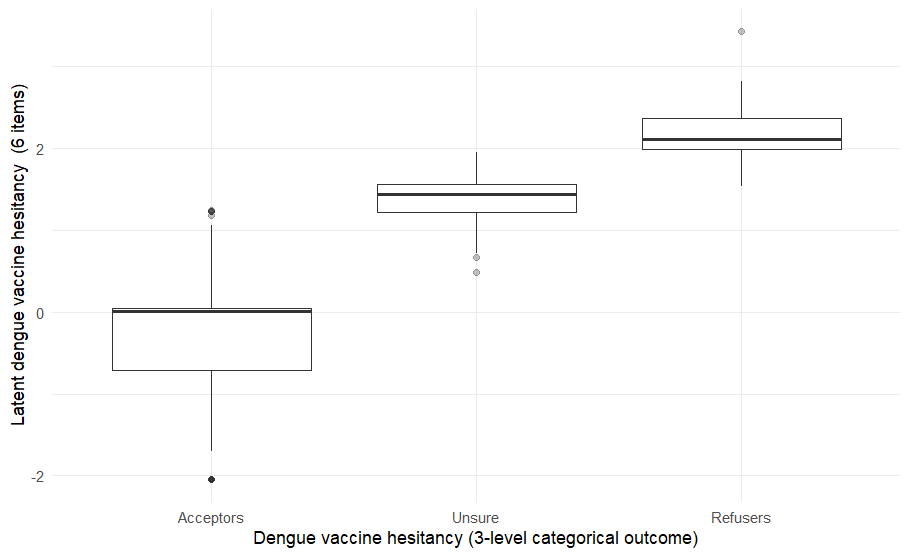
Figure A in S4 Text. Distribution of observed dengue vaccine hesitancy mean score by three-level categorical hesitancy outcome

Figure B in S4 Text presents the distribution of the latent dengue vaccine hesitancy factor (6 items, CFA-based) across the same three categorical groups. Negative latent scores are predominantly observed among individuals classified as *acceptors*, reflecting attitudes clearly more favorable than the average population. Conversely, *refusers* participants cluster at high positive latent values, indicating substantially elevated hesitancy. The *unsure* group again occupies an intermediate position, with moderate positive latent scores. The consistent separation and ordering of groups across the latent and observed outcomes demonstrate that all three specifications capture the same underlying hesitancy continuum, differing primarily in scale and modeling assumptions rather than substantive interpretation.


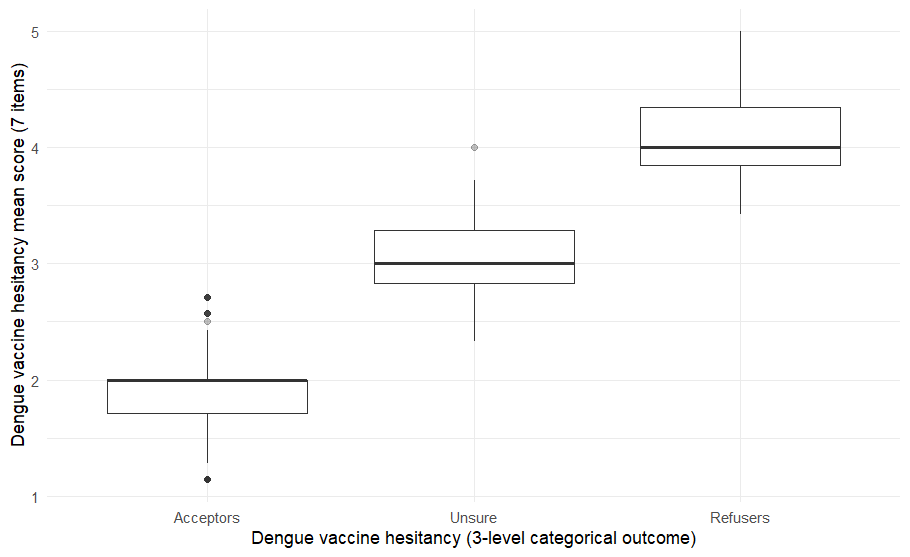
Figure B in S4 Text. Distribution of latent dengue vaccine hesitancy factor by three-level categorical hesitancy outcome

## **2. Multivariable regression results across outcome specifications**

In this section, we present the results of the adjusted multivariable regression models corresponding to the four outcome specifications. Associations between the selected predictors and dengue vaccine hesitancy are described and compared, with particular emphasis on the consistency of effects in terms of direction, relative magnitude, and statistical significance across the different models.

Across the four outcome specifications, associations showed strong consistency in direction and relative magnitude, indicating robust measurement of dengue vaccine hesitancy. Indicators of vaccine confidence—such as negative attitudes toward COVID-19 vaccination and concerns about rapid vaccine development—were consistently associated with higher hesitancy or uncertainty across models. Conversely, willingness to pay for the dengue vaccine and trust in institutional information sources showed stable protective associations. Although effect sizes were attenuated in the dichotomous outcome focused on uncertainty, the overall pattern of predictors remained coherent, supporting the conclusion that key determinants operate similarly across alternative operationalizations of hesitancy.

Table A in S4 Text. Consistency of predictors across four operational definitions of dengue vaccine hesitancy

|  | Latent hesitancy factor  (β) | | | Observed mean score  (β) | | | 3-Cat outcome  (OR) | | | 2-Cat outcome  (OR) | | |
| --- | --- | --- | --- | --- | --- | --- | --- | --- | --- | --- | --- | --- |
| **Characteristic** | **Beta** | **95% CI** | **p-value** | **Beta** | **95% CI** | **p-value** | **OR** | **95% CI** | **p-value** | **OR** | **95% CI** | **p-value** |
| **CONFIDENCE** |  |  |  |  |  |  |  |  |  |  |  |  |
| **Opinion of the COVID-19 vaccine** |  |  | <0.001 |  |  | <0.001 |  |  | <0.001 |  |  | 0.007 |
| *In favor* | — | — |  | — | — |  | — | — |  | — | — |  |
| *Against or in doubt* | 0.34 | 0.17, 0.50 |  | 0.30 | 0.19, 0.41 |  | 3.09 | 1.87, 5.11 |  | 2.17 | 1.23, 3.79 |  |
| **Trust in vaccine benefits (Vaccination Attitudes Examination [VAX] scale construct)** |  |  | 0.013 |  |  | <0.001 |  |  | <0.001 |  |  | 0.001 |
| *Low* | — | — |  | — | — |  | — | — |  | — | — |  |
| *Intermediate* | -0.25 | -0.54, 0.04 |  | -0.32 | -0.51, -0.13 |  | 0.45 | 0.21, 0.95 |  | 0.52 | 0.21, 1.27 |  |
| *High* | -0.39 | -0.67, -0.10 |  | -0.42 | -0.60, -0.24 |  | 0.24 | 0.12, 0.52 |  | 0.25 | 0.10, 0.61 |  |
| **Knowing that the COVID-19 vaccine was developed very quickly reduced my confidence in getting vaccinated** |  |  | 0.006 |  |  | <0.001 |  |  | 0.010 |  |  | 0.015 |
| *No* | — | — |  | — | — |  | — | — |  | — | — |  |
| *Yes* | 0.27 | 0.08, 0.46 |  | 0.23 | 0.10, 0.35 |  | 2.06 | 1.18, 3.56 |  | 2.17 | 1.17, 3.98 |  |
| **Concerns about unforeseen future effects of vaccines (Vaccination Attitudes Examination [VAX] scale construct)** |  |  | >0.9 |  |  | >0.9 |  |  | 0.6 |  |  | 0.2 |
| *Low* | — | — |  | — | — |  | — | — |  | — | — |  |
| *Intermediate* | 0.03 | -0.17, 0.23 |  | 0.00 | -0.14, 0.13 |  | 1.41 | 0.63, 3.49 |  | 2.39 | 0.89, 7.83 |  |
| *High* | 0.04 | -0.17, 0.25 |  | -0.02 | -0.16, 0.12 |  | 1.21 | 0.52, 3.07 |  | 2.36 | 0.85, 7.92 |  |
| **COMPLACENCY** |  |  |  |  |  |  |  |  |  |  |  |  |
| **Participant believes they could get dengue again if previously infected (Agree)** |  |  | 0.12 |  |  | 0.4 |  |  | 0.6 |  |  | 0.2 |
| *Disagree* | — | — |  | — | — |  | — | — |  | — | — |  |
| *Agree* | -0.10 | -0.23, 0.03 |  | -0.03 | -0.12, 0.05 |  | 0.89 | 0.57, 1.39 |  | 0.71 | 0.43, 1.16 |  |
| **Participant believes people can die from dengue infection (Agree)** |  |  | 0.017 |  |  | 0.003 |  |  | 0.020 |  |  | 0.12 |
| *Disagree* | — | — |  | — | — |  | — | — |  | — | — |  |
| *Agree* | -0.36 | -0.65, -0.06 |  | -0.29 | -0.48, -0.09 |  | 0.42 | 0.20, 0.88 |  | 0.49 | 0.21, 1.20 |  |
| **Participant perceives that dengue infection could lead to a loss of income (Agree)** |  |  | >0.9 |  |  | 0.7 |  |  | 0.4 |  |  | 0.5 |
| *Disagree* | — | — |  | — | — |  | — | — |  | — | — |  |
| *Agree* | 0.00 | -0.18, 0.18 |  | -0.02 | -0.14, 0.10 |  | 0.80 | 0.47, 1.41 |  | 0.80 | 0.43, 1.51 |  |
| **Preference for natural immunity** |  |  | 0.11 |  |  | 0.2 |  |  | 0.4 |  |  | 0.5 |
| *Low* | — | — |  | — | — |  | — | — |  | — | — |  |
| *Intermediate* | 0.06 | -0.08, 0.20 |  | 0.07 | -0.02, 0.16 |  | 1.32 | 0.81, 2.16 |  | 1.28 | 0.75, 2.21 |  |
| *High* | -0.10 | -0.26, 0.05 |  | -0.02 | -0.12, 0.08 |  | 0.96 | 0.53, 1.74 |  | 0.90 | 0.46, 1.74 |  |
| **CONVENIENCE** |  |  |  |  |  |  |  |  |  |  |  |  |
| **Willingness to pay for a dengue vaccine** |  |  | <0.001 |  |  | <0.001 |  |  | <0.001 |  |  | <0.001 |
| *No* | — | — |  | — | — |  | — | — |  | — | — |  |
| *Yes* | -0.37 | -0.49, -0.25 |  | -0.24 | -0.32, -0.16 |  | 0.32 | 0.19, 0.53 |  | 0.40 | 0.23, 0.67 |  |
| **Willingness to wait in line at dengue vaccination site** |  |  | 0.4 |  |  | 0.4 |  |  | >0.9 |  |  | 0.3 |
| *Less than one hour* | — | — |  | — | — |  | — | — |  | — | — |  |
| *One hour or more* | 0.05 | -0.08, 0.18 |  | 0.04 | -0.05, 0.12 |  | 0.98 | 0.62, 1.55 |  | 0.76 | 0.46, 1.26 |  |
| **COMMUNICATION** |  |  |  |  |  |  |  |  |  |  |  |  |
| **Negative information or misinformation encountered through news media or social networks was a major factor contributing to my distrust of COVID-19 vaccination** |  |  | 0.4 |  |  | >0.9 |  |  | 0.6 |  |  | 0.8 |
| *No* | — | — |  | — | — |  | — | — |  | — | — |  |
| *Yes* | 0.05 | -0.07, 0.17 |  | 0.00 | -0.08, 0.08 |  | 0.90 | 0.58, 1.38 |  | 0.95 | 0.59, 1.54 |  |
| **Before deciding whether to receive a dengue vaccine, I would like to know whether it has been officially approved by Ministry of Health or WHO** |  |  | 0.9 |  |  | 0.7 |  |  | 0.15 |  |  | 0.11 |
| *No* | — | — |  | — | — |  | — | — |  | — | — |  |
| *Yes* | -0.02 | -0.25, 0.22 |  | 0.03 | -0.12, 0.19 |  | 1.68 | 0.82, 3.36 |  | 1.89 | 0.87, 3.98 |  |
| **Preference for Ministry of Health as source of dengue vaccine information** |  |  | 0.2 |  |  | 0.036 |  |  | 0.094 |  |  | 0.11 |
| *No* | — | — |  | — | — |  | — | — |  | — | — |  |
| *Yes* | -0.10 | -0.23, 0.04 |  | -0.10 | -0.19, -0.01 |  | 0.65 | 0.40, 1.08 |  | 0.63 | 0.36, 1.11 |  |
| **Health centers or local primary care facilities as source of dengue vaccine information** |  |  | <0.001 |  |  | <0.001 |  |  | 0.007 |  |  | 0.057 |
| *No* | — | — |  | — | — |  | — | — |  | — | — |  |
| *Yes* | -0.28 | -0.42, -0.13 |  | -0.22 | -0.31, -0.12 |  | 0.47 | 0.26, 0.81 |  | 0.55 | 0.29, 1.02 |  |
| **Media to publicize the vaccine** |  |  | 0.058 |  |  | 0.3 |  |  | 0.4 |  |  | 0.4 |
| *Radio* | — | — |  | — | — |  | — | — |  | — | — |  |
| *Tv* | -0.08 | -0.29, 0.14 |  | -0.05 | -0.20, 0.09 |  | 0.58 | 0.25, 1.33 |  | 0.89 | 0.35, 2.23 |  |
| *Social media* | -0.12 | -0.31, 0.07 |  | -0.05 | -0.18, 0.07 |  | 0.86 | 0.45, 1.68 |  | 1.39 | 0.66, 2.99 |  |
| *Perifoneo* | 0.01 | -0.18, 0.20 |  | -0.01 | -0.13, 0.12 |  | 1.28 | 0.65, 2.54 |  | 1.80 | 0.84, 3.99 |  |
| *House-to-house notification* | 0.22 | -0.02, 0.45 |  | 0.11 | -0.05, 0.27 |  | 1.28 | 0.53, 3.01 |  | 1.08 | 0.37, 2.97 |  |
| **CONTEX** |  |  |  |  |  |  |  |  |  |  |  |  |
| **Site of study** |  |  | 0.4 |  |  | 0.10 |  |  | 0.2 |  |  | 0.4 |
| *Iquitos* | — | — |  | — | — |  | — | — |  | — | — |  |
| *Piura* | 0.06 | -0.10, 0.23 |  | 0.09 | -0.02, 0.20 |  | 1.46 | 0.79, 2.70 |  | 1.34 | 0.68, 2.67 |  |
| **Previous participation in dengue research** |  |  | 0.3 |  |  | >0.9 |  |  | 0.6 |  |  | 0.4 |
| *No* | — | — |  | — | — |  | — | — |  | — | — |  |
| *Yes* | 0.07 | -0.06, 0.19 |  | 0.00 | -0.08, 0.08 |  | 0.89 | 0.57, 1.40 |  | 0.82 | 0.50, 1.35 |  |
| **Close experience with severe covid (Yes)** |  |  | 0.8 |  |  | 0.6 |  |  | 0.8 |  |  | 0.2 |
| *No* | — | — |  | — | — |  | — | — |  | — | — |  |
| *Yes* | -0.02 | -0.14, 0.11 |  | 0.02 | -0.06, 0.11 |  | 1.05 | 0.66, 1.67 |  | 0.73 | 0.43, 1.23 |  |
| **Close experience with severe dengue (Yes)** |  |  | 0.5 |  |  | 0.7 |  |  | 0.4 |  |  | 0.2 |
| *No* | — | — |  | — | — |  | — | — |  | — | — |  |
| *Yes* | -0.06 | -0.23, 0.11 |  | -0.02 | -0.13, 0.09 |  | 1.35 | 0.71, 2.50 |  | 1.52 | 0.75, 2.97 |  |
| **COVID-19 vaccine doses** |  |  | <0.001 |  |  | 0.003 |  |  | 0.015 |  |  | 0.087 |
| *Third or fourth doses* | — | — |  | — | — |  | — | — |  | — | — |  |
| *No or incomplete doses* | 0.29 | 0.12, 0.46 |  | 0.17 | 0.06, 0.28 |  | 1.87 | 1.12, 3.08 |  | 1.66 | 0.93, 2.93 |  |
| **Know how dengue is transmitted (Yes)** |  |  | 0.3 |  |  | 0.2 |  |  | 0.001 |  |  | 0.030 |
| *No* | — | — |  | — | — |  | — | — |  | — | — |  |
| *Yes* | -0.10 | -0.29, 0.10 |  | -0.09 | -0.22, 0.04 |  | 0.36 | 0.19, 0.67 |  | 0.45 | 0.23, 0.93 |  |
| **SOCIODEMOGRAPHIC** |  |  |  |  |  |  |  |  |  |  |  |  |
| **Gender** |  |  | 0.2 |  |  | 0.3 |  |  | 0.3 |  |  | 0.10 |
| *Female* | — | — |  | — | — |  | — | — |  | — | — |  |
| *Male* | 0.09 | -0.06, 0.25 |  | 0.05 | -0.05, 0.15 |  | 1.34 | 0.80, 2.27 |  | 1.63 | 0.91, 2.93 |  |
| **Age in categories** |  |  | 0.049 |  |  | 0.5 |  |  | 0.6 |  |  | 0.6 |
| *18 to 39 years old* | — | — |  | — | — |  | — | — |  | — | — |  |
| *40 to 60 years old* | -0.12 | -0.24, 0.00 |  | -0.03 | -0.11, 0.06 |  | 0.87 | 0.56, 1.36 |  | 0.88 | 0.54, 1.44 |  |
| **Level education** |  |  | 0.4 |  |  | 0.12 |  |  | 0.003 |  |  | 0.031 |
| *Elementary school* | — | — |  | — | — |  | — | — |  | — | — |  |
| *High school* | 0.05 | -0.13, 0.23 |  | 0.08 | -0.04, 0.19 |  | 2.84 | 1.31, 6.56 |  | 1.81 | 0.81, 4.32 |  |
| *Technical and/or higher* | 0.15 | -0.07, 0.36 |  | 0.15 | 0.01, 0.29 |  | 4.89 | 2.00, 12.7 |  | 3.33 | 1.31, 8.97 |  |
| **Occupation** |  |  | 0.068 |  |  | 0.5 |  |  | 0.7 |  |  | 0.6 |
| *Homemaker* | — | — |  | — | — |  | — | — |  | — | — |  |
| *Qualified worker* | 0.21 | 0.02, 0.39 |  | 0.07 | -0.05, 0.19 |  | 1.22 | 0.63, 2.38 |  | 1.10 | 0.52, 2.29 |  |
| *No Qualified worker* | 0.15 | -0.02, 0.32 |  | 0.05 | -0.07, 0.16 |  | 1.34 | 0.71, 2.50 |  | 1.41 | 0.70, 2.84 |  |
| *No. Obs.*  *R^2^* |  | 748  0.276 |  |  | 773  0.297 |  |  | 790 |  |  | 761 |  |
| Abbreviations: CI = Confidence Interval, OR = Adjusted Odds Ratio, β = Regression coefficient from linear regression models (continuous outcomes) | | | | | | | | | | | | |
