# Supplementary material for: Dengue vaccine acceptability in Peru: A mixed-methods study in two dengue-endemic Peruvian cities
Source: PLoS Negl Trop Dis. 2026 May 18;20(5):e0013572. doi: 10.1371/journal.pntd.0013572 (PMC13193613; doi:10.1371/journal.pntd.0013572)
Supplement: S5 Text — (DOCX) [file pntd.0013572.s008.docx]

**S5 Text:**

**Description of** **Methodological Limitations**

This study has several methodological limitations that warrant transparent discussion. First, the hesitancy instrument represents an adaptation of the Oxford COVID-19 Vaccine Hesitancy Scale to the dengue context, and a formal psychometric validation specific to dengue vaccination was not conducted. Second, one item required substantive modification in both wording and response format, which prevented full preservation of its original ordinal structure. Third, data were collected in a pre-implementation setting, such that attitudes toward a hypothetical dengue vaccine may differ from those observed following vaccine rollout.

Importantly, these limitations are mitigated by the use of multiple, complementary outcome specifications and the consistency of results across them. As shown in the comparative analyses, key predictors—including indicators of vaccine confidence, attitudes toward COVID-19 vaccination, and willingness to pay—exhibited stable directions of association and comparable relative magnitudes across categorical, latent, observed continuous, and dichotomous outcomes. The convergence of findings across these analytically distinct approaches provides strong evidence of internal coherence and construct validity, suggesting that substantive conclusions are not artifacts of any single operational definition or modeling assumption.

While retaining the original response structure for the modified item would have been preferable, the harmonization strategy was explicitly documented in the supplementary material, and sensitivity analyses demonstrated that results were robust to alternative outcome specifications. Collectively, these findings support the feasibility of further scale refinement and formal psychometric validation in future work.

Given the observed consistency across outcomes, the main analyses presented in the manuscript focus on the dichotomous outcome contrasting *acceptors* versus *unsure*. This specification was selected to facilitate interpretation and to emphasize programmatically relevant determinants that may shift individuals in the “uncertain” group toward vaccine acceptance, a population segment most amenable to public health intervention [1,2].

# **References.**

1. Hyland P, Vallières F, Hartman TK, McKay R, Butter S, Bentall RP, et al. Detecting and describing stability and change in COVID-19 vaccine receptibility in the United Kingdom and Ireland. PLoS One. 2021 Nov 1;16(11):e0258871. doi:10.1371/journal.pone.0258871 PubMed PMID: 34731208.

2. Rozbroj T, McCaffery K. The importance of addressing social inequalities and targeting the undecided to promote vaccination against COVID-19. Lancet Reg Health West Pac. 2021 Sep 1;14:100250. doi:10.1016/j.lanwpc.2021.100250
