# Supplementary material for: Dengue vaccine acceptability in Peru: A mixed-methods study in two dengue-endemic Peruvian cities
Source: PLoS Negl Trop Dis. 2026 May 18;20(5):e0013572. doi: 10.1371/journal.pntd.0013572 (PMC13193613; doi:10.1371/journal.pntd.0013572)
Supplement: S6 Text — (DOCX) [file pntd.0013572.s009.docx]

**S6 Text:**

**Characterization of Participants Excluded from the Multivariable Model due to Missing Covariate Data (Complete-Case Analysis)**

## **1. Overview**

Of the 851 participants classified as acceptors or unsure (i.e., the analytic sample for the multivariable model, after excluding 32 refusers from the descriptive sample of 883), 90 (10.6%) were excluded due to missing data in one or more covariates, resulting in a final analytic sample of n = 761 (Piura = 409; Iquitos = 352). Missing data were concentrated across 10 covariates, with the majority attributable to a single attitudinal variable (av_trust, confidence in vaccine benefits). Table A in S6 Text shows the distribution of missing data by covariate, and Table B in S6 Text presents a systematic comparison of included versus excluded participants across key demographic and contextual characteristics.

Table A in S6 Text. Distribution of Missing Data by Covariate Among Excluded Participants (n=90)

| **Covariate with missing data** | **n**  **missing** | **% of excluded (n=90)** | **% of analytic sample (n=851)** |
| --- | --- | --- | --- |
| Trust in vaccine benefits (VAX scale construct) | 63 | 70.0% | 7.4% |
| Media to publicize the vaccine | 13 | 14.4% | 1.5% |
| Preference for natural immunity (VAX scale construct) | 12 | 13.3% | 1.4% |
| Occupation | 6 | 6.7% | 0.7% |
| Concerns about unforeseen future effects of vaccines (VAX scale construct) | 5 | 5.6% | 0.6% |
| Participant perceives dengue may lead to income loss | 2 | 2.2% | 0.2% |
| Know how dengue is transmitted | 2 | 2.2% | 0.2% |
| Participant believes they could get dengue again if previously infected | 1 | 1.1% | 0.1% |
| Participant believes people can die from dengue infection | 1 | 1.1% | 0.1% |
| Willingness to wait in line at dengue vaccination site | 1 | 1.1% | 0.1% |

*Note: Percentages do not sum to 100% because some participants had missing data in more than one covariate.*

## **2. Comparison of Included vs. Excluded Participants**

Table B in S6 Text: Characteristics of Participants Included vs. Excluded from the Multivariable Model

| **Variable** | **N** | **Overall** N = 851^1^ | **Included** n = 761^1^ | **Excluded** n = 90^1^ | **p-value**^2^ |
| --- | --- | --- | --- | --- | --- |
| **Study site** | 851 |  |  |  | <0.001 |
| *Iquitos* |  | 414 (49%) | 352 (46%) | 62 (69%) |  |
| *Piura* |  | 437 (51%) | 409 (54%) | 28 (31%) |  |
| **Age group** | 851 |  |  |  | 0.202 |
| *18 to 39 years old* |  | 437 (51%) | 397 (52%) | 40 (44%) |  |
| *40 to 60 years old* |  | 414 (49%) | 364 (48%) | 50 (56%) |  |
| **Education level** | 851 |  |  |  | 0.507 |
| *Elementary school* |  | 144 (17%) | 132 (17%) | 12 (13%) |  |
| *High school* |  | 387 (45%) | 347 (46%) | 40 (44%) |  |
| *Technical and/or higher* |  | 320 (38%) | 282 (37%) | 38 (42%) |  |
| **Sex** | 851 |  |  |  | 0.377 |
| *Female* |  | 606 (71%) | 546 (72%) | 60 (67%) |  |
| *Male* |  | 245 (29%) | 215 (28%) | 30 (33%) |  |
| **Occupation** | 845 |  |  |  | 0.498 |
| *Homemaker* |  | 381 (45%) | 348 (46%) | 33 (39%) |  |
| *Qualified worker* |  | 274 (32%) | 245 (32%) | 29 (35%) |  |
| *No Qualified worker* |  | 190 (22%) | 168 (22%) | 22 (26%) |  |
| **Previous experience in dengue research (Yes)** | 851 | 408 (48%) | 367 (48%) | 41 (46%) | 0.713 |
| **Dengue vaccine hesitancy** | 851 |  |  |  | 0.525 |
| *Vaccination “acceptors”* |  | 723 (85%) | 644 (85%) | 79 (88%) |  |
| *Unsure to get vaccinated* |  | 128 (15%) | 117 (15%) | 11 (12%) |  |
| ^1^n (%) | | | | | |
| ^2^Pearson's Chi-squared test | | | | | |

*Note: n (%); Pearson's Chi-squared test. *Statistically significant (p < 0.05). Red p-values indicate statistically significant differences.*

## **3. Interpretation of Results**

**Primary source of missing data**

Missing data were predominantly attributable to a single attitudinal variable, confidence in vaccine benefits (a VAX scale subscale), which accounted for 70.0% (n = 63) of all excluded participants. The remaining nine covariates contributed minimally, with no single variable accounting for more than 14.4% of exclusions (media to publicize the vaccine, n = 13). This pattern indicates that missing data were structurally concentrated rather than diffusely distributed across the covariate set.

**Site-level differences in exclusion rates**

The only statistically significant difference between included and excluded participants was study site (p < 0.001). Exclusion rates differed substantially between sites: 15.0% of Iquitos participants were excluded (n = 62 of 414) compared to 6.4% in Piura (n = 28 of 437). Stratified analysis confirms that this differential was driven by item non-response to the same variable at both sites: confidence in vaccine benefits accounted for 66.1% of exclusions in Iquitos (n = 41 of 62) and 78.6% in Piura (n = 22 of 28). The higher overall exclusion rate in Iquitos therefore reflects a greater volume of non-response to this specific attitudinal construct, not a broader or more diffuse pattern of missing data.

**Absence of systematic bias in demographic and contextual characteristics**

Excluded participants did not differ significantly from included participants by age group (p = 0.202), education level (p = 0.507), sex (p = 0.377), occupation (p = 0.498), or previous experience in dengue research (p = 0.713). The absence of differential exclusion by this variable indicates that the key finding is unlikely to be an artifact of the complete-case analytic strategy.

**Outcome distribution comparability**

The proportion classified as vaccine-uncertain did not differ between included (15%) and excluded (12%) participants (p = 0.525). Preservation of the outcome distribution across both groups indicates that the complete-case sample does not systematically overrepresent or underrepresent either outcome category, supporting the internal validity of the primary estimates.

**Overall assessment**

The pattern of missing data is consistent with a Missing At Random (MAR) mechanism conditional on study site. The higher exclusion rate observed in Iquitos was not accompanied by differences in any demographic, occupational, or outcome-relevant characteristic between included and excluded participants. Since study site is an observed variable included as a covariate in the adjusted model, the MAR assumption is plausibly satisfied within the analytic framework employed. The complete-case analysis (n = 761) is therefore considered analytically sound. Sensitivity analyses using alternative outcome specifications — a continuous latent score, an observed mean score, and a three-level ordinal categorization — produced results consistent with the primary model, further supporting the robustness of the findings.
