# Supplementary material for: Dengue vaccine acceptability in Peru: A mixed-methods study in two dengue-endemic Peruvian cities
Source: PLoS Negl Trop Dis. 2026 May 18;20(5):e0013572. doi: 10.1371/journal.pntd.0013572 (PMC13193613; doi:10.1371/journal.pntd.0013572)
Supplement: Attachment — Submitted filename: PNTD-D-25-01673_reviewer_report_submitted.pdf [file pntd.0013572.s010.pdf]

Reviewer Report: PNTD-D-25-01673

Manuscript Title: Dengue vaccine acceptability in Peru: A mixed-methods study in two dengue-endemic Peruvian cities

Journal: PLOS Neglected Tropical Diseases

Overall Recommendation: MAJOR REVISION with strong encouragement for a follow-up validation study.

Summary and General Assessment

This is a timely and valuable mixed-methods study that provides crucial pre-implementation data on dengue vaccine acceptability in high-burden Peruvian regions, framed by the **5C model of vaccine hesitancy**. The combination of focus groups and a survey is appropriate for the research question. The finding of **81.2% stated willingness** to be vaccinated establishes an important baseline. However, the strength of the conclusions is significantly constrained by a critical **temporal disconnect** (2022 data collection vs. 2024 Qdenga rollout) and several substantial methodological issues related to statistical rigor and outcome definition. Addressing these concerns is essential for the manuscript to provide actionable and credible guidance.

Major Concerns Requiring Substantial Revision

1. Temporal Disconnect and Research Opportunity

The core limitation is the gap between surveying attitudes toward a **hypothetical vaccine** in 2022 and the **actual Qdenga rollout** in 2024.

- **Validity Issue:** Attitudes were likely heavily influenced by the immediate **COVID-19 context** (e.g., mandates, political polarization), which may temporarily inflate hesitancy and may not translate to real-world dengue vaccine uptake.
- **Required Action:** The paper must be **reframed as pre-implementation baseline data**. The discussion should explicitly address how the post-COVID context may skew the findings and how the shift from a hypothetical to a real vaccine may change behavior.
- **Strong Encouragement:** The authors have an extraordinary opportunity to transform this work by committing to a rapid **follow-up validation study** to compare their 2022 predictions against 2024-25 **actual Qdenga uptake** in their study sites. This would elevate the paper's contribution to high-impact implementation science.

2. Problematic Outcome Variable Definition

The study's **dichotomization of vaccine acceptance** is methodologically flawed because it conflates distinct concepts, leading to potential misclassification and limited policy relevance.
The definition groups participants as "Willing" if they responded:

- "I would get it as soon as I can" (Immediate Acceptance)
- **"I would get it when I have time" (Convenience Barrier)**

The phrase **"when I have time"** strongly indicates a *logistical or convenience barrier* (e.g., work conflicts, travel distance, long clinic queues), not true **vaccine hesitancy** (which is typically defined as delay or refusal due to psychological or cognitive factors like lack of confidence or complacency).
This conflation has three major consequences:

1. **Misclassification and Inflated Rates:** It likely misclassifies participants who face access challenges as fully "willing," which artificially **inflates the overall acceptance rate** (reported at 81.2%).

2. **Obscured Policy Relevance:** By treating convenience barriers as acceptance, the analysis **obscures critical logistical hurdles** that implementation strategies must address (e.g., mobile clinics, evening hours).
3. **Loss of Nuance:** Converting a nuanced, multi-point scale into a simple binary variable sacrifices valuable information about the continuum of acceptance and refusal.

Required Changes:
The authors must either:

1. **Conduct Sensitivity Analyses:** Test alternative models by treating "I would get it when I have time" as a separate category, or by grouping it with "I would delay getting it" (a form of logistical hesitancy).
2. **Treat as Ordinal:** Analyze the outcome as an **ordinal variable** (preserving all response categories) to capture the full nuance of participant intent and proximity to refusal.
3. **Justify Categorization:** If the current categorization is retained, provide a **clear, evidence-based justification** by referencing established, validated vaccine acceptance scales.

3. Unexplained and Counterintuitive Finding: Education and Hesitancy

The finding that **technical/higher education is associated with dramatically increased hesitancy (OR 6.48)** is highly unusual and requires deep, data-driven exploration.

- **Problem:** The provided discussion is insufficient. This large effect could signal confounding, selection bias, or a genuine phenomenon (e.g., greater exposure to online misinformation or distrust among critical, educated groups).
- **Required Analysis:** Provide a **deeper analysis** exploring mechanisms: test for **interaction effects** with information sources and trust measures and systematically integrate **qualitative data mining** to find education-specific themes.

4. Statistical Analysis Deficiencies (Critical)

Confidence in the final model's predictors is undermined by several critical reporting omissions:

- **Missing Data:** The final model excludes **29% of participants** without any description of missing data patterns, handling strategy (e.g., imputation), or sensitivity analyses. This raises a significant risk of selection bias. A **missing data flow diagram** is required.
- **Model Building:** The process for variable selection is unclear. The authors must provide a **transparent description of the model building strategy** and justify variable inclusion/exclusion.
- **Model Diagnostics: VIF statistics** are necessary to check for multicollinearity among correlated variables (e.g., education, wealth, residence).
- **Effect Modification:** Given the substantial difference in hesitancy between sites (Piura 13.8% vs. Iquitos 23.8%), **interaction terms (site × predictor)** must be tested to explore effect modification.
- **Clarity:** Ensure **consistent interpretation** of Odds Ratios (ORs) throughout the abstract and results (i.e., always clearly state if the OR represents hesitancy or acceptance).

5. Weak Integration of Mixed Methods

The study describes itself as mixed methods, but the integration is primarily sequential (qual → quant) rather than analytical.

- **Required Action:** Use the qualitative data more effectively to **explain contradictory quantitative findings**. For instance, why did FGDs reveal trust in health professionals if this was not a significant predictor in the regression model? A **convergence matrix or joint display** is needed to systematically compare and reconcile qualitative and quantitative results.

Moderate and Minor Concerns

- **Sampling and Generalizability:** The **daytime household recruitment** resulted in an oversampling of women (**71% female**) and likely under samples working-age males. The authors must acknowledge this **selection bias** and discuss its implication for generalizability.

- **Measurement:** The adaptation of the VAX scale from 7-point to 5-point without validation requires reporting of **reliability statistics** (e.g., Cronbach's alpha). The operationalization of the "dengue knowledge" measure must be fully described.
- **Presentation and Clarity: Figure 1** is difficult to read and should be redesigned. **Table 3** should clearly separate analytical themes from illustrative quotes.
- **Discussion and Recommendations:** The recommendations must be more **concrete, context-specific, and actionable** (e.g., specifying messaging for the two-dose schedule or how to target the concerns of higher-educated individuals).
- **Literature Engagement:** Strengthen the discussion of existing **Latin American dengue vaccine acceptance literature** and address factors specific to the new vaccine, such as the two-dose schedule and seronegativity approval.

Recommendations for Resubmission

The paper must address the following points with accompanying new analysis and transparent reporting:

1. **Reframing and Temporal Acknowledgment** (Line 88-98). Reframe as pre-implementation baseline; explicitly discuss the COVID-19 proxy issue and the need for validation.
2. **Outcome Definition Revision/Justification** (Line 223-228). Conduct sensitivity analyses for the definition; justify or revise categorization of "when I have time."
3. **Complete Statistical Methods.** Address missing data (29% excluded), provide model building transparency, report VIFs, and test for site × predictor interaction (effect modification).
4. **Deeper Analysis of Education-Hesitancy Link.** Deeper analysis, including qualitative integration and stratified analysis, to explain the high OR for hesitancy.
5. **Systematic Mixed-Methods Integration**. Create a **joint display** or convergence matrix to systematically compare and explain discrepancies between qualitative and quantitative findings.

The combination of addressing these major revisions and pursuing a validation study would make this an exceptional piece of implementation science.
