# Supplementary material for: Dengue vaccine acceptability in Peru: A mixed-methods study in two dengue-endemic Peruvian cities
Source: PLoS Negl Trop Dis. 2026 May 18;20(5):e0013572. doi: 10.1371/journal.pntd.0013572 (PMC13193613; doi:10.1371/journal.pntd.0013572)
Supplement: Attachment — Submitted filename: Reviewer_Report_R1_PNTD-D-25-01673_submitted.pdf [file pntd.0013572.s012.pdf]

Reviewer Report: PNTD-D-25-01673R1

Manuscript Title: Dengue vaccine acceptability in Peru: A mixed-methods study in two dengue-endemic Peruvian cities

Journal: PLOS Neglected Tropical Diseases

Overall Recommendation: MINOR REVISION

Summary of Revision Assessment

I appreciate the authors' thorough and thoughtful engagement with the concerns raised in the first round of review. The revised manuscript is substantially improved. The reframing as a pre-implementation baseline study is now clearly and consistently articulated throughout the introduction, methods, and discussion. The outcome variable has been meaningfully strengthened using all seven Oxford scale items and four complementary outcome specifications. The addition of sensitivity analyses, the convergence matrix, the model-building transparency, and the VIF diagnostics are all welcome and important changes. The discussion is more nuanced and the recommendations more actionable. That said, a number of relatively minor issues remain that should be addressed before acceptance.

Assessment of Responses to Major Concerns

1. Temporal Disconnect and Reframing

Status: Adequately addressed.

The manuscript now clearly positions itself as a pre-implementation baseline throughout the introduction, methods, and discussion. The rationale for using COVID-19 vaccination as a concrete proxy, rather than discussing a purely abstract hypothetical, is well argued and persuasive. I also found the acknowledgment that a follow-up Qdenga study is underway, with preliminary indications that these findings held, to be a credible addition. No further changes needed on this point.

2. Outcome Variable Definition

Status: Substantially improved, minor clarification needed.

The shift to using all seven adapted Oxford scale items and constructing four complementary outcome specifications is a major improvement. The three-level categorical outcome for descriptive analyses (81.9% acceptors, 14.5% unsure, 3.6% refusers) is more informative than the prior binary split, and the sensitivity analyses across all four specifications (latent continuous, observed continuous, three-level categorical, and dichotomous) strengthen confidence in the findings. The rationale for focusing the multivariable model on acceptors vs. unsure, targeting the "movable middle," is reasonable and well justified.

Remaining issue: The abstract still reports 81.9% as the acceptor rate based on the revised multi-item classification, but the earlier version reported 81.2% from the single-item measure. Although

both figures are defensible within their respective operationalizations, the abstract should briefly note that this figure reflects a theory-driven, multi-item classification rather than a single survey question, so that readers do not conflate the two approaches. Additionally, Supplementary Material 2 is referenced heavily but its accessibility and clarity are critical. Please ensure that the four outcome specifications, their construction rules, and the sensitivity analysis results are presented in a format that is easy to follow, for example a summary table showing effect estimates side by side across all four specifications.

3. Education and Hesitancy Finding

Status: Improved, but still somewhat underexplored.

The revised OR (3.33 vs. the prior 6.48) and narrower confidence interval indicate better model stability, which is reassuring. The addition of a brief literature review showing mixed evidence across Latin America on this topic is helpful. The speculative explanations offered (critical appraisal, exposure to anti-vax narratives, preference for private sector access) are plausible.

Remaining issue: The discussion of this finding remains largely speculative and somewhat parenthetical in tone. For example, the rhetorical question on lines 588 to 589 reads more like a brainstorming note than a formal interpretation. I would suggest restructuring this passage so the hypotheses are stated more directly. Additionally, if any qualitative data from the FGDs touch on differences in how participants with more vs. less formal education discussed vaccine concerns, even indirectly, this would strengthen the interpretation. Even a brief statement that the FGDs did not systematically capture education-stratified perspectives, and that this represents a gap for future research, would add transparency.

4. Statistical Analysis

Status: Substantially improved.

The additions are commendable. The flow diagram (Figure 1) now clearly reports exclusions and reasons. The model-building strategy is transparent and theory-driven. VIF diagnostics are reported. And the rationale for not pursuing formal interaction terms is reasonable given the lack of descriptive heterogeneity between sites. The reduction in excluded participants from 29% to 13.8% is also notable and reflects improved model specification.

Minor points:

(a) The Table 5 footnote mentions several variables that were included in the adjusted model but are not shown. It would be helpful to confirm that the full model results, including these non-significant variables, are available in Supplementary Material 2 or 3, and to state this explicitly in the footnote.

(b) The complete-case exclusion of 90 participants due to missing covariates is acknowledged, but a brief characterization of whether these individuals differed systematically from included participants (e.g., by site, age, or education) would strengthen the argument against selection bias. Even a short supplementary table would suffice.

5. Mixed-Methods Integration

Status: Adequately addressed.

The addition of a "Comparison of Qualitative and Quantitative Findings" section in the results and the full convergence matrix in Supplementary Material 5 represent a meaningful improvement. The paragraph summarizing convergence and divergence across the 5C domains is well written and provides a balanced assessment. The honest acknowledgment that some discrepancies reflect differences in how topics were captured across methods is appropriate.

Minor suggestion: Consider adding one sentence in the convergence summary that directly addresses the trust in health professionals finding. Specifically, FGDs emphasized trust in health professionals, but this variable did not reach significance in the adjusted regression model (OR 0.55, $p = 0.057$). This was specifically highlighted in the original review as an example of a discrepancy needing explanation, and the near-significant p-value and the direction of effect deserve a brief comment.

Assessment of Responses to Moderate and Minor Concerns

Sampling and Generalizability

The acknowledgment of the overrepresentation of women and the justification that women tend to be household health decision-makers in Peru is adequate. No further changes needed.

Measurement

Reporting of Cronbach's alpha for the adapted VAX scale ($\alpha = 0.73$) is appreciated and meets expectations. The dengue knowledge operationalization is now described in Supplementary Material 2. That said, I would suggest adding a one-sentence description of the dengue knowledge variable in the main text methods section (something along the lines of *"Correct knowledge of dengue transmission was defined as identifying the bite of an infected mosquito as the mode of transmission"*) so that readers do not need to consult supplementary materials for this key variable.

Presentation and Clarity

Figure 2 (formerly Figure 1, the VAX scale diverging bar chart) appears improved. Table 3 now separates themes from quotes more clearly. These changes are satisfactory.

Discussion and Recommendations

The recommendations are now more concrete and actionable, with specific suggestions about integrating vaccination into existing outreach platforms, leveraging locally trusted figures, and addressing residual COVID-19 mistrust. The addition of the Puerto Rico comparison and the multi-country Latin American study strengthens the contextualization. Well done.

Literature Engagement

The addition of references on the heterogeneous education/hesitancy relationship and on dengue vaccine acceptance in other Latin American settings is adequate. The authors' candid note that

literature on two-dose schedule barriers or seronegativity-specific concerns for newer vaccines remains limited is fair.

New Issues Identified in the Revised Manuscript

(a) Abstract consistency. The abstract mentions "reduced confidence due to the rapid COVID-19 vaccine development (OR 2.17)" and "negative views towards COVID-19 vaccination (OR 2.17)." While it is coincidental that both ORs are 2.17, this may confuse readers into thinking it is a single finding reported twice. Consider rephrasing to make clear these are two separate predictors.

(b) Terminology. The manuscript now uses "acceptors," "unsure," and "refusers" consistently, which is good. However, the abstract uses "vaccine uncertainty" in some places and "hesitancy" in others. I would recommend settling on one term for the primary outcome throughout the abstract to avoid ambiguity. "Vaccine uncertainty" seems more precise given the operationalization.

(c) Line 103 (Butantan DV). The sentence about the Butantan DV vaccine states it "is not yet in use because it is under regulatory review and awaiting approval." Given how quickly the regulatory landscape is moving, it may be worth verifying whether this has changed since manuscript preparation.

(d) Table 5 readability. Both unadjusted and adjusted estimates are shown side by side, which is appropriate. For readability, consider bolding or otherwise visually flagging the statistically significant adjusted estimates to help readers quickly identify the key findings.

Final Recommendation

The authors have made substantial and responsive revisions that address the core methodological and conceptual concerns from the initial review. The manuscript is now better framed, more analytically rigorous, and more transparent in its methods. The remaining issues are minor and can be addressed without additional data collection or reanalysis. I **recommend acceptance after minor revision**.

Summary of required minor revisions:

1. Clarify in the abstract that the 81.9% acceptor figure reflects a multi-item, theory-driven classification.

2. Ensure Supplementary Material 2 includes a side-by-side comparison table of effect estimates across all four outcome specifications.

3. Restructure the education/hesitancy discussion to state hypotheses directly rather than as rhetorical questions, and acknowledge the qualitative gap.

4. Confirm full model results (including non-significant variables) are accessible in supplementary materials. Add a brief characterization of participants excluded due to missing data.

5. Add a sentence in the convergence summary addressing the trust in health professionals discrepancy between FGDs and regression results.

6. Add a brief in-text definition of the dengue knowledge variable in the methods section.

7. Resolve abstract-level ambiguity: clarify the two distinct OR 2.17 findings and standardize the terminology (uncertainty vs. hesitancy).

8. Minor presentation improvements to Table 5 (visual flagging of significant estimates).
