# Supplementary material for: Dengue vaccine acceptability in Peru: A mixed-methods study in two dengue-endemic Peruvian cities
Source: PLoS Negl Trop Dis. 2026 May 18;20(5):e0013572. doi: 10.1371/journal.pntd.0013572 (PMC13193613; doi:10.1371/journal.pntd.0013572)
Supplement: Attachment — Submitted filename: Reviewer Response.docx [file pntd.0013572.s011.docx]

# **SUMMARY AND GENERAL ASSESSMENT**

This is a timely and valuable mixed-methods study that provides crucial pre-implementation data on dengue vaccine acceptability in high-burden Peruvian regions, framed by the **5C model of vaccine hesitancy**. The combination of focus groups and a survey is appropriate for the research question. The finding of 81.2% **stated willingness** to be vaccinated establishes an important baseline. However, the strength of the conclusions is significantly constrained by a critical **temporal disconnect** (2022 data collection vs. 2024 Qdenga rollout) and several substantial methodological issues related to statistical rigor and outcome definition. Addressing these concerns is essential for the manuscript to provide actionable and credible guidance.

**Major Concerns Requiring Substantial Revision**

## **1. Temporal Disconnect and Research Opportunity**

The core limitation is the gap between surveying attitudes toward a **hypothetical vaccine** in 2022 and the **actual Qdenga rollout** in 2024.

- **Validity Issue:** Attitudes were likely heavily influenced by the immediate **COVID-19 context** (e.g., mandates, political polarization), which may temporarily inflate hesitancy and may not translate to real-world dengue vaccine uptake.

We acknowledge the impact of the COVID-19 context on our findings. The COVID-19 pandemic has likely had a profound impact on how vaccines are viewed worldwide, and some of the global myths and misconceptions were observed in some sub-populations in Peru. Peru had one of the highest fatality rates in the world, schools were shut down for over a year, the country was in military-regulated lockdown, and as businesses started to open (only a year before this research took place), people were only allowed into closed buildings (even small corner stores) if they showed their COVID vaccine card. That said, in our experience doing qualitative research over the past two decades in Iquitos, we have found that people are better able to discuss tangible and concrete examples over hypothetical ones. **Hence, because the pandemic was fresh for most participants, using COVID-19 vaccination as a concrete example to discuss issues related to a hypothetical dengue vaccine was purposefully done by our team to strengthen the focus group discussion.** It allowed us to probe into the range of nuanced issues that emerged for one infectious disease – COVID-19 – to discuss how this MIGHT affect decision-making for a different vaccine for another infectious disease.

We would also like to state that following the Qdenga rollout in 2024, our research team proceeded to conduct focus groups and interviews in Iquitos to examine the barriers that emerged. We are finalizing the Qdenga manuscript, but will say here that potential barriers that are discussed in this manuscript would have been helpful for health authorities, and we believe will be helpful moving forward.

To address this, we have added the following sentences to the discussion (lines 584-487):

Lines 120-122: At the time our study was conducted, no dengue vaccine was commercially available in Peru, so participant experiences with COVID-19 vaccination efforts in Peru were used to provide a concrete and tangible example for understanding public perceptions and actual behaviors.

Lines 188-190: To facilitate discussion in the FGDs, we used the recent and tangible experience with the COVID-19 vaccination as a starting point to examine acceptance towards a potential (but at the time, still hypothetical) dengue vaccine

Lines 607-621: One potential methodological limitation was our decision to explore attitudes toward a hypothetical dengue vaccine by asking participants to reflect on their decision-making process regarding another recent 'new vaccine'—the COVID-19 vaccine—as a proxy for evaluating acceptance of novel immunization strategies. Peru’s COVID-19 vaccination efforts began in February 2021 [75], only a year before our FGDs took place, which likely influenced responses by our participants, as exposure to vaccination information and campaigns were highly publicized and evolving at this time. Some may argue that the recent COVID-19 experience influenced how people responded to the FG and survey questions, and we fully agree: it is likely to influence many for a long time. But we specifically constructed our FGDs to include people’s experience with COVID-19 because we felt it was a real and tangible example of what might drive hesitancy by looking at what happened during the pandemic, rather than discussing an abstract and hypothetical future vaccine. Since this study, there was a limited rollout of the Qdenga vaccine in Iquitos, and we conducted a qualitative study to explore health professionals’ and community members’ experiences. This manuscript is in preparation, but the findings from this mixed methods study held and are still relevant to future large-scale rollouts.

- **Required Action:** The paper must be **reframed as pre-implementation baseline data**. The discussion should explicitly address how the post-COVID context may skew the findings and how the shift from a hypothetical to a real vaccine may change behavior.

This study investigated attitudes towards a hypothetical future vaccine. We have addressed this by adding terminology for “hypothetical” and “pre-implementation acceptance” in the last paragraph of the introduction and briefly in the methods (line 200).

Lines 117-119: Our study used a mixed-method approach to assess acceptability towards and barriers to uptake of a hypothetical dengue vaccine in order to propose strategies for future dengue vaccine deployment in Peru – a pre-implementation baseline that could be used to guide future rollouts.

Lines 122-125: Since then, the Qdenga vaccine was rolled out in 2024 in Iquitos and Piura (the two sites of this study), and a manuscript is in progress about post-implementation barriers and how these compared and contrasted to pre-implementation findings.

Lines 132-134: We present findings from focus group discussions and surveys conducted in two dengue-endemic Peruvian communities, exploring pre-implementation acceptability of a dengue vaccine.

Additionally, we have added the following paragraph to the discussion to address the likely influence of COVID-19 on current vaccine hesitancy:

Lines 607-621: One potential methodological limitation was our decision to explore attitudes toward a hypothetical dengue vaccine by asking participants to reflect on their decision-making process regarding another recent 'new vaccine'—the COVID-19 vaccine—as a proxy for evaluating acceptance of novel immunization strategies. Peru’s COVID-19 vaccination efforts began in February 2021 [75], only a year before our FGDs took place, which likely influenced responses by our participants, as exposure to vaccination information and campaigns were highly publicized and evolving at this time. Some may argue that the recent COVID-19 experience influenced how people responded to the FG and survey questions, and we fully agree: it is likely to influence many for a long time. But we specifically constructed our FGDs to include people’s experience with COVID-19 because we felt it was a real and tangible example of what might drive hesitancy by looking at what happened during the pandemic, rather than discussing an abstract and hypothetical future vaccine. Since this study, there was a limited rollout of the Qdenga vaccine in Iquitos, and we conducted a qualitative study to explore health professionals’ and community members’ experiences. This manuscript is in preparation, but the findings from this mixed methods study held and are still relevant to future large-scale rollouts.

- **Strong Encouragement:** The authors have an extraordinary opportunity to transform this work by committing to a rapid **follow-up validation study** to compare their 2022 predictions against 2024-25 **actual Qdenga uptake** in their study sites. This would elevate the paper's contribution to high-impact implementation science.

We did conduct a follow-up qualitative study to evaluate the roll-out of the Qdenga vaccine in Iquitos, which was made available for children 10-16 years old. In July- August 2025, we conducted focus group discussions with parents of children in the eligible age group and key informant interviews with local ministry of health professionals involved in the rollout. Findings from THIS manuscript are relevant to the findings in the upcoming manuscript and for future dengue vaccine rollouts. We currently have the Qdenga manuscript under preparation, but have added the following sentences to the introduction and the discussion:

Lines 122-125: Since then, the Qdenga vaccine was rolled out in 2024 in Iquitos and Piura (the two sites of this study), and a manuscript is in progress about post-implementation barriers and how these compared to pre-implementation findings.

Lines 605-608: Since this study, there was a limited rollout of the Qdenga vaccine in Iquitos, and we conducted a qualitative study to explore health professionals’ and community members’ experiences. This manuscript is in preparation, but the findings from this mixed methods study held and are still relevant to future large-scale rollouts.

## **2. Problematic Outcome Variable Definition**

The study's **dichotomization of vaccine acceptance** is methodologically flawed because it conflates distinct concepts, leading to potential misclassification and limited policy relevance.

The definition groups participants as "Willing" if they responded:

- "I would get it as soon as I can" (Immediate Acceptance)
- **"I would get it when I have time" (Convenience Barrier)**

The phrase **"when I have time"** strongly indicates a *logistical or convenience barrier* (e.g., work conflicts, travel distance, long clinic queues), not true **vaccine hesitancy** (which is typically defined as delay or refusal due to psychological or cognitive factors like lack of confidence or complacency).

This conflation has three major consequences:

1. **Misclassification and Inflated Rates:** It likely misclassifies participants who face access challenges as fully "willing," which artificially **inflates the overall acceptance rate** (reported at 81.2%).

We agree that convenience barriers fit within factors that would influence obtaining the vaccine or not and, by deciding to use the validated Oxford Vaccine Hesitancy Scale, we are restrained to their categorization – with all its pros and cons. However, we have tried to examine possible inflation by doing the following.

In the previous version, as you point out, dengue vaccine acceptability was operationalized using a single item adapted from the Oxford COVID-19 Vaccine Hesitancy Scale, in which responses “I would get it as soon as I can” and “I would get it when I have time” were grouped as willing or acceptors. In the revised manuscript, we NOW incorporate all seven items of the adapted Oxford scale to construct four complementary outcomes, three that are validated by the Oxford scale (latent continuous, observed continuous, three-level categorical) and one created by us (dichotomous acceptor vs. unsure) (see supplementary material 2). For descriptive analyses, we now use the three-level outcome, yielding: 81.9% acceptors, 14.5% unsure, and 3.6% refusers. For multivariable analyses, we focus on acceptors versus unsure, to examine determinants of vaccine uncertainty within the “movable middle,” excluding the refusers group due to its small size and conceptual distinctiveness. This dichotomous variable might still inflate “acceptors” by including those who may forego vaccination if there are convenience barriers, so we have added a line about this in the discussion section in lines 566-568: dengue vaccination programs should be designed to minimize logistical burdens, particularly for those might accept the vaccination but may delay or miss getting it due to access constraints.

2. **Obscured Policy Relevance:** By treating convenience barriers as acceptance, the analysis **obscures critical logistical hurdles** that implementation strategies must address (e.g., mobile clinics, evening hours).

Agree with comment and responded to this issue above. That said, in the qualitative findings, convenience barriers came out as FGD participants discussed COVID-19 vaccine and hence the issue of convenience is still highlighted as a policy implication from this article. We discuss convenience as a key issue, yet a modifiable one, that can potentially be minimized through implementation in existing public health infrastructure.

Lines 556-568: Convenience also emerged as a key and modifiable factor that could shape vaccine uptake. Although participants did not describe long waiting times as a reason to refuse vaccination, various experiences during the COVID-19 vaccination were described as deterrents, including extended lines, limited vaccination hours, and transportation barriers. These findings suggest that dengue vaccination programs should be designed to minimize logistical burdens, particularly for those might accept the vaccination but may delay or miss getting it due to access constraints…

3. **Loss of Nuance:** Converting a nuanced, multi-point scale into a simple binary variable sacrifices valuable information about the continuum of acceptance and refusal.

As mentioned above, we agree with this point, and yet are also trying to rely on the validated Oxford scale. Also as mentioned above, we have conducted sensitivity analyses (seen in supplementary material 2) using all four outcome definitions with an identical, theory-driven set of predictors aligned with the 5C’s model (confidence, complacency, convenience, communication, and context). Across these alternative operationalizations—including continuous and categorical approaches—the direction and relative magnitude of associations were highly consistent. This convergence strengthens confidence that the substantive conclusions are robust and not an artifact of dichotomization (see supplementary material 2).

We have made the following changes in lines 306-309: Additionally, sensitivity analyses were conducted using alternative outcome specifications (three-level categorical, latent continuous, and observed continuous outcomes), applying the same predictor set; these results are presented in Supplementary Material 2 [S2].

For descriptive analyses, we now use the three-level categorical outcome, which preserves conceptual distinctions between acceptance, uncertainty, and refusal. For multivariable modeling, we selected the acceptors versus the unsure specification to examine determinants of vaccine uncertainty among individuals who do not categorically reject vaccination – and hence, from a public health perspective, more “movable” towards acceptance.

**Required Changes:**

The authors must either:

1. **Conduct Sensitivity Analyses:** Test alternative models by treating "I would get it when I have time" as a separate category, or by grouping it with "I would delay getting it" (a form of logistical hesitancy).

In the revised manuscript, we conducted sensitivity analyses based on four outcome specifications derived from the seven items of the adapted Oxford Vaccine Hesitancy Scale. Specifically, we ran multivariable regression models for each of the four outcomes using the same set of predictors as in the final model. Across these parallel analyses, the direction and relative magnitude of associations were consistent, indicating that the findings are robust to alternative operationalizations of vaccine hesitancy. Full details of these sensitivity analyses are presented in supplementary material 2 and additional methodological details were added in the methods section.

2. **Treat as Ordinal:** Analyze the outcome as an **ordinal variable** (preserving all response categories) to capture the full nuance of participant intent and proximity to refusal.

We did not treat it as ordinal, but did conduct the sensitivity analysis and also provided a clear justification for this decision. We also added a statement about this in the manuscript:

Lines 228-232: In the main manuscript, descriptive analyses are presented using the three-level categorical outcome to preserve meaningful gradients of vaccine acceptability. However, multivariable regression analyses focus on the dichotomous outcome, selected to identify determinants of vaccine uncertainty among individuals potentially amenable to intervention. Detailed descriptions of outcome construction and sensitivity analyses across all specifications are provided in Supplementary Material 2 [S2].

3. **Justify Categorization:** If the current categorization is retained, provide a **clear, evidence-based justification** by referencing established, validated vaccine acceptance scales.

The dichotomous specification (acceptor vs. unsure) was retained for the primary multivariable analysis for implementation-oriented purposes, focusing on determinants of vaccine uncertainty within the “movable middle.” Importantly, sensitivity analyses using three-level and continuous outcome specifications yielded consistent direction and magnitude of associations, supporting the robustness and validity of the retained categorization.

## **3. Unexplained and Counterintuitive Finding: Education and Hesitancy**

The finding that **technical/higher education is associated with dramatically increased hesitancy (OR 6.48)** is highly unusual and requires deep, data-driven exploration.

- **Problem:** The provided discussion is insufficient. This large effect could signal confounding, selection bias, or a genuine phenomenon (e.g., greater exposure to online misinformation or distrust among critical, educated groups).
- **Required Analysis:** Provide a **deeper analysis** exploring mechanisms: test for **interaction effects** with information sources and trust measures and systematically integrate **qualitative data mining** to find education-specific themes.

In the original version, the adjusted odds ratio for technical/higher education (AOR 6.48; 95% CI 2.24–21.4) was likely influenced by overadjustment and model instability. In the revised manuscript, we implemented a structured, theory-driven covariate selection process grounded in the 5C’s framework and reassessed multicollinearity. In the updated final model, higher education remains positively associated with *being unsure to get vaccinated* (AOR 3.33; 95% CI 1.31–8.97), but with a more conservative effect size and narrower confidence interval, suggesting improved model stability.

Importantly, the direction and relative magnitude of this association were consistent across sensitivity analyses using alternative outcome specifications (three-level categorical, latent, and observed continuous outcomes; supplementary material 2), reducing concern that the finding is an artifact of a single modeling approach.

A literature review of vaccine hesitancy and education level in Latin America demonstrated there is not a single consensus on the subject. There are some studies that found a higher level vaccine hesitancy in groups with higher education levels (like our findings), some that found a higher level of vaccine hesitancy in groups with lower education levels, and some that found no significant relationship between the two variables. Based on our experience on site, we believe that it is possible that more educated participants expressed greater scrutiny of vaccine approval processes, institutional transparency, and information sources, rather than categorical rejection. In this case, higher education may be associated with more critical engagement (they might have been more aware of the “anti-vax” movement in other countries). We added this sentence in the discussion section:

Lines 580-592: Our findings also reveal that vaccine hesitancy is not evenly distributed across the population, suggesting opportunities for targeted interventions. The literature examining the relationship between educational attainment and vaccine acceptance is heterogeneous, with some studies reporting greater hesitancy among individuals with higher education, others identifying higher hesitancy among those with lower education, and several observing no significant association between education and vaccine attitudes [65,68–70]. Our study found individuals with higher levels of formal education were more likely to express uncertainty, underscoring that hesitancy in this context may reflect critical appraisal rather than lack of information (e.g., is it possible that those with higher education in these settings were more influenced by myths that emerged amongst vaccine deniers globally during the pandemic?)– or the ability to access services including vaccines from sources this population trusts more, such as the private sector, even if it requires paying. More research would be needed to explore this issue.

## **4. Statistical Analysis Deficiencies (Critical)**

Confidence in the final model's predictors is undermined by several critical reporting omissions:

- **Missing Data:** The final model excludes **29% of participants** without any description of missing data patterns, handling strategy (e.g., imputation), or sensitivity analyses. This raises a significant risk of selection bias. A **missing data flow diagram** is required.

We provide a data flow diagram as Figure 1 that reports the number of individuals assessed, excluded, enrolled, and included in the final multivariable analysis, including site-specific counts and reasons for exclusion.

In the revised manuscript, we strengthened the construction of the final multivariable model by applying an a priori, theory-driven variable selection aligned with the 5C’s framework, which reduced reliance on predictors with substantial item-level missingness that contributed to attrition in the previous version. In the current final analysis, 13.8% of enrolled participants were excluded (122/883), primarily due to: (i) exclusion of participants classified as “vaccine refusers” (n = 32) for the binary outcome specification focused on uncertainty (acceptor vs. unsure), and (ii) complete-case exclusion due to missingness in covariates included in the final model (n = 90).

- **Model Building:** The process for variable selection is unclear. The authors must provide a **transparent description of the model building strategy** and justify variable inclusion/exclusion.

We have added much more detail about variable selection and model building in our methods section. Specifically:

Line 290-309: Selection of predictors for the final multivariable model followed a structured, theory-driven approach grounded in the 5C’s framework. All candidate variables were classified a priori into the five conceptual domains (Confidence, Complacency, Convenience, Communication, and Context). Within each domain, multicollinearity was assessed using variance inflation factors (VIF) and overlapping or highly correlated variables were evaluated to guide retention or exclusion. Crude and domain-specific adjusted models were examined to assess the stability of effect direction and relative magnitude within each domain. In addition to domain-specific predictors, a core set of a priori sociodemographic covariates (sex, age group, education level, and occupation) was included in the final multivariable model to control for potential confounding. These covariates were retained irrespective of statistical significance. Final inclusion of predictors in the multivariable model was guided by conceptual relevance, absence of problematic multicollinearity, consistency of effects across domain-specific analyses, and model parsimony. Variables were not selected solely based on statistical significance; conceptually important predictors were retained even when not statistically significant to ensure adequate representation of key dimensions of vaccine hesitancy. All variables evaluated within each domain, the set of predictors retained in the final model, and the results of multicollinearity diagnostics are presented in Supplementary Material 3 [S3]. Additionally, sensitivity analyses were conducted using alternative outcome specifications (three-level categorical, latent continuous, and observed continuous outcomes), applying the same predictor set; these results are presented in Supplementary Material 2 [S2].

- **Model Diagnostics: VIF statistics** are necessary to check for multicollinearity among correlated variables (e.g., education, wealth, residence).

We can confirm that multicollinearity was systematically assessed. For each domain, we estimated variance inflation factors (VIF) in multivariable logistic regression models. This approach allowed us to identify and exclude redundant variables.

All variables retained in the final model demonstrated acceptable VIF values (VIF > 2), indicating no evidence of problematic multicollinearity, including among sociodemographic variables such as education, occupation, and place of residence. This diagnostic process ensured the stability and interpretability of the regression estimates. Detailed VIF results have been added to the supplementary material 3 to provide full transparency of the diagnostic process.

Specifically, in lines 293-295m, we state: Within each domain, multicollinearity was assessed using variance inflation factors (VIF) and overlapping or highly correlated variables were evaluated to guide retention or exclusion.

- **Effect Modification:** Given the substantial difference in hesitancy between sites (Piura 13.8% vs. Iquitos 23.8%), **interaction terms (site × predictor)** must be tested to explore effect modification.

In the revised manuscript, site-specific differences in dengue vaccine hesitancy were re-examined using the updated three-level categorical outcome. In contrast to the previous dichotomous specification, no statistically significant differences were observed between Iquitos and Piura in the distribution of accepting, unsure, and refusing participants (p = 0.5), and absolute differences across categories were modest. Furthermore, study site was included as a covariate in the final multivariable logistic regression model comparing willing versus unsure participants and was not significantly associated with vaccine uncertainty after adjustment. Given the absence of both descriptive heterogeneity and adjusted associations and lacking a strong theoretical basis for differential effects by site, formal interaction terms (site × predictor) were not pursued. Site was retained as an adjustment variable to account for contextual differences.

- **Clarity:** Ensure **consistent interpretation** of Odds Ratios (ORs) throughout the abstract and results (i.e., always clearly state if the OR represents hesitancy or acceptance).

We have read throughout the paper and revised our OR interpretations to ensure they clearly state what they represent, as well as made our language clearer to specify the three groups: acceptors, unsures, and refusers.

## **5. Weak Integration of Mixed Methods**

The study describes itself as mixed methods, but the integration is primarily sequential (qual → quant) rather than analytical.

- **Required Action:** Use the qualitative data more effectively to **explain contradictory quantitative findings**. For instance, why did FGDs reveal trust in health professionals if this was not a significant predictor in the regression model? A **convergence matrix or joint display** is needed to systematically compare and reconcile qualitative and quantitative results.

We appreciate this suggestion and created such a matrix. It was so long, however, that we present in the article a paragraph summarizing this matrix, and have added a table detailing the convergence matrix of mixed-methods findings: “Convergence Matrix of Mixed-Methods Findings” in supplementary material 5. Some of the differences in contradictory findings are due to how certain topics were asked – so not easy to compare findings systematically, but at least allow for a summary of the main points that arose in qualitative and quantitative. Here is the paragraph added in results:

Lines 505-523: Qualitative and quantitative findings were largely convergent. Both data sources demonstrated high acceptability of a potential dengue vaccine, with confidence—shaped by trust, transparency, and prior vaccination experiences—emerging as a central determinant. Strong convergence was also observed in the domain of communication: participants emphasized the importance of disseminating information through multiple media channels (e.g., radio, loudspeakers, social media) and from trusted sources, including health professionals and the Ministry of Health. Misinformation and residual mistrust related to the COVID-19 vaccination experience emerged in both datasets, underscoring the need for clear and consistent messaging. FGDs, however, provided more detailed insight into informational needs, including safety, side effects, eligibility criteria, number of doses, and prior vaccine use. In the domain of convenience, logistical barriers such as waiting times were not described as deterrents in either FGDs or surveys, yet cost emerged as a salient factor; willingness to pay was lower among participants classified as unsure, and FGD participants generally expected vaccination to be free. For complacency, qualitative findings emphasized lived experience with dengue as a motivator, whereas survey data highlighted perceived risk and knowledge of dengue transmission as factors associated with acceptance. Finally, contextual findings were complementary: FGDs identified Indigenous and certain religious communities as potentially harder to reach, while survey analyses identified sociodemographic correlates of vaccine uncertainty. A detailed matrix comparing qualitative and quantitative findings is provided in Supplementary Material 5 [S5].

## **6. Moderate and Minor Concerns**

- **Sampling and Generalizability:** The **daytime household recruitment** resulted in an oversampling of women (**71% female**) and likely under samples working-age males. The authors must acknowledge this **selection bias** and discuss its implication for generalizability.

One reason we oversampled females is they are generally responsible for health decisions for their children. We have added the following line to our limitations section in lines 635-638: Finally, almost three quarters of our survey participants were women, which is not representative of the whole population and may affect generalizability of the study, yet in our experience women tend to be the decision makers about health and vaccines in their families in Peru.

- **Measurement:** The adaptation of the VAX scale from 7-point to 5-point without validation requires reporting of **reliability statistics** (e.g., Cronbach's alpha). The operationalization of the "dengue knowledge" measure must be fully described.

In the revised manuscript, we now report reliability statistics for the adapted Vaccination Attitudes Examination (VAX) scale. Internal consistency of the 12-item VAX scale adapted from a 7-point to a 5-point Likert format was acceptable in our sample (Cronbach’s α = 0.73; standardized α = 0.72; complete-case N = 872).

Lines 223-227: After piloting this instrument with 20 individuals, the response format was adapted from a 7-point to a 5-point Likert scale for comprehension, tested for internal consistency (Cronbach’s α = 0.73; standardized α = 0.72). The survey was developed, collected, and managed using REDCap (version 14.0.8) electronic data capture tools [60,61] and applied by our team on tablets [S1].

Regarding dengue knowledge, we have clarified its operationalization and presentation. All variables considered under the 5C’s vaccine hesitancy framework—including knowledge-related indicators—are now fully described and grouped by domain in supplementary material 2. This material also explicitly identifies which variables were retained in the final multivariable model, improving transparency and reproducibility.

We added reliability analyses for the adapted 5-point VAX scale, including Cronbach’s alpha for the full scale, reported in the methods. We also expanded the description of dengue knowledge and other predictors aligned with the 5C’s framework. A new table in supplementary material 3 details all candidate variables by domain and clearly indicates those included in the final multivariable model, improving clarity of measurement and analytic decisions.

- **Presentation and Clarity: Figure 1** is difficult to read and should be redesigned. **Table 3** should clearly separate analytical themes from illustrative quotes.

We have redesigned Figure 1 (now Figure 2) to make it easier to read: Figure 2. Participant responses to Vaccination Attitudes Examination (VAX) Scale.

We have modified Table 3 slightly, but we are not sure if this addresses the concern. We have included now one column about themes and one on illustrative themes.

- **Discussion and Recommendations:** The recommendations must be more **concrete, context-specific, and actionable** (e.g., specifying messaging for the two-dose schedule or how to target the concerns of higher-educated individuals).

We have updated our recommendations throughout the discussion section to make them more concrete and actionable, including explicitly addressing transparency around vaccine information, residual mistrust from COVID-19, minimizing logistical burdens, and integrating the dengue vaccine into existing outreach platforms.

Specifically, in lines 542-546: These results highlight that dengue vaccination programs will need to explicitly acknowledge and address possible residual mistrust stemming from the COVID-19 pandemic, rather than assuming that dengue vaccines would be evaluated by the population independently of this other recent vaccination experience.

Lines 547-555: Programmatically, our findings imply that communication strategies should emphasize transparency around the dengue vaccine development, safety monitoring, eligibility criteria, number of doses, and expected side effects. This finding aligns with findings from Puerto Rico that found that inconsistent information, concerns about vaccine side effects and risks, and multiple doses were key barriers to Dengvaxia acceptance [66]. Participants emphasized the importance of receiving information from trusted health professionals and institutions, including the Peruvian Ministry of Health, suggesting that integrating dengue vaccination into clinical services – whether facility-based or through outreach to remote communities – may strengthen confidence. Consistently, a multi-site study conducted in Latin America (that included Argentina, Brazil, Colombia, and Mexico) and several Asian countries found higher willingness to vaccinate among individuals who expressed trust in the health system and in their health professionals’ ability to manage any side effects [64,65]. Leveraging the support of locally trusted figures as program champions may further enhance acceptability, particularly when these individuals are visibly vaccinated themselves [67].

And lines 569-573: Integrating dengue vaccination into existing outreach platforms may offer an efficient solution that builds on existing infrastructure and services that are already trusted and used. For example, coupling vaccination with established vector control activities or mobile health campaigns could reduce vaccine access barriers while capitalizing on familiar public health infrastructure.

- **Literature Engagement:** Strengthen the discussion of existing **Latin American dengue vaccine acceptance literature** and address factors specific to the new vaccine, such as the two-dose schedule and seronegativity approval.

Although, we didn’t find any literature specifically addressing a two-dose schedule or seronegativity approval related to a new dengue vaccine (this literature exists for Dengvaxia), there were various studies that look at similar factors of dengue vaccine acceptance as ours. We reviewed qualitative and larger quantitative studies investigating general dengue vaccine acceptance across Latin America. We added to the following to the discussion:

Lines 580-592: Our findings also reveal that vaccine hesitancy is not evenly distributed across the population, suggesting opportunities for targeted interventions. The literature examining the relationship between educational attainment and vaccine acceptance is heterogeneous, with some studies reporting greater hesitancy among individuals with higher education, others identifying higher hesitancy among those with lower education, and several observing no significant association between education and vaccine attitudes [65,68–70]. Our study found individuals with higher levels of formal education were more likely to express uncertainty, underscoring that hesitancy in this context may reflect critical appraisal rather than lack of information (e.g., is it possible that those with higher education in these settings were more influenced by myths that emerged amongst vaccine deniers globally during the pandemic?)– or the ability to access services including vaccines from sources this population trusts more, such as the private sector, even if it requires paying. More research would be needed to explore this issue.

## **7. Recommendations for Resubmission**

The paper must address the following points with accompanying new analysis and transparent reporting:

1. **Reframing and Temporal Acknowledgment** (Line 88-98). Reframe as pre-implementation baseline; explicitly discuss the COVID-19 proxy issue and the need for validation.

We have addressed this by adding terminology in the introduction, methods, and discussion stating that this is pre-implementation of a vaccine. In both the introduction and discussion we explain why we used the COVID-19 vaccine as a proxy.

Specifically, lines 120-122: At the time our study was conducted, no dengue vaccine was commercially available in Peru, so participant experiences with COVID-19 vaccination efforts in Peru were used to provide a concrete and tangible example for understanding public perceptions and actual behaviors.

Lines 613-621: …Some may argue that the recent COVID-19 experience influenced how people responded to the FG and survey questions, and we fully agree: it is likely to influence many for a long time. But we specifically constructed our FGDs to include people’s experience with COVID-19 because we felt it was a real and tangible example of what might drive hesitancy by looking at what happened during the pandemic, rather than discussing an abstract and hypothetical future vaccine. Since this study, there was a limited rollout of the Qdenga vaccine in Iquitos, and we conducted a qualitative study to explore health professionals’ and community members’ experiences. This manuscript is in preparation, but the findings from this mixed methods study held and are still relevant to future large-scale rollouts.

2. **Outcome Definition Revision/Justification** (Line 223-228). Conduct sensitivity analyses for the definition; justify or revise categorization of "when I have time."

We have updated our manuscript to incorporate all seven items of the adapted Oxford COVID-19 Vaccine Hesitancy Scale to construct four complementary outcomes. We conducted sensitivity analyses for all four of these outcomes, which can be found in supplementary material 2. As explained previously, however, we retained the three-level outcome for descriptive analyses (acceptors, unsure, and refusers) and a policy-oriented dichotomous outcome (willing vs. unsure) for multivariable modeling.

3. **Complete Statistical Methods.** Address missing data (29% excluded), provide model building transparency, report VIFs, and test for site × predictor interaction (effect modification).

Our model building and VIFs can be found in supplementary materials 2 and 3, which detail the description of the development and selection of dengue vaccine hesitancy outcomes and variable selection and multicollinearity assessment (including VIF results), respectively. Finally, site was re-examined descriptively and in adjusted models and showed no significant association with the outcome; therefore, formal interaction terms were not pursued.

4. **Deeper Analysis of Education-Hesitancy Link.** Deeper analysis, including qualitative integration and stratified analysis, to explain the high OR for hesitancy.

As mentioned above, we have re-run the analysis with our new scale and still found education to be a positive predictor for hesitancy. While education level was not a main subject of our qualitative discussions, we added context based on our experience, but also recognize more research would be needed to understand the findings. We have added this to the discussion.

Lines 580-592: Our findings also reveal that vaccine hesitancy is not evenly distributed across the population, suggesting opportunities for targeted interventions. The literature examining the relationship between educational attainment and vaccine acceptance is heterogeneous, with some studies reporting greater hesitancy among individuals with higher education, others identifying higher hesitancy among those with lower education, and several observing no significant association between education and vaccine attitudes [65,68–70]. Our study found individuals with higher levels of formal education were more likely to express uncertainty, underscoring that hesitancy in this context may reflect critical appraisal rather than lack of information (e.g., is it possible that those with higher education in these settings were more influenced by myths that emerged amongst vaccine deniers globally during the pandemic?)– or the ability to access services including vaccines from sources this population trusts more, such as the private sector, even if it requires paying. More research would be needed to explore this issue..

5. **Systematic Mixed-Methods Integration**. Create a **joint display** or convergence matrix to systematically compare and explain discrepancies between qualitative and quantitative findings.

As suggested, we created a convergence matrix and summarized it in a “Comparison of Qualitative and Quantitative Findings” section in the results. The table, “SM5. Convergence Matrix of Mixed-Methods Findings,” was long, and we decided to place it as supplementary material 5. Finally, we acknowledge that some of the discrepancies are due to specific wording in the qualitative versus quantitative data (which may not be a true comparison), but this matrix does allow us to have a more comprehensive view on the cohesion of our data.

# **REVIEWERS' COMMENTS**

## **Methods**

Reviewer #1: - There is no sample size justification

This study was designed as a pre-implementation assessment of acceptability of a potential dengue vaccine in two endemic settings. At the time of study design, no site-specific data (including all Peru) on dengue vaccine acceptability were available, precluding a formal power calculation based on expected prevalence or effect sizes.

Therefore, sample size was determined a priori using a conservative precision-based approach commonly applied in exploratory and implementation-oriented studies, and described in lines 256-263: For the quantitative phase, a base sample of ≥400 participants per study site was defined by selecting city blocks randomly, followed by systematic door to door recruitment of subjects with a maximum number of enrollees per block. Only one participant per household was permitted (see below). This target ensured stable site-specific estimates, adequate precision for a binary outcome under a conservative assumption (±5% margin of error at 95% confidence), and feasibility for door-to-door household surveys. We used random sampling to select blocks in both the intervention and control areas to target recruitment in every household within those blocks.

Reviewer #3: The method is approperiate but further clarifications are required on the following:

- More information on the logistic regression analyses is required and particularly, how the final model was chose and how confident are you of the model fit.

In the revised manuscript, the final multivariable model was selected using a structured, theory-driven approach grounded in the 5C framework. Variables were retained based on conceptual relevance, absence of problematic multicollinearity (assessed using VIF), stability of effect estimates across domain-specific models, and parsimony. Detailed results can be found in supplementary material 3.

Lines 282-289 in the methods: For multivariable analyses, the primary outcome was a dichotomous dengue vaccine hesitancy variable contrasting “acceptors” versus with those who were “unsure”. This dichotomy was chosen to identify determinants of vaccine hesitancy among individuals who had not expressed unequivocal refusal that might provide insights about moving them from the “unsure” to the “acceptor” category. Multivariable logistic regression models were estimated to assess the association between predictors derived from the 5C’s framework and the likelihood of being unsure about vaccination. Adjusted odds ratios (ORs) and 95% confidence intervals were reported.

- What form of analysis was performed to the qualitative research- ? thematic analysis.

We have now specified we used thematic analysis in the methods.

Line 272: We conducted thematic analysis using the 5C’s.

- No sample size calculation for qualitative research was provided!

We have now specified we conducted FGDs until saturation was reached in the methods. Our sample size was determined by staff and budget resources to be 16 FGDs, which we felt would be sufficient to reach saturation within the populations.

Lines 251-252: We conducted 16 focus group discussions (FGDs) between February and March 2022 to reach saturation.

- Further information on the qualitative research is required- interview guide, probing question... etc

Under our Data Collection Instruments section of the methods, we outline the questions/ talking points from the interview guide. Our interview guide can now be found in supplementary materials 1.

Lines 188-195: To facilitate discussion in the FGDs, we used the recent and tangible experience with the COVID-19 vaccination as a starting point to examine acceptance towards a potential (but at the time, still hypothetical) dengue vaccine, as participants had all recently been engaged in this COVID-19 national vaccination program. Participants were then asked to envision a scenario where the Ministry of Health had approved a dengue vaccine and addressed three key questions: 1) What information would you need to accept vaccination? 2) How can public confidence in the vaccine be improved? 3) What is the best way to distribute a new vaccine quickly in your community?

- Not sure about the utility of a control group in qualitative and quantitative research here! No clear presentation of their data in the results.

The intervention/control classification was defined at the community level to explore whether prior exposure to dengue-related research activities might influence vaccine perceptions. As we now added in lines 243-246: We hypothesized that a community’s previous interaction with dengue research teams – and in Piura, a dengue vaccine trial – might influence responses, so we wanted to ensure some participants were selected from “intervened” areas.

Importantly, participants themselves were not necessarily enrolled in those previous studies. In both the qualitative and quantitative phases, no meaningful differences were observed between communities with and without prior dengue research exposure. In the quantitative analyses, this variable was not associated with dengue vaccine hesitancy in descriptive or multivariable models (Table 4, and Table 5).

Lines 449-451: Study site and previous participation in dengue research did not have an impact on survey participants’ vaccine acceptability.

## **Results**

Reviewer #1: (No Response)

Reviewer #2: (No Response)

Reviewer #3: Clear presentation of results, but see comment in the "methods"

## **Conclusions**

-Are the conclusions supported by the data presented?
-Are the limitations of analysis clearly described?
-Do the authors discuss how these data can be helpful to advance our understanding of the topic under study?
-Is public health relevance addressed?

Reviewer #1: (No Response)

Reviewer #2: (No Response)

Reviewer #3: Approperiate

## **Editorial and Data Presentation Modifications?** Use this section for editorial suggestions as well as relatively minor modifications of existing data that would enhance clarity. If the only modifications needed are minor and/or editorial, you may wish to recommend “Minor Revision” or “Accept”.

Reviewer #1: Minor Revision

Reviewer #2: (No Response)

Reviewer #3: Minor revision

## **Summary and General Comments** Use this section to provide overall comments, discuss strengths/weaknesses of the study, novelty, significance, general execution and scholarship. You may also include additional comments for the author, including concerns about dual publication, research ethics, or publication ethics. If requesting major revision, please articulate the new experiments that are needed.

Reviewer #1: (No Response)

Reviewer #2: (No Response)

Reviewer #3: A well-written manuscript, it would benefit from comments in "methods" and possible shortening of of some sections (a long manuscript)
